# Virtual reality as a tool to explore multisensory processing before and after engagement in physical activity

**DOI:** 10.3389/fnagi.2023.1207651

**Published:** 2023-11-02

**Authors:** Aysha Basharat, Samira Mehrabi, John E. Muñoz, Laura E. Middleton, Shi Cao, Jennifer Boger, Michael Barnett-Cowan

**Affiliations:** ^1^Department of Kinesiology and Health Sciences, Faculty of Health, University of Waterloo, Waterloo, ON, Canada; ^2^Department of Systems Design Engineering, Faculty of Engineering, University of Waterloo, Waterloo, ON, Canada; ^3^Research Institute for Aging, Waterloo, ON, Canada

**Keywords:** aging, audiovisual integration, physical activity, multisensory, virtual reality

## Abstract

**Introduction:**

This pilot study employed a non-randomized control trial design to explore the impact of physical activity within a virtual reality (VR) environment on multisensory processing among community-dwelling older adults.

**Methods:**

The investigation compared both chronic (over 6 weeks) and acute effects of VR-based physical activity to a reading control group. The evaluation metrics for multisensory processing included audiovisual response time (RT), simultaneity judgments (SJ), sound-induced flash illusion (SIFI), and temporal order judgments (TOJ). A total of 13 older adults were provided with VR headsets featuring custom-designed games, while another 14 older adults were assigned to a reading-based control group.

**Results:**

Results indicated that acute engagement in physical activity led to higher accuracy in the SIFI task (experimental group: 85.6%; control group: 78.2%; *p* = 0.037). Additionally, both chronic and acute physical activity resulted in quicker response times (chronic: experimental group = 336.92; control group = 381.31; *p* = 0.012; acute: experimental group = 333.38; control group = 383.09; *p* = 0.006). Although the reading group showed a non-significant trend for greater improvement in mean RT, covariate analyses revealed that this discrepancy was due to the older age of the reading group.

**Discussion:**

The findings suggest that immersive VR has potential utility for enhancing multisensory processing in older adults. However, future studies must rigorously control for participant variables like age and sex to ensure more accurate comparisons between experimental and control conditions.

## Introduction

Physical activity has been consistently shown to play a crucial role in maintaining and improving cognitive and perceptual processes. This is particularly relevant for older adults, as illustrated in foundational studies by Colcombe and Kramer (2003). Perceptual processes like multisensory integration—the ability to combine and interpret sensory information from various sources such as vision, hearing, and touch—are enhanced by regular physical exercise. This leads to better cognitive function, motor learning, and overall well-being (Colcombe and Kramer, 2003; Hillman et al., 2008).

The majority of research on the effects of physical activity on cognition involves traditional exercise forms like biking and treadmill running. Studies often span several months and focus on older adults, with or without cognitive impairment. For instance, in a landmark study by Colcombe et al. (2006), they found significant increases in brain gray and white matter among aerobic exercise participants, particularly in regions tied to cognitive functions like attention and memory. A follow-up study (Erickson et al., 2011) established a direct link between aerobic exercise and memory improvements.

However, most research has focused on the chronic effects of exercise. Yet, single bouts of aerobic exercise also hold promise for improving cognitive and perceptual function (Davranche and Audiffren, 2004; Davranche and Pichon, 2005; Lambourne et al., 2010; Chang et al., 2012; Pontifex et al., 2019). Specifically, limited studies have investigated how aerobic exercise influences multisensory processing in older adults. One seminal study by O’Brien et al. (2017) found that after 60–80 min of aerobic exercise, sensitivity to the Sound Induced Flash Illusion (SIFI) increased, particularly when the aerobic activity was unpredictable.

The underlying mechanisms for these effects are thought to be multifaceted. Changes in arousal levels, indicated by metrics like heart rate and skin conductance, are often cited (Lambourne et al., 2010; Chang et al., 2012; Pontifex et al., 2019). Other physiological factors such as the production of neurotrophic factors like BDNF, IGF-1, and VEGF also play roles in cognitive and perceptual function enhancement (Cotman et al., 2007; Chang et al., 2012; Pontifex et al., 2019).

The COVID-19 pandemic highlighted the need for alternative physical activity options. This is particularly crucial for older adults, who faced disruptions to their exercise routines due to closures of fitness facilities. Exergames, or interactive games that combine physical activity and gaming, have thus emerged as a viable alternative. Virtual reality (VR) exergaming (Šlosar et al., 2022) can offer an especially immersive experience and has shown promise in enhancing physical activity, motor learning, cognitive function, and emotional well-being in older adults (Miller et al., 2014; Molina et al., 2014; Amorim et al., 2019; Campo-Prieto et al., 2021; Yen and Chiu, 2021).

Yet, there is limited research on the effects of VR-based physical activity on sensory integration processes in older adults. Merriman et al. (2015) studied balance training using a VR display that was placed approximately 2 M away from the participant (i.e., non-immersive environment) and found a correlation between improved balance and susceptibility to the SIFI. Given the potential risks associated with aging, such as falling and poor decision-making due to perceptual processing changes (Setti et al., 2011a; Donoghue et al., 2014; Merriman et al., 2015), VR interventions may offer unique benefits.

In light of these gaps, our project aims to extend the evidence related to the effects of acute and long-term physical activity in a VR setting on perceptual processes. Specifically, we hypothesize that a 6-week VR the intervention will positively impact multisensory integration processes. These hypotheses are based on previous research suggesting both acute and chronic exercise can influence these processes (O’Brien et al., 2017; Basharat and Barnett-Cowan, 2023).

To this end, we developed an immersive VR physical activity intervention called Seas the Day, specifically tailored to the needs and preferences of older adults. This pilot study assesses the impact of this intervention on multisensory integration, aiming to contribute to our understanding of how alternative forms of exercise can benefit cognitive and perceptual function in older adults.

## Methods

This study is based on data from a larger pilot, non-randomized controlled trial that assessed the effects of a VR physical activity game on cognition, perception, mental well-being, changes in physical activity behaviour outside of the game, and game experience in community-dwelling older adults. Primary outcomes reflect the feasibility of a VR intervention that engaged upper extremity movement and assessments to evaluate its effects on cognition and perception. Effectiveness analyses presented below are exploratory. Assessments were conducted both before and after the 6-week intervention period (chronic effects) and before and after game plan on several days within the intervention period (acute effects).

### Intervention: VR hardware and software

**Figure 1:**
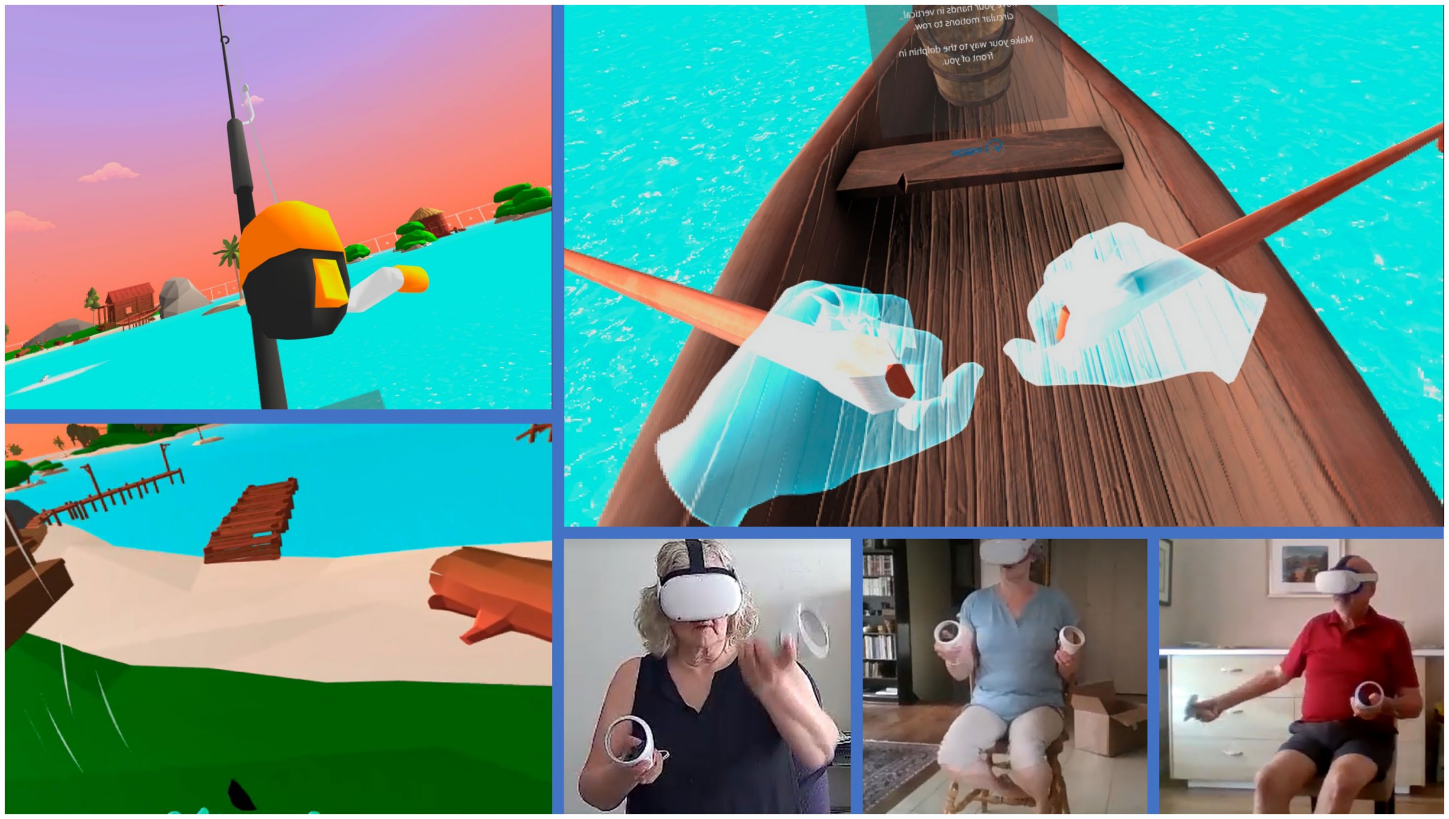
Screenshots of Seas the Day, a VR intervention to promote exercise among older adults.

Seas the Day^1^ is a custom-made VR intervention co-created to promote exercise among older adults. The game is publicly available and has been designed to foster an enjoyable physical activity session using a Tai-Chi routine, boat rowing task, and fishing. Seas the Day requires the use of the standalone VR headset, Oculus Quest 2 and the entire experience lasts 15–20 min. Generally and for the purpose of this study, the headset was an all-in-one solution for participants to engage with the VR games, through the use of two controllers. Seas the Day was designed to be played in a seated position to prevent falls as shown in Figure 1.

### Intervention: reading

Participants read from a physical book for 15–20 min in the comfort of their homes. Each participant read what they felt was interesting and engaging. When inquired about the content of the materials participants read, none revealed it to be content that increased their heart rate or their level of anxiety. Informally, participants reported the activity to be relaxing and engaging.

### Participants

The study included a convenience sample of 13 participants in the experimental (physical activity intervention) group and 14 participants in the control (reading) group, all of whom were community-dwelling older adults with or without cognitive impairment. Participants were recruited through various sources, including the Waterloo Research in Aging Pool (WRAP), the Centre for Community, Clinical and Applied Research Excellence (CCCARE) mailing list, professional networks, and personal social media accounts.

The inclusion criteria were as follows: aged 60 years or older, able to provide consent, able to complete the Montreal Cognitive Assessment (MoCA) with a score of 18 or higher, able to communicate verbally in English, able to participate in light-to-moderate unsupervised activity without requiring medical approval, ability to access a laptop or desktop PC, and access to internet at their residence. Participants were excluded based on criteria related to dementia, hearing impairment, ear infection, middle ear diseases, uncorrected visual impairment, motion sickness, pre-existing conditions that preclude exercise, or having a heart pacemaker. Demographic information was collected for both the physical activity (mean age = 68.46, *n* = 6 females) and reading (mean age = 74.83, *n* = 12 females) intervention groups. See Tables 1, 2 below for further information regarding the participants included in the study as part of the experimental and control group, respectively.

**Table 1:** Demographic details regarding sex (males = 7), age (mean = 68.46, s.e. = 1.34), education (1 individual with a high school degree or equivalent; 8 with at least some post-secondary education including post-secondary certificate, diploma, or degree; 4 with postgraduate degrees), and ethnicity (all Caucasian, but one).

ID	Sex	Age	Education	Ethnicity	MoCA	PASE - B	PASE - PI
1	M	61	Post-Secondary	Caucasian	27	153.27	133.75
2	F	71	Post-Secondary	Caucasian	27	50	113.6
3	F	64	Post-graduate degree	Mixed	22	153.64	142.4
5	M	77	Post-graduate degree	Caucasian	29	97.74	60.61
6	F	60	Post-secondary	Caucasian	28	124.85	166.56
7	M	67	Post-secondary	Caucasian	29	176	143.2
8	F	67	Some post-secondary	Caucasian	28	52.31	83.2
9	M	70	Post-graduate degree	Caucasian	25	164.4	233.42
10	F	70	High school diploma	Caucasian	28	172.86	174.82
11	M	69	Post-secondary	Caucasian	30	219.67	176.3
12	M	75	Post-graduate degree	Caucasian	21	85.25	39.5
14	F	69	Post secondary	Caucasian	28	60.61	67.31
15	M	70	Some post-secondary	Caucasian	25	58.6	32.53

Cognitive function of each participant was assessed using the Montreal Cognitive Assessment (MoCA) and the scores of each participant have been reported above (mean = 26.69, s.e = 0.75). Participants also reported on their habitual exercise habits using the Physical Activity Scale for the Elderly (PASE) both at baseline (B; mean = 120.71, s.e. = 15.72) and at post-intervention (PI; mean = 120.55, s.e. = 16.84); the values self-reported by each participant are presented in the table. A higher PASE score indicates higher level of physical activity.

**Table 2:** Demographic details regarding sex (males = 2), age (mean = 74.83, s.e. = 1.48), education (4 individuals with high school degrees or equivalent; 7 with at least some post-secondary certificate, diploma, or degree; 3 with postgraduate degrees), and ethnicity (all Caucasian).

ID	Sex	Age	Education	Ethnicity	MoCA	PASE - B	PASE - PI
17	F	69	Post-Secondary	Caucasian	29	131.8	111.8
18	F	76	High school diploma	Caucasian	27	131.8	106.57
19	F	80	Post-secondary	Caucasian	23	165.86	143.71
21	M	74	Post-secondary	Caucasian	24	255.81	163.7
22	F	89	Post-secondary	Caucasian	26	76.4	51.4
23	F	86	Post-graduate degree	Caucasian	28	93.6	116.89
24	F	77	High school diploma	Caucasian	24	89.91	78.11
26	F	75	High school diploma	Caucasian	29	123.2	142.23
28	F	79	Post-secondary	Caucasian	28	99.54	91.53
29	F	71	Post-graduate degree	Caucasian	30	95	130
30	M	71	Post-graduate degree	Caucasian	27	133.53	182.71
31	F	74	Post-secondary	Caucasian	20	-	148.37
33	F	77	High school diploma	Caucasian	23	163.58	140.88
34	F	75	Post-secondary	Caucasian	25	124.6	162.8

Cognitive function of each participant was assessed using the Montreal Cognitive Assessment (MoCA) and the scores of each participant have been reported above (mean = 25.93, s.e = 0.77). Participants also reported on their habitual exercise habits using the Physical Activity Scale for the Elderly (PASE) both at baseline (B; mean = 129.59, s.e. = 12.98) and at post-intervention (PI; mean = 126.59, s.e. = 9.67); the values self-reported by each participant are presented in the table. A higher PASE score indicates higher level of physical activity.

### Procedure

Participants were asked to play Seas The Day three times a week for 6 weeks. Seas The Day was the only game that participants had access to in the provided VR headset. Participants were encouraged to maintain consistency in engaging with the VR intervention by playing in the mornings and preferably on the same days every week. They were notified that each intervention session would take approximately 15–20 min to complete. Participants were introduced to the OMNI rate of perceived exertion scale and were encouraged to achieve a light to moderate intensity (as indicated by the scale; ≤6 out of 10) when playing the game. Set-up of the VR intervention and ongoing participation in the intervention were supported in a number of ways. First, participants were provided with step-by-step software and hardware manuals (see Mehrabi et al., 2022). Second, each participant met with study staff or trainees for a remote introductory session *via* a video conference platform. The team member showed participants how to use the system while sharing their screen, so participants could see and become familiar with the visual information and the overall interaction with the system. The team member also demonstrated how to calibrate the system and played the game, stage by stage, while answering any questions as they arose. Participants were then encouraged to interact with the system during a familiarization session where they tried engaging with the intervention in the presence of a team member. During the familiarization session, participants were encouraged to speak aloud about what they were seeing and experiencing so they could be guided by the team member if they faced any difficulties. In addition to the familiarization session, participants were able to contact study staff and trainees to troubleshoot the system *via* email, phone, text or video calls at any time, as most appropriate for the situation and the participant’s comfort. During the troubleshooting video calls and to facilitate the explanation, participants were offered screen-sharing options as well as the option to see the view from the frontal camera of the study staff or trainee’s computer to see how the team member was located and moving in the physical space. Finally, study staff and/or trainees interacted with participants on a bi-weekly basis to ensure that participants were playing the game and engaging with the cognitive and perceptual tasks appropriately.

This study specifically used data from four perceptual tasks (audiovisual RT task, SIFI, SJ task, and TOJ task) to investigate the effects of engagement in physical activity in a virtual environment in community-dwelling older adults to determine how chronic (6 weeks) and acute bouts of physical activity within virtual environments, as compared to reading, impact multisensory integration processes. Participants completed various assessments, questionnaires, and cognitive tasks at baseline, before, and after selected intervention sessions. The pre-assessments took between 1 h and 40 min to 2 h and 10 min to complete. In addition, the researchers maintained bi-weekly meetings with the participants to monitor their progress and address any concerns. The post-assessments took approximately 1 h and 20 min to 1 h and 50 min to complete. Due to the fact that the materials presented in this manuscript stem from a larger pilot-study, time for testing the effects of engagement with a VR game on multisensory processes was limited and therefore not all tasks could be performed every week. Thus, the tasks with the most evidence for changes post-engagement in physical activity were selected to be performed more regularly as compared to tasks with limited evidence (see Merriman et al., 2015; O’Brien et al., 2017 for effects of physical activity on the SIFI task and see Mahoney et al., 2015; Basharat and Barnett-Cowan, 2023 for effects of physical activity on RT).

Once all the assessments and tasks were completed in week 1, participants began either the VR or reading intervention remotely from their homes. Each participant in the VR intervention group received an Oculus Quest 2 VR headset with Seas the Day installed, VR controllers, an instruction booklet, a weekly checklist for progress tracking, a VR system care guide, various questionnaires (see Table 3; Mehrabi et al., 2022 for further information) and blank sheets of paper for noting comments and concerns. The headset, sanitizing protocol, instruction booklet, and questionnaires were all delivered to participants’ homes *via* mail or by a research team member, adhering to public health guidelines for social and physical distancing during the pandemic. Participants in the reading group received the same items as the VR group, except for the Oculus Quest 2 VR headset, VR controllers, and the VR-related questionnaires (RPE, perceived enjoyment, and game user questionnaires [Self-reported physical (e.g., motion sickness, vertigo, nausea, etc.) or emotional (e.g., fear, anxiety, etc.) discomfort]). Those in the reading group read a physical book of their choice. Chronic effects of intervention were investigated *via* comparison of performance between the two groups on baseline and post-intervention sessions, while exploratory analyses were conducted to assess acute performance by comparing the weeks between baseline and post-intervention.

**Table 3:** Summary of data collected for the larger pilot non-randomized controlled trial (adapted from Mehrabi et al., 2022).

	Time Points														
		Baseline	Week 1		Week 2		Week 3		Week 4		Week 5		Week 6		Post-intervention
			Pre	Post	Pre	Post	Pre	Post	Pre	Post	Pre	Post	Pre	Post	
Background and demographic	Demographic questionnaire	x													
	MoCA	x													
	GDS-15	x													
	GAQ	x													
Executive function	OTMT	x	x	x			x	x			x	x			x
	VF	x	x	x			x	x			x	x			x
	Modified flanker task	x	x	x			x	x			x	x			x
Multisensory integration	SIFI task	x			x	x			x	x			x	x	x
	SJ task	x													x
	TOJ task	x													x
	RT task	x	x	x			x	x			x	x			x
Physical activity, mood, and exercise self-efficacy	PAAS	x	x	x	x	x	x	x	x	x	x	x	x	x	x
	PASE	x													x
	Exercise self-efficacy	x				x				x				x	
	PRE			x		x		x		x		x		x	
	Perceived enjoyment			x		x		x		x		x		x	
	Usability and game user experience questionnaire														x

Alongside demographic information, only the Response Time (RT), Sound Induced Flash Illusion (SIFI), Simultaneity Judgment (SJ), and Temporal Order Judgment (TOJ) tasks were analyzed for this manuscript and presented below to assess the effects of engaging in physical activity in VR and reading on multisensory processing. RPE (OMNI Rate of Perceived Exertion), perceived enjoyment, and the usability and game user experience questionnaire were completed only by those in the VR intervention group, as they pertain to their experience with Seas the Day. MoCA, Montreal Cognitive Assessment; GDS-15, Geriatric Depression Scale; GAQ, Get Active Questionnaire; OTMT, Oral Trail Making Test Part A and B; VF, Verbal Fluency test; PAAS, Physical Activity Affect Scale; PASE, Physical Activity Scale for the Elderly.

### Experimental setup

Participants were divided into an intervention group (physical activity in VR) and a control group (reading), recruited consecutively using the same inclusion and exclusion criteria (see Participants section). The intervention was 6-weeks long, with pre- and post-assessments as well as semi-structured interviews post-intervention. Note that in this paper we present the quantitative results related to sensory processing and the qualitative results will be discussed elsewhere in a separate publication.

The perceptual tasks presented in this manuscript were created with PsychoPy builder, exported into PsychoJS (Javascript), and hosted on Pavlovia, allowing the experiments to run in a browser with a precision of under 3.5 ms (Bridges et al., 2020). Participants completed the perceptual tasks on their computing device of choice (laptop or desktop computer) using Firefox as their browser. They were provided with instructions embedded in each task and were asked to sit in a quiet room, adjust the brightness and sound on their device, and not use headphones to ensure that the auditory stimuli appeared to stem from the same location as the visual stimuli.

During the perceptual tasks, participants were asked to directly face their personal computing device and place it at an approximate distance of 57 cm. The visual stimuli were presented as white circles subtending 2° of visual angle, appearing approximately 8° below the fixation cross (visual angle = 1.5°) for approximately 16 ms. Auditory stimuli were presented as a beep (approximately 3,500 Hz, 16 ms, 68 dBA) through the device’s speakers or through external speakers placed beside the screen. Each trial began with a stimulus presented after a delay of 1,000–3,000 ms to reduce temporal predictability. Participants used a computer keyboard to input their responses for each trial. They completed the SIFI, SJ, TOJ, and RT tasks in a randomized order during the baseline and post-intervention sessions. Practice trials were conducted before each experimental task.

### Detailed procedure of the perceptual tasks

Auditory stimuli were presented at a suprathreshold level (3,500 Hz, 16 ms, 68 dBA). The visual stimuli were presented as a 0.4° white circle (49.3 cd/m^2^) against a black background (0.3 cd/m^2^), appearing 2° below the fixation cross for 17 ms. The fixation cross, designed to minimize involuntary eye movements, resembled a combination of a bullseye and crosshair (visual angle = approximately 1.5°). Participants were instructed to fixate on this cross throughout the experimental procedure, as in previous in-lab studies. The stimulus onset asynchronies (SOAs) used in this study were chosen to ensure that participants could complete each task in a short period of time without losing interest or abandoning the task. To maintain consistency across the four perceptual tasks, the same stimuli and stimulus duration were used.

### Sound induced flash illusion

The SIFI task consisted of three conditions (vision-only, auditory-only, and audiovisual). In the vision-only block, participants were shown two flashes and asked to indicate the number of flashes they saw. In the auditory-only block, participants were presented with two beeps and asked to indicate the number of beeps they heard. The following SOAs were used in these conditions: 70 ms, 150 ms, and 230 ms for both 2 beep and 2 flash conditions. There were 30 trials in each of the unimodal conditions, with each SOA presented 10 times. Participants were explicitly told to respond as accurately as possible instead of quickly. The unimodal visual condition trials were randomly interleaved with the multimodal audiovisual trials, and the auditory block was completed separately, as instructions and modality of interest differed between auditory and audiovisual conditions.

**Figure 2:**
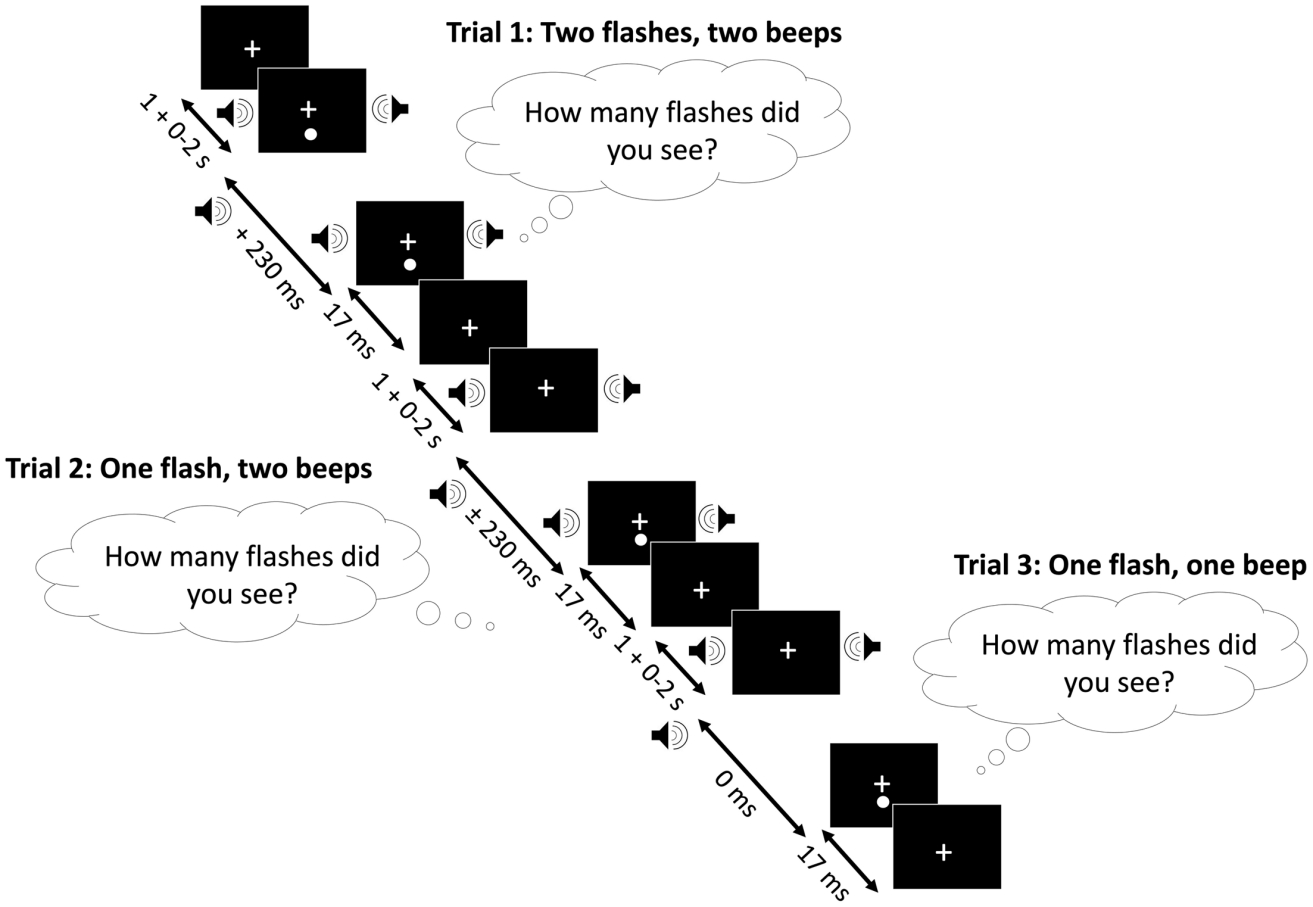
Sound induced flash illusion. The control conditions consited of the presentation of 2 flashes/2 beeps (trial 1) and 1 flash/1 beep (trial 3), while the illusory condition consisted of the presentation of 1 flash/2 beeps (trial 2). In the 1 flash/1 beep control condition, the auditory and visual stimuli were presented simultaneously. In the 2 flashes/2 beeps condition, the following SOAs were used: 70, 150, and 230 ms. In the illusory condition, the auditory stimulus was either presented prior to the presentation of the auditory and visual stimuli (auditory-lead) following a variable SOA of 70, 150, or 230 ms, or a visual stimulus was presented alongside the auditory stimulus followed by the second auditory stimulus (vision-lead) at a variable SOA of 70, 150, or 230 ms. For all the conditions, the first stimulus could appear 1–3 s after the fixation cross, and the second stimulus appeared between 0 and 230 ms after the first stimulus.

The audiovisual trials included two control conditions (1 beep/1 flash and 2 beeps/2 flashes) and an illusory condition (2 beeps/1 flash). In the audiovisual control conditions, the auditory and visual stimuli were presented simultaneously. In the 2 beeps/1 flash illusory condition, auditory-lead trials presented the auditory stimulus first, followed by simultaneous auditory and visual stimuli at variable SOAs. In vision-lead trials, the first auditory stimulus was accompanied by a visual stimulus, and the second auditory beep was presented after a variable SOA. The multimodal condition used the following SOAs: 0 ms, ±70 ms, ±150 ms, and ± 230 ms, with ‘+’ indicating vision-lead trials and ‘-’ indicating auditory lead trials. The three audiovisual conditions were randomly presented within the testing block to prevent response bias (see Figure 2 as well as Supplementary Figure S1; Supplementary Table S1 for further information).

Participants were asked to fixate on the fixation bullseye throughout the task and reported the number of flashes seen while ignoring the auditory stimuli. All conditions were repeated 10 times, totaling 100 trials (including 10 repetitions for 0 SOA with simultaneous presentation of a single beep and flash). In total, 166 trials were presented for all three conditions (vision-only, auditory-only, and audiovisual), including 6 practice trials to familiarize participants with the task. The task took approximately 10 min to complete. Previous literature indicates that participants may report perceiving three or more stimuli; thus, responses were not limited to ‘1’ or ‘2’, as participants could have perceived more than the presented number of stimuli (audio or visual). Participants were explicitly instructed to prioritize accuracy over speed.

Participants completed this task not only at the beginning and end of the intervention, but also six times during the intervention (pre- and post-gameplay or reading engagement during weeks 2, 4, and 6), for a total of 8 times.

Literature reveals that In this task, when a single flash is accompanied by two beeps in close temporal proximity, it can lead to the perception of two flashes (Shams et al., 2000, 2002). It has been found that healthy younger adults generally perceive the illusion when the SOA between the beeps and flash is less than or equal to 70–150 ms, whereas older adults are susceptible over a wider range of temporal SOAs. Here, susceptibility to the SIFI at longer SOAs (e.g., 230 ms) would indicate poorer temporal multisensory processing, as it would suggest that the central nervous system is unable to differentiate which cues belong together and which do not (Setti et al., 2011a,b, 2014).

### Simultaneity judgment

**Figure 3:**
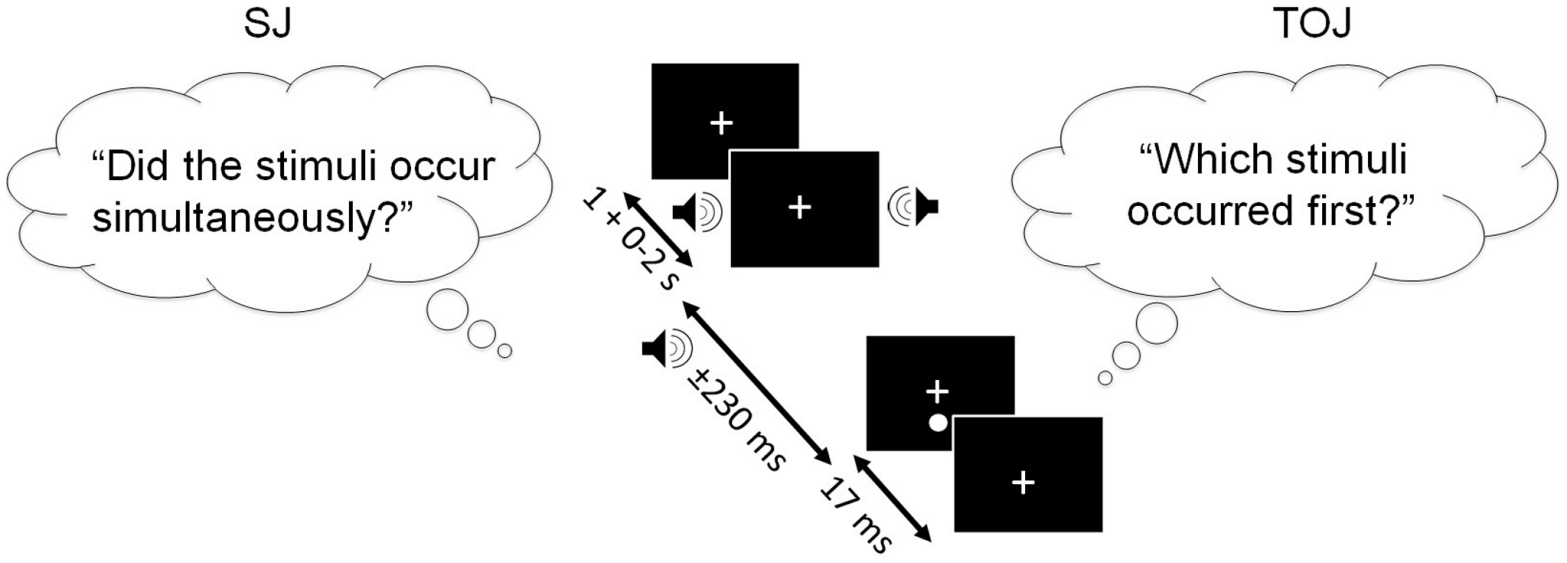
SJ task (left) and the TOJ task (right), presented with the SOAs of 0, ±70, ±150, ±230 ms (−ve = sound appeared before light). In both tasks, the first stimulus of the audiovisual pair appeared 1–3 s following the fixation cross, and the second stimulus appeared between 0 and 230 ms after the first stimulus. The figure depicts the auditory stimulus (i.e., beep) as appearing before the visual stimulus (i.e., flash). Note that the experimental design for the SJ and TOJ tasks is identical, but the instructions vary by task.

In the Simultaneity Judgment (SJ) task, participants were instructed to report whether they perceived the auditory and visual stimuli as occurring simultaneously (using the number ‘1’ key) or not (using the number ‘2’ key; see Figure 3). Participants were explicitly instructed to respond as accurately as possible, rather than responding quickly. The following SOAs were utilized: 0 ms, ±70 ms, ±150 ms, and ± 230 ms; here ‘+’ indicates vision-lead trials while ‘-’ indicates auditory lead trials. Ten trials were presented in a randomized order for each SOA, along with six practice trials, totaling 76 trials. This task took approximately 5–10 min to complete. Participants completed this task twice: before and after engagement in either intervention (physical activity or reading).

### Temporal order judgment

The Temporal Order Judgment (TOJ) task’s experimental design was identical to the SJ task, except for the task instructions. In this task, participants were asked to report whether they perceived the visual (using the number ‘1’ key) or auditory (using the number ‘2’ key) stimulus as appearing first. ‘Synchronous’ or ‘I do not know’ options were not provided for this task (see Figure 3). Participants were explicitly instructed to respond as accurately as possible, rather than responding quickly. This task took approximately 5–10 min to complete. Participants completed this task twice: before and after engagement in either intervention (physical activity or reading).

In both the SJ and TOJ tasks, participants were provided with the same pairs of audiovisual stimuli and they were either asked to determine if the stimuli occurred at the same or different times (SJ) or which stimulus appeared first (TOJ). These tasks have been found to be sensitive to both the temporal binding window (TBW), a window of time within which stimuli from different modalities are integrated and perceived as simultaneous, as well as the point of subjective simultaneity (PSS), the point at which participants are most likely to perceive stimuli as occurring simultaneously for the SJ task, and the point of maximal uncertainty for the TOJ task. Literature from the SJ and TOJ tasks indicates that there is an impairment in older adult’s ability to perceive the temporal order of events from multiple modalities due to a widening of the TBW (i.e., less precision) and a larger shift from true simultaneity (i.e., less accuracy; Poliakoff et al., 2006; Setti et al., 2011a,b; Chan et al., 2014a,b; Bedard and Barnett-Cowan, 2016; Basharat et al., 2018, 2019). A wider TBW has been associated with decreased speech comprehension (Maguinness et al., 2011; Setti et al., 2013), an inability to dissociate from distracting or inaccurate information (Wu et al., 2012), and an increase in susceptibility to falls (Setti et al., 2011a; Mahoney et al., 2014). Thus, a PSS closer to 0 and a narrower TBW would indicate optimal multisensory processing.

### Response time task

**Figure 4:**
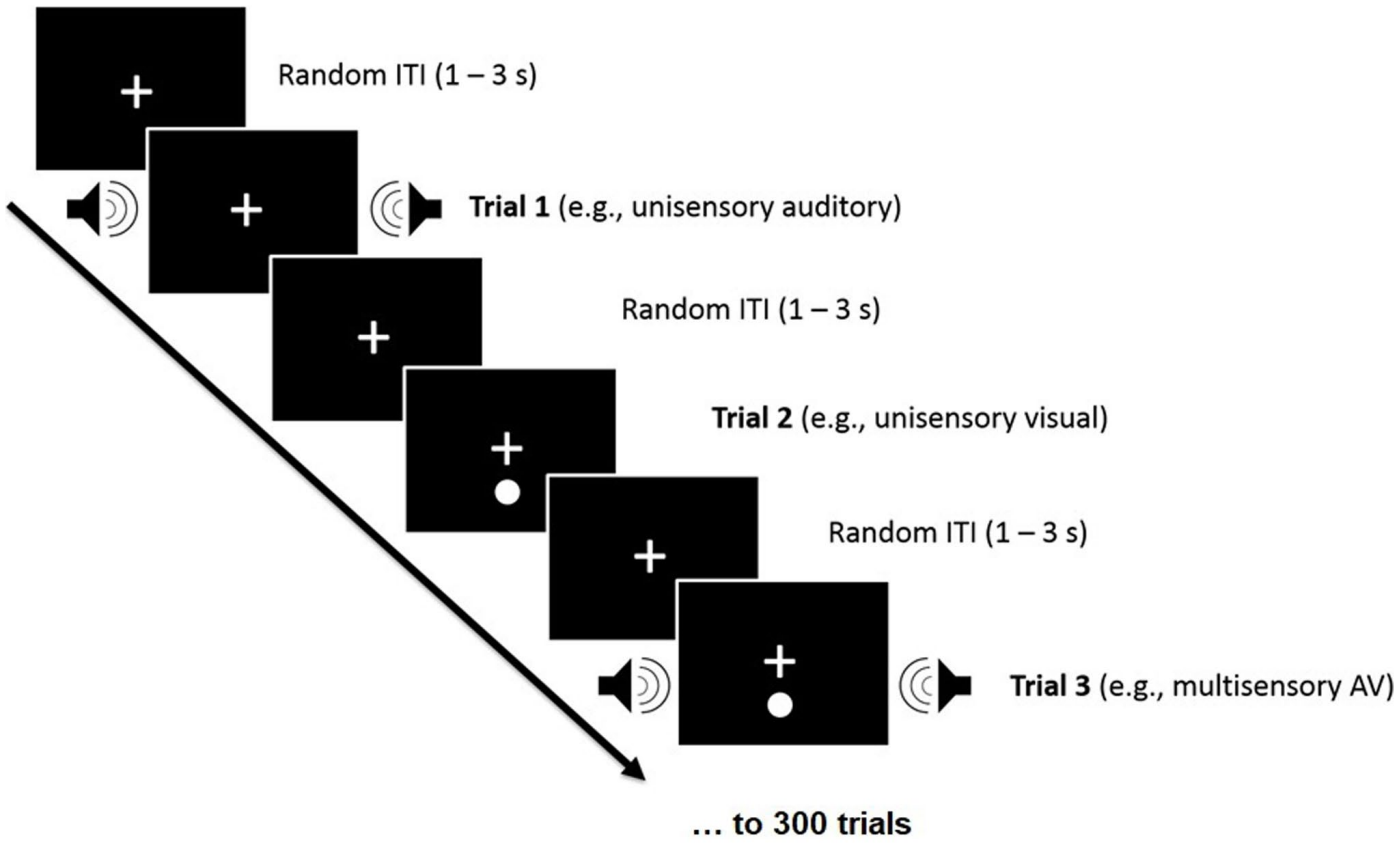
Participants were presented with unimodal [auditory (A) or visual (V)] or bimodal [audiovisual (AV)] stimuli and were asked to make speeded responses to all stimuli, regardless of sensory modality, by pressing the spacebar, which triggered the next trial. A, V, and AV stimuli were randomly presented with random inter-trial-intervals (ITIs) of 1–3 s.

For the Response Time (RT) task, participants were informed that they would either see a flash of light, hear a beep, or experience a combination of the two. Participants were instructed to press the response button (spacebar key) as soon as they detected any of the three experimental conditions: unisensory Visual (V), unisensory Auditory (A), or multisensory Audiovisual (AV) (audio and visual stimuli were presented simultaneously for each trial; see Figure 4). In this task, each stimulus was presented 50 times in random order, along with 6 practice trials. However, if a participant responded too quickly (<100 ms) or took longer than 3 s to respond to a trial where stimuli were presented, that trial was repeated. This task took approximately 5–10 min to complete. Participants completed this task not only at the beginning and end of the intervention period but also six times during the intervention (pre- and post-gameplay or reading during weeks 1, 3, and 5, for a total of 8 times).

Research indicates that multisensory stimuli are detected faster than unimodal stimuli and therefore may confer enhancement in activities of daily living (Laurienti et al., 2006; Peiffer et al., 2007; Diederich et al., 2008; Mahoney et al., 2011; Couth et al., 2018). Thus, a faster response time would indicate optimal integration.

## Statistical analysis

### Physical activity in VR and reading group comparison

Independent *t*-tests were used to compare the intervention and control group to assess differences between age, MoCA scores, and PASE scores at baseline and post-intervention.

### Sound induced flash illusion

Repeated measures ANOVAs were conducted to determine whether there were sensory differences between participants in the physical activity intervention and those in the reading control. Analyses were conducted separately on the proportion correct for unimodal and multimodal conditions (Merriman et al., 2015; O’Brien et al., 2017; Chan et al., 2018), as well as on acute and long-term data. To investigate the effects of long-term exposure to unimodal and multisensory perception between community-dwelling older adults who participated in the physical activity intervention and those in the reading control, a 2 (group: experimental or control) × 2 (time: baseline and post-intervention) mixed-design ANOVA was conducted for both auditory and visual cues. In order to assess whether participation in the physical activity intervention, compared to a reading control, would reduce susceptibility to the SIFI (hypothesis 1), a 2 (group) × 2 (time) × 4 (accuracy per condition: overall, 1-flash/1-beep, 2-flash/2-beep, or 1-flash/2-beeps) mixed-design ANOVA was conducted for the multisensory cues. Exploratory analyses were further conducted to examine potential acute changes in unimodal and multisensory perception, with a 2 (group) × 6 (time: pre- and post-week 2, pre- and post-week 4, pre- and post-week 6) mixed-design ANOVA conducted for both auditory and visual cues, and a 2 (group) × 6 (time) × 4 (accuracy per condition: overall, 1-flash/1-beep, 2-flash/2-beep, or 1-flash/2-beeps) mixed-design ANOVA conducted for the multisensory cues. Mauchly’s test of sphericity was conducted, and Greenhouse–Geisser adjustments were used to correct for lack of homogeneity of variance for all analyses if needed. Pairwise comparisons were also made to further assess the differences between group, condition, and time.

To further investigate the data, difference scores were calculated by subtracting baseline accuracy from post-intervention accuracy to assess long-term changes and by subtracting pre-session accuracy from post-session accuracy for sessions 1, 2, and 3 to assess acute changes. The data were analyzed using mixed-design ANOVAs. A 2 (group) × 1 (time: baseline - post-intervention) × 4 (condition) mixed-design ANOVA was conducted to investigate the effects of chronic effects of engaging in physical activity versus reading, and a 2 (group) × 3 (time: post-session 1 - pre-session 1, post-session 2 - pre-session 2, post-session 3 - pre-session 3) × 4 (condition) mixed-design ANOVA was conducted to investigate acute changes on the multisensory trials. A 2 (group) × 3 (time) mixed-design ANOVA was conducted for the unisensory conditions to investigate acute changes. Mauchly’s test of sphericity was performed, and Greenhouse–Geisser adjustments were used to correct for lack of homogeneity of variance for all analyses, if necessary. Pairwise comparisons were also conducted to further assess the differences between group, condition, and time. Additionally, independent t-tests were computed to investigate long-term changes for the unisensory conditions.

### Simultaneity and temporal order judgment tasks

To estimate the accuracy (PSS values) and precision (TBW) with which participants made their judgments for SJ and TOJ tasks, psychometric functions were fitted to each participant’s responses as a function of SOA using SigmaPlot version 12.5. Each task was analyzed individually for each participant, with participant data fit to both Gaussian (for the SJ task; Eq. 1) and logistic (for the TOJ task; Eq. 2) functions:


(1) $$y=a\;\cdot \;e\left(-0.5{\left(\frac{x-x0}{b}\right)}^{2}\right)$$

Where a is the amplitude, x0 is the PSS and b is the standard deviation.


(2) $$y=100\left(1+e\left(-\frac{x-x0}{b}\right)\right)$$

Where a is fixed to 1, x0 is the PSS and b is the standard deviation.

The best-fit parameters corresponding to the PSS and TBW were identified for each participant separately, and participants whose data were poorly estimated were excluded from further statistical analyses (*r*^2^ < 0.2; *n* = 1 in the physical activity group, *n* = 3 in the reading group).

As we were interested in the relationships between TBWs obtained from the two tasks and not their absolute size, we chose to analyze the b values (i.e., standard deviation) of these psychometric functions as a proxy for the size of the TBW to avoid discrepancies in the literature that differ when defining the absolute size of the TBW.

To assess whether participation in the physical activity intervention, as compared to the reading control, would reduce the width of the TBW (hypothesis 2), a 2 (group: engaging in physical activity or reading) × 2 (task: SJ or TOJ) × 2 (time: baseline and post-intervention) mixed-design ANOVA was conducted for the TBW to determine the impact of task, time, and participation in the intervention (or lack thereof). The same analysis was conducted with PSS values. For both the SJ and TOJ tasks, difference scores were also calculated by subtracting baseline values from post-intervention values for the TBW and PSS, and exploratory 2 (group) × 2 (task) mixed-design ANOVAs were conducted with said difference scores to further investigate and understand the data. Additionally, difference scores were computed for the ‘a’ values and an exploratory independent t-test was conducted with said values. Mauchly’s test of sphericity was conducted, and if the dependent variables were not proportional to the identity matrix, the Greenhouse–Geisser adjustment was used for the mixed-design ANOVA. The Shapiro–Wilk test was used to determine normality for the independent *t*-tests. Pairwise comparisons were also made to assess differences between the tasks, intervention, and group for the mixed-design ANOVA.

### Response time task

#### Error analysis and outlier removal

As previously mentioned, participants responded to 150 trials in total (50 per condition). Data trimming procedures were not applied (see Gondan, 2010; Gondan and Minakata, 2016; Mahoney and Verghese, 2018, 2019; Basharat et al., 2019; Mahoney et al., 2019); however, responses faster than 100 ms and slower than 1,500 ms were set to infinity rather than excluded (see Mahoney and Verghese, 2019 for a race model inequality (RMI) tutorial and (Basharat et al., 2019) where this method of data trimming was recently used). Here, we found that <1% of trials for both engagement in physical activity (average accuracy = 99.78%) and reading (average accuracy = 99.4%) groups were outliers that were set to infinity.

#### Mean response time analysis

In order to assess whether participation in the physical activity intervention would reduce response time more than participation in the reading control (hypothesis 3), a 2 (group) × 2 (time: baseline and post-intervention) × 3 (modality: auditory, visual, or audiovisual) mixed-design ANOVA was conducted to determine the long-term impact of time, modality, and participation in the physical activity versus reading interventions. Additionally, an exploratory mixed-design 2 (group) × 6 (time: pre-, post-week 1; pre-, post-week 3; pre-, post-week 5) × 3 (modality) ANOVA was conducted to determine the acute impact of time, modality, and participation in the physical activity versus reading interventions. To further investigate the data, difference scores were calculated by subtracting baseline response time from post-intervention response time to assess long-term changes, and by subtracting pre-session response time from post-session response time for sessions 1, 2, and 3 to assess acute changes, which were compared using exploratory mixed-design ANOVAs. A 2 (group) × 1 (time: baseline - post-intervention) × 3 (modality) mixed-design ANOVA was conducted to assess long-term effects of intervention on multisensory processing. A 2 (group) × 3 (time: post-session 1 - pre-session 1, post-session 2 - pre-session 2, post-session 3 - pre-session 3) × 3 (modality) mixed-design ANOVA was conducted to assess acute effects of intervention on multisensory processing. Mauchly’s test of sphericity was conducted, and Greenhouse–Geisser corrections were applied if necessary. Pairwise comparisons were utilized to further assess the differences between time, modality, and experimental group. The same analyses as those conducted with mean RT data were conducted for the median RT data; these results can be found in the Supplementary material.

#### Test of the race model

The race model asserts that the response to redundant signals is produced by the modality that processes its respective signal the fastest and thus is the “winner” of the race (Raab, 1962). Race model violations are typically tested using cumulative distribution function (CDF) models, which compare the observed CDF distribution to the predicted CDF distribution (Miller, 1982).

To compute CDFs, each participant’s data was sorted in ascending order for all three conditions (A, V, AV). Each participant’s RTs were then quantized into 5th percentile bins until the 100th percentile was reached, yielding a total of 21 bins.

Observed CDF distributions were formed using the following equation (Eq. 3):

CDF_observed_ = P (RT_AV_ ≤ *t*)(3)

Where RT_AV_ represents the RT observed for the multisensory condition for any latency, *t* (Colonius and Diederich, 2006; Mahoney et al., 2011).

Predicted CDF models were formed using the following equation (Eq. 4):

CDF_predicted_ = Min [P (RT_A_ ≤ *t*) + P (RT_V_ ≤ *t*), 1] (4)

Where RT_A_ and RT_V_ represent the RTs observed for unisensory condition ‘A’ (i.e., auditory) and ‘V’ (i.e., vision), for any time, *t* (Colonius and Diederich, 2006; Mahoney et al., 2011).

Differences between the observed CDF distribution and the predicted CDF distribution were calculated for every participant across all percentile bins as follows (Eq. 5):

${\mathrm{RT}}_{\mathrm{AV}}=P\;\left({\mathrm{RT}}_{\mathrm{AV}}\leq t\right)-\min\left[P\;\left({\mathrm{RT}}_{A}\leq t\right)+P\;\left({\mathrm{RT}}_{V}\leq t\right),1\right]$ (5)

When the observed CDF is less than or equal to the predicted CDF, the race model is accepted. However, the race model is violated when the observed CDF is greater than the predicted CDF. Thus, a negative value (or zero) indicates acceptance of the race model, while values greater than zero provide evidence for multisensory integration as they are indicative of race model violations (Colonius and Diederich, 2006; Mahoney et al., 2011, 2014).

To investigate if the race model inequality was violated, Gondan’s permutations were computed over the fastest quartile (0–25%) of responses (Gondan, 2010; Gondan and Minakata, 2016; Mahoney and Verghese, 2019) for all sessions for both those who engaged in physical activity and reading (see Tables 4, 5 below for outcomes of Gondan’s permutations for the experimental and control groups). Further, in addition to performing Gondan’s permutation test of the race model (Gondan and Minakata, 2016), we also calculated the area under the curve (AUC), which served as our independent variable, to further quantify the magnitude of RMI violation over the first quartile of responses. As described in (Mahoney and Verghese, 2019), the AUC was calculated for each time bin over the 0-25th percentile, where the difference value obtained from the observed CDF and the predicted CDF from the first time bin (i.e., 0%) was summed with the difference value obtained from the second time bin (5%) and divided by two. This process was repeated for the subsequent time bins until the 25th percentile was reached. All the values obtained were summed to generate a total AUC of the CDF difference wave during the 25th percentile.

**Table 4:** Outcome of Gondan’s permutation for 8 of the sessions where data was collected for those who engaged in physical activity; the statistically significant outcome of Gondan’s permutations indicate that race model inequality was violated for all the sessions.

Session	tmax	tcrit	value of p
Baseline	4.503	2.281	≤0.001
1 Pre-physical activity engagement	3.064	2.337	≤0.05
1 Post-physical activity engagement	3.605	2.260	≤0.05
2 Pre-physical activity engagement	5.807	2.208	≤0.001
2 Post-physical activity engagement	4.965	2.095	≤0.001
3 Pre-physical activity engagement	5.866	2.336	≤0.001
3 Post-physical activity engagement	4.879	2.205	≤0.001
Post-physical activity engagement	6.185	2.164	≤0.001

**Table 5:** Outcome of Gondan’s permutation for 8 of the sessions where data was collected for the reading group; the statistically significant outcome of Gondan’s permutations indicate that race model inequality was violated for all the sessions.

Session	tmax	tcrit	value of *p*
Baseline	5.991	2.27	≤ 0.01
1 Pre-physical activity	7.207	2.26	≤ 0.01
1 Post-physical activity	9.201	2.146	≤ 0.001
2 Pre-physical activity	3.620	2.179	≤ 0.01
2 Post-physical activity	4.773	2.094	≤ 0.001
3 Pre-physical activity	5.909	2.153	≤ 0.0001
3 Post-physical activity	6.394	2.339	≤ 0.0001
Post-physical activity	7.094	2.291	≤ 0.001

In order to assess whether participation in the physical activity engagement intervention would increase race model violations more so than participation in the reading control (hypothesis 3), a mixed-design 2 (group: engagement in physical activity or reading) × 2 (time: baseline and post-intervention) ANOVA was conducted with AUC values to compare the long-term effects of engagement in physical activity and reading interventions on the AUC. Additionally, an exploratory mixed-design 2 (group: engagement in physical activity or reading) × 6 (time: pre-, post-week 1; pre-, post-week 3; pre-, post-week 5) ANOVA was conducted with AUC values to compare the acute effects of engagement in physical activity and reading interventions on the AUC.

To further investigate the data, difference scores were computed for the AUC by subtracting baseline AUC from post-intervention AUC to assess long-term changes and by subtracting the pre-session AUC from post-session AUC for sessions 1, 2, and 3 to assess acute changes. These were compared using mixed-design ANOVAs. Exploratory independent t-tests were computed to compare the difference score obtained from post-intervention and baseline sessions between participants who engaged in physical activity versus reading interventions. Moreover, an exploratory 2 (group) × 3 (time: post-session 1 - pre-session 1, post-session 2 - pre-session 2, post-session 3 - pre-session 3) mixed-design ANOVA was conducted to assess acute effects of intervention on multisensory processing. Mauchly’s test of sphericity was conducted, and Greenhouse–Geisser corrections were applied if necessary. Pairwise comparisons were also made to assess the differences between time and experimental group.

## Results

The results revealed that the reading group (mean age = 74.83, s.e. = 1.48) was significantly older compared to those who engaged in physical activity (*p* < 0.001; mean age = 68.46, s.e. = 1.34) and there were significantly more females in the reading as compared to those who engaged in physical activity. No further differences were found.

### SIFI: audiovisual conditions

**Figure 5:**
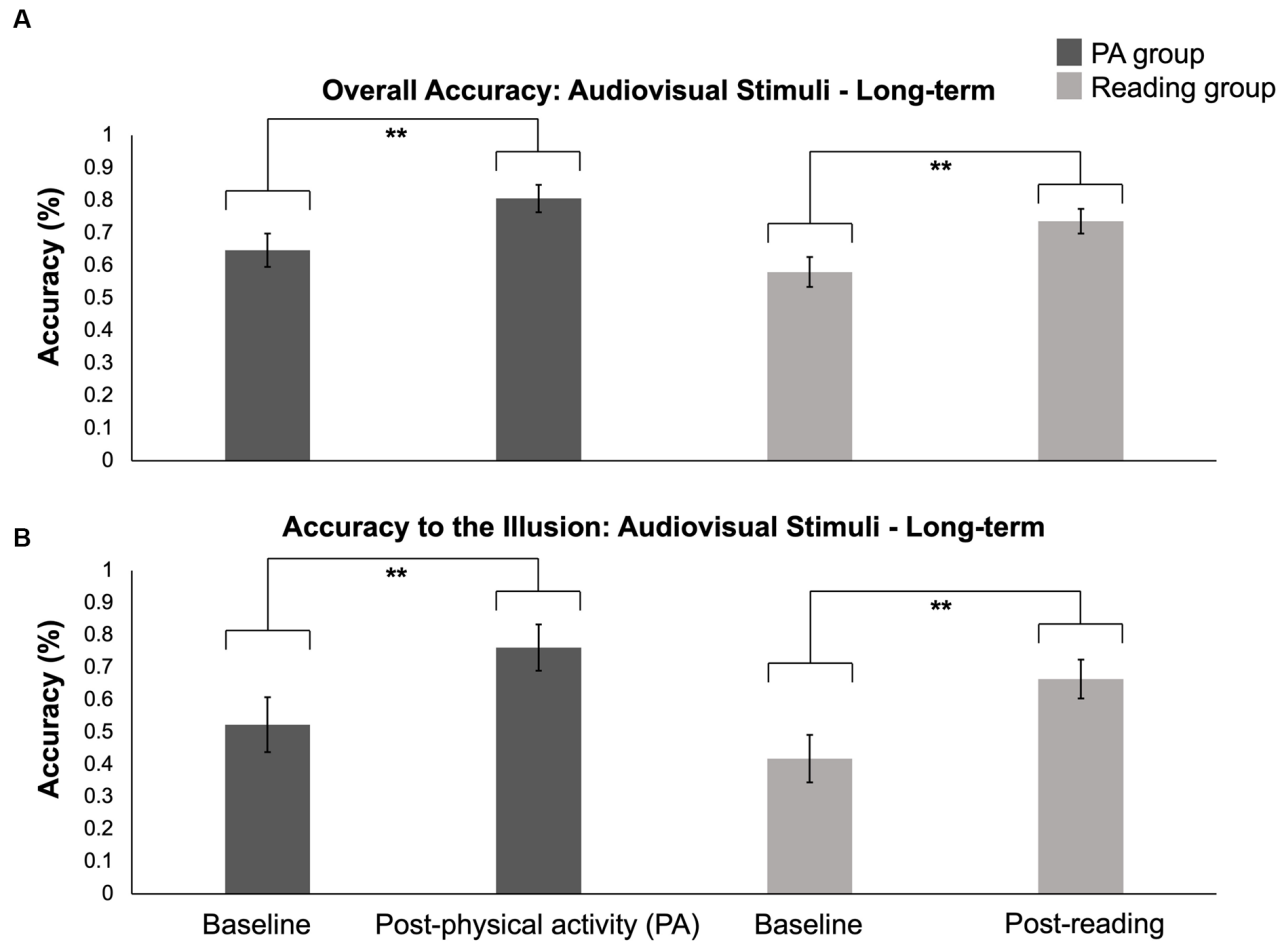
Accuracy for overall and illusory conditions during baseline and post-intervention sessions in the physical activity (PA) and reading interventions. **(A)** Overall accuracy at baseline and post-intervention. **(B)** Illusory condition accuracy at baseline and post-intervention. Error bars represent SEM. A main effect of time was found (*p* = 0.010), but pairwise comparison between baseline and post-intervention was not significant (*p* = 0.254).

A 2 (group) × 2 (time) × 4 (conditions) mixed-design ANOVA was conducted to investigate the effects of long-term exposure to physical activity and reading on the SIFI. The analysis revealed a significant interaction between time and condition (*F* (3, 69) = 9.004, *p* < 0.001; *η*^2^_p_ = 0.281). Planned pairwise comparisons showed that compared to accuracy on the illusory trials at baseline, the accuracy was higher for all conditions at both baseline and post-intervention, including the accuracy in the illusory condition at the time of post-intervention (*p* < 0.001). Additionally, the results indicated that compared to overall accuracy achieved at baseline, the accuracy was higher for all other conditions (i.e., 1-flash/1-beep, 2-flashes/2-beeps, 1-flash/2-beeps) at both baseline and post-intervention (*p* < 0.05), except for the accuracy achieved for the illusory condition from the post-intervention session (see Table 6 for more information). Note that Levene’s test for Equality of Variance was violated for time and condition; thus, non-parametric Friedman tests were conducted, revealing a main effect of time (*χ*2 (1) = 6.570, *p* = 0.010) and a main effect of condition (*χ*2 (3) = 70.024, *p* < 0.001).Conover’s post-hoc pairwise comparisons investigating the main effect of condition revealed that the main effect was driven by significantly higher accuracy for the 1-flash/1-beep condition compared to the illusory (*p* = 0.002) and overall accuracy conditions (*p* = 0.019). The pairwise comparison investigating the main effect of time failed to reveal a significant difference between accuracy obtained at baseline and post-intervention (*p* = 0.254), suggesting a lack of power to differentiate where the effect arose from. Finally, the analysis did not find a significant effect of group (*F* (1, 23) = 2.711, *p* = 0.113; *η*^2^_p_ = 0.105). See Figure 5 and Supplementary Figure S2 for long-term accuracy scores obtained from those who engaged in physical activity and reading interventions. The results used to assess hypothesis 1 are concluded; what follows are exploratory analyses that investigate potential acute changes, difference scores, and changes in unimodal perception.

**Table 6:** *Post-hoc* comparisons for the audiovisual condition of the SIFI during baseline and post-intervention sessions.

Time*Condition	Time*Condition	Mean Difference	SE	*t*	Cohen’s *d*	*p*bonf.
B, Overall	PI, Overall	−0.147	0.037	−3.966	−0.833	0.005
	B, Illusion	0.143	0.043	3.347	0.812	0.031
	B, 2 flash	−0.191	0.043	−4.467	−1.083	< 0.001
	PI, 2 flash	−0.203	0.047	−4.329	−1.154	< 0.001
	B, 1 flash	−0.285	0.043	−6.682	−1.620	< 0.001
	PI, 1 flash	−0.350	0.047	−7.454	−1.986	< 0.001
PI, Overall	B, Illusion	0.29	0.047	6.173	1.645	< 0.001
	PI, 1 flash	−0.203	0.043	−4.754	−1.153	< 0.001
B, Illusion	PI, Illusion	−0.228	0.037	−6.152	−1.293	< 0.001
	B, 2 flash	−0.334	0.043	−7.814	−1.895	< 0.001
	PI, 2 flash	−0.346	0.047	−7.375	−1.965	< 0.001
	B, 1 flash	−0.428	0.043	−10.029	−2.432	< 0.001
	PI, 1 flash	−0.493	0.047	−10.499	−2.798	< 0.001
PI, Illusion	B, 1 flash	−0.201	0.047	−4.276	−1.139	0.001
	PI, 1 flash	−0.265	0.043	−6.207	−1.505	< 0.001
B, 2 flash	PI, 1 flash	−0.159	0.047	−3.389	−0.903	0.027
PI, 2 flash	PI, 1 flash	−0.147	0.043	−3.433	−0.833	0.023

Results indicate higher accuracy for all conditions at both baseline and post-intervention compared to the illusory condition at baseline (*p* < 0.001). Higher accuracy was found for all conditions compared to overall accuracy at baseline (*p* < 0.05), except for the illusory condition during post-intervention. Only significant results are presented.

**Figure 6:**
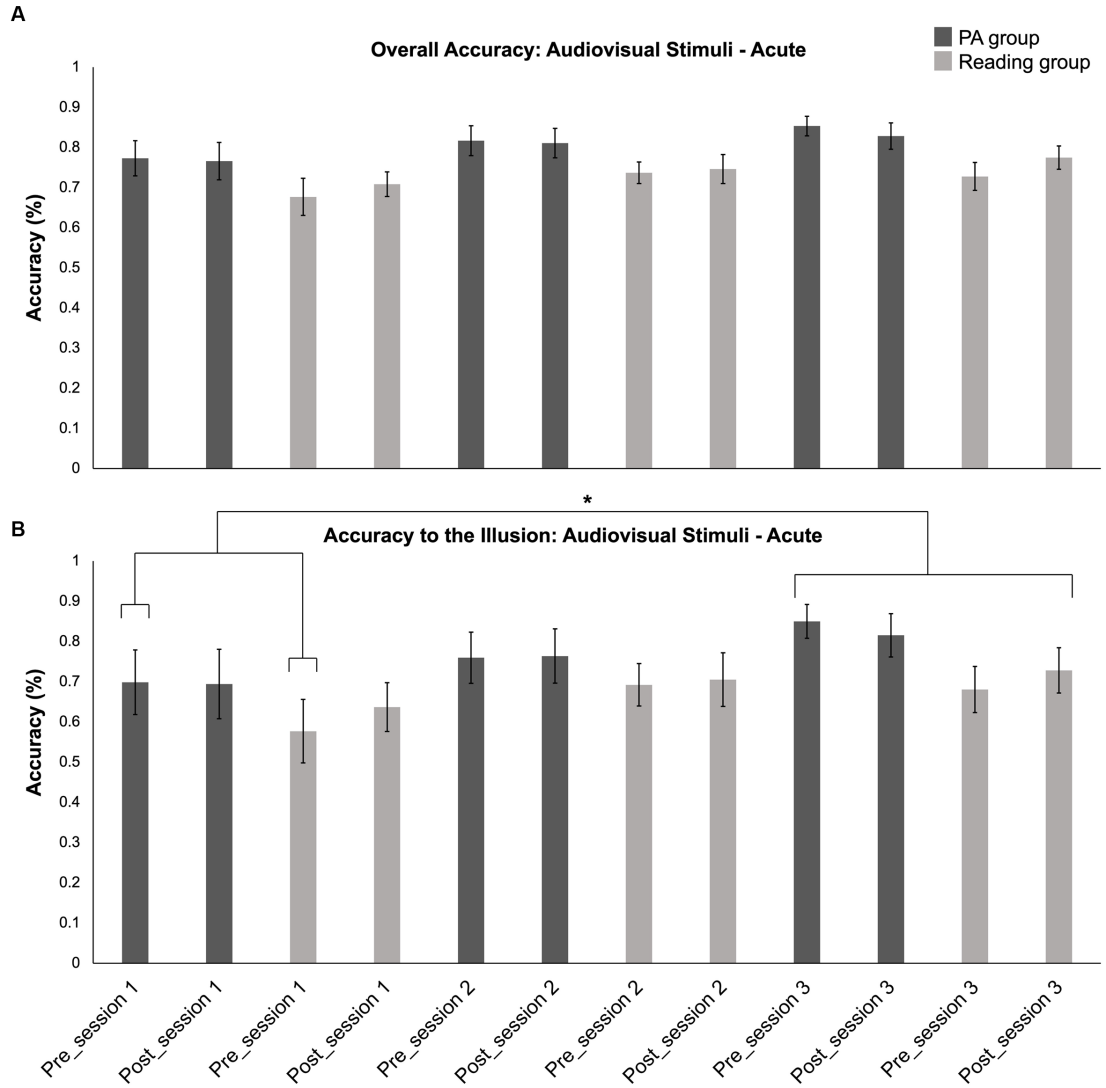
Acute accuracy for overall and illusory conditions during sessions 1, 2, and 3 in the physical activity (PA) and reading interventions. **(A)** Overall acute accuracy. **(B)** Illusory condition acute accuracy. Those who engaged in physical activity had significantly higher accuracy than the reading group (*p* = 0.037). Error bars represent SE.

A 2 (group) × 6 (time) × 4 (conditions) mixed-design ANOVA investigating the acute effects of engagement in physical activity and reading revealed a significant main effect of group (*F* (1, 18) = 5.051, *p* = 0.037; *η*^2^_p_ = 0.219). Pairwise comparisons found that participants in the physical activity intervention (mean accuracy = 85.6%) were significantly more accurate compared to those in the reading group (mean accuracy = 78.2%; *p* = 0.037). Additionally, a significant interaction between time and condition (*F* (15, 270) = 1.753, *p* = 0.041; *η*^2^_p_ = 0.089) was found. Pairwise comparisons investigating the interaction between time and condition revealed multiple significant outcomes (refer to Supplementary Table S2 for details). Of primary interest, the results showed that compared to pre-intervention accuracy for the illusion in session 1, accuracy was higher for both pre- (*p* = 0.001) and post-sessions (*p* = 0.047) of session 3. Moreover, the results demonstrated that participants achieved higher accuracy on the 1-flash/1-beep trials at all times that SIFI was administered as compared to overall accuracy (*p* < 0.01) and the accuracy achieved for the illusory condition (*p* < 0.05). Additionally, accuracy for the 1-flash/1-beep condition was also higher than the 2-flash/2-beep condition, primarily during sessions 2 and 3. Note that Levene’s test for Equality of Variance was violated for time and condition, so non-parametric Friedman tests were conducted, revealing a main effect of condition (*χ2* (3) = 138.972, *p* < 0.001), but no main effect of time (*χ2* (5) = 3.282, *p* = 0.657). Pairwise comparisons investigating the main effect of condition found that accuracy for the 1-flash/1-beep condition was significantly higher than all the other conditions, including accuracy for the overall condition (*p* = 0.003), illusory condition (*p* = 0.003), and 2-flashes/2-beeps condition (*p* = 0.014). See Figure 6 and Supplementary Figure S3 for acute accuracy scores obtained during the 6-week intervention from those who engaged in physical activity and reading. Given that there was a significant difference in age between the exercise and reading groups, we reran these analyses using age as a covariate. When age was added as a covariate, the analysis revealed no main effect of group (*F* (1, 17) = 0.4706, *p* = 0.412; *η*^2^_p_ = 0.040) and there were no other subsequent effects. What this indicates is that the effects reported above related to engagement in physical activity for the SIFI task may be due to the fact that the participants in the physical activity intervention were younger than those in the reading group and therefore less susceptible to the SIFI.

To further investigate the data, difference scores were computed by subtracting baseline accuracy from post-intervention accuracy to assess long-term changes and by subtracting pre-session accuracy from post-session accuracy for sessions 1, 2, and 3 to assess acute changes. A 2 (group) × 1 (time: post-intervention - baseline) × 4 (condition) mixed-design ANOVA investigating the effects of long-term exposure to physical activity and reading revealed a main effect of condition (*F* (3, 72) = 8.070, *p* < 0.001; *η*^2^_p_ = 0.252). Pairwise comparisons were conducted to investigate the main effect of condition, which revealed that the difference in accuracy for the illusory condition was significantly higher than that for the 2-flashes/2-beeps (*p* < 0.001) and 1-flash/1-beep conditions (*p* = 0.005). This indicates that susceptibility to the illusion not only decreased after 6 weeks of both engagement in physical activity and reading interventions, but also showed greater improvement compared to the control conditions. Further, the pairwise comparisons revealed that the difference in overall accuracy was significantly higher than that for the 2-flashes/2-beeps condition (*p* = 0.035). The ANOVA failed to find a main effect of group (*F* (1, 24) = 0.225, *p* = 0.639; *η*^2^_p_ = 0.009) or a significant interaction between condition and group (*F*(3, 72) = 0.223, *p* = 0.880; *η*^2^_p_ = 0.009). See Supplementary Figures S4, S5 for a comparison of difference scores obtained by subtracting baseline accuracy from post-intervention accuracy scores for the physical activity and reading interventions.

A 2 (group) × 3 (time: post-session 1 - pre-session 1, post-session 2 - pre-session 2, post-session 3 - pre-session 3) × 4 (condition) analysis was conducted to investigate the acute effects of time, condition, and intervention. The analysis failed to reveal significant effects for group (*F*(1, 20) = 1.606, *p* = 0.220; *η*^2^_p_ = 0.074), time (*F*(2, 40) = 0.433, *p* = 0.652; *η*^2^_p_ = 0.021), and condition (*F*(3, 60) = 0.017, *p* = 0.997; *η*^2^_p_ < 0.001). Additionally, no significant interactions were found for group and time (*p* = 0.837), group and condition (*p* = 0.818), or time, condition, and group (*p* = 0.996). See Supplementary Figures S6, S7 for a comparison of the acute difference scores between the physical activity and reading interventions.

See Supplementary Figures S8–S11 for unimodal (control) condition analysis for the SIFI. To summarize, we did not find any significant differences between the two groups. Of interest, accuracy for auditory cues during the post-intervention session was significantly higher than at baseline (*p* = 0.011). Additionally, when an independent t-test was conducted to examine the long-term effects of physical activity and reading on the visual condition, the results revealed a near-significant difference between the two groups (*t*(25) = −1.837, *p* = 0.078; *Cohen’s d* = − 0.707), with the reading group demonstrating a larger difference in accuracy compared to those who engaged in physical activity.

### Simultaneity and temporal order judgment tasks

Initially, a mixed-design ANOVA (2 × 2 × 2) was conducted for TBW, considering group (engagement in physical activity or reading), task (SJ or TOJ), and time (baseline and post-intervention). Due to a violation of Levene’s test for Equality of Variance, Friedman tests were performed, revealing a significant main effect of task (*χ2*(1) = 13.365, *p* < 0.001) but not of time (*χ2*(1) = 2.504, *p* = 0.114). Pairwise comparisons indicated wider TBWs for the SJ task (*p* = 0.021) and wider TBWs at baseline compared to post-intervention. No significant effect of group (*F* (1, 21) = 0.055, *p* = 0.816; *η*^2^_p_ = 0.003) or interaction between group, time, and task (*F* (1, 21) = 0.054, *p* = 0.818; *η*^2^_p_ = 0.003) was found. See Supplementary Figure S12 (average Gaussian [SJ] function) and Supplementary Figure S13 (average Logistic [TOJ] function).

The following exploratory analyses investigated long-term intervention effects on TBW, PSS and amplitude. Difference scores were used to assess the long-term effects of engagement in physical activity and reading on SJ and TOJ tasks. A mixed-design ANOVA (2 × 2) with difference scores for TBW and PSS did not reveal any significant main effects or interactions for either TBW or PSS. An independent t-test investigating amplitude differences between the physical activity and reading interventions did not reveal a significant difference. See Supplementary Figure S14 for a graphical representation of the difference scores obtained for the SJ and TOJ tasks for both groups, and Supplementary Figure S15 for the amplitude difference scores.

### Response time

**Figure 7:**
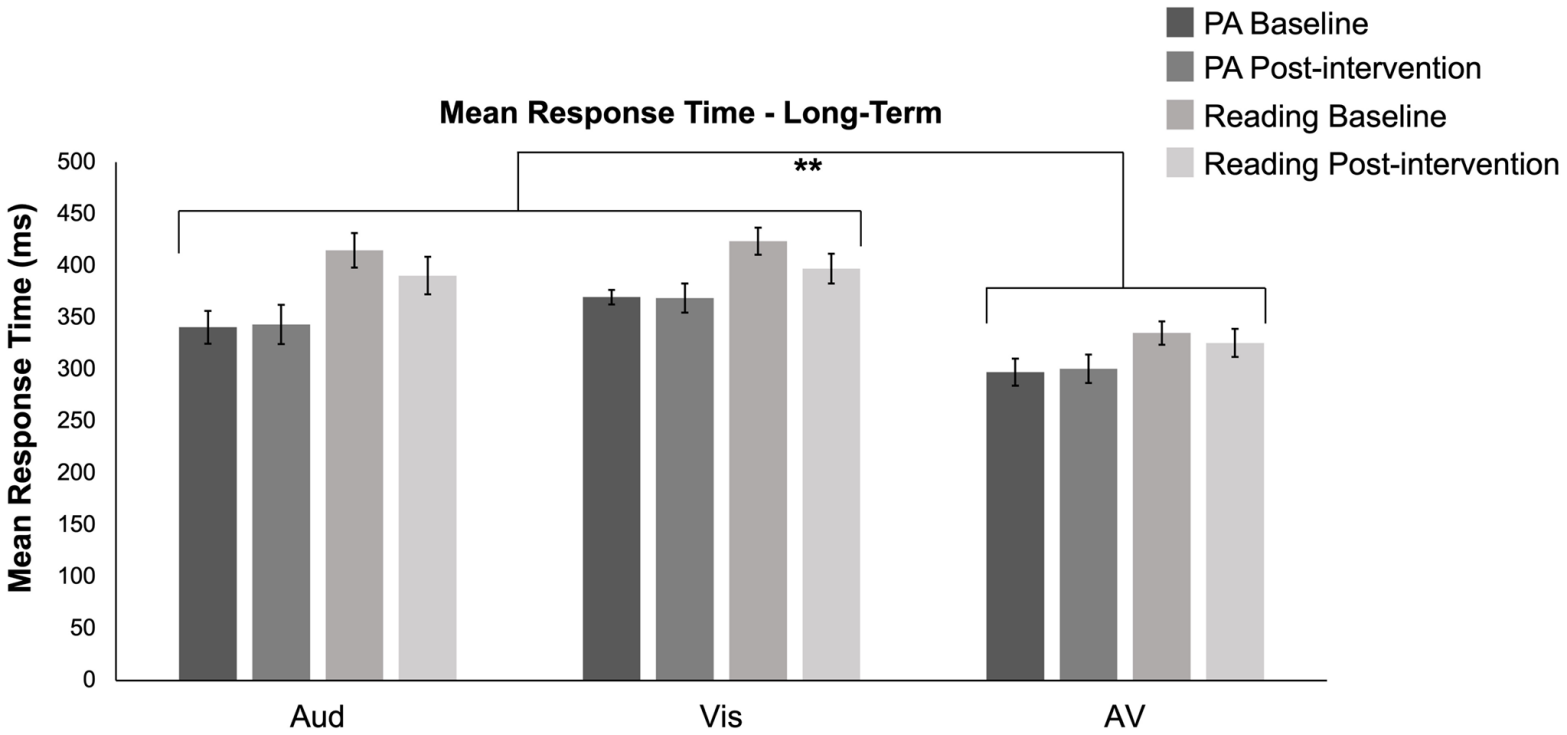
The mean response time for baseline (darker shade) and post-intervention (lighter shade) sessions in both the physical activity (PA; dark grey) and reading (light grey) groups across auditory, visual, and audiovisual trials. The reading group displayed longer response times (mean = 381.305, s.e. = 16.832) compared to those who engaged in physical activity (mean = 336.9172, s.e. = 12.954, *p* = 0.012). Moreover, response times for audiovisual stimuli (mean = 314.792, s.e. = 9.275) were significantly faster than auditory (mean = 372.524, s.e. = 18.195; *p* < 0.001) and visual (mean = 390.0177, s.e. = 13.047; *p* < 0.001) modalities. Aud, auditory stimuli; Vis, visual stimuli; AV, audiovisual stimuli; pre, baseline; post, post-intervention. Error bars indicate the SEM.

A 2 (group) × 2 (time: baseline and post-intervention) × 3 (modality: auditory, visual, or audiovisual) mixed-design ANOVA was conducted to determine the long-term effects of participation in the two interventions. The analysis revealed significant main effects of group (*F* (1, 24) = 7.318, *p* = 0.012; *η*^2^_p_ = 0.234) and modality (*F* (1.445, 34.673) = 67.898, *p* < 0.001; *η*^2^_p_ = 0.739). Pairwise comparisons showed longer response times for the reading group compared to those who engaged in physical activity (*p* = 0.012), and both auditory (*p* < 0.001) and visual (*p* < 0.001) stimuli had significantly longer response times than audiovisual stimuli. No significant main effect of time (*F* (1, 24) = 0.907, *p* = 0.350; *η*^2^_p_ = 0.036) or interaction between group, time, and modality (*F* (1.684, 40.417) = 0.593, *p* = 0.556; *η*^2^_p_ = 0.024) was found. Figure 7 presents mean response time data for baseline and post-intervention sessions for both groups. Hypothesis 3 analyses on mean response time are followed by exploratory analyses investigating potential acute changes and difference scores from longitudinal and acute sessions. Just as the main effect of group was investigated above for the SIFI due to a significant difference in age between the exercise and reading groups, we re-ran these analyses using age as a covariate. When age was added as a covariate, the analysis revealed no main effect of group (*F* (1, 23) = 0.707, *p* = 0.409; *η*^2^_p_ = 0.030). We did however find a significant interaction between group and time (*F* (1, 22) = 5.00, *p* = 0.035; *η*^2^_p_ = 0.179), a main effect of time (*F* (1, 23) = 4.360, *p* = 0.048; *η*^2^_p_ = 0.159), and a main effect of age (*F* (1, 23) = 5.140, *p* = 0.033; *η*^2^_p_ = 0.183). The lack of a main effect of group and a main effect of age indicate that the effects reported above related to those in the reading group as having longer response times may be due to the fact that the participants in the reading group were older than those who engaged in physical activity and therefore had slower response times.

A 2 (group: experimental or control) × 6 (time: pre-, post-week 1; pre-, post-week 3; pre-, post-week 5) × 3 (modality: auditory, visual, or audiovisual) mixed-design ANOVA was conducted to determine the acute impact of physical activity engagement versus reading. The analysis revealed a significant main effect of group (*F* (1, 23) = 9.127, *p* = 0.006; *η*^2^_p_ = 0.284), with longer response times for the reading group compared to those who engaged in physical activity (p = 0.006). Due to a violation of the Levene’s test for Equality of Variance, a Friedman test was conducted, revealing a significant main effect of modality (*χ2*(2) = 134.776, *p* < 0.001). Pairwise comparisons showed longer response times for both auditory (*p* < 0.001) and visual (*p* < 0.001) stimuli compared to audiovisual stimuli. No significant main effect of time (*χ2*(5) = 8.246, *p* = 0.143) or interaction between group, time, and modality (*F* (4.692, 107.914) = 1.052, *p* = 0.389; *η*^2^_p_ = 0.044) was found. See Supplementary Figure S16 for mean response time data for acute conditions. Just as the main effect of group was investigated above, here too we re-ran these analyses using age as a covariate. When age was added as a covariate, the analysis revealed no main effect of group (*F* (1, 22) = 0.759, *p* = 0.393; *η*^2^_p_ = 0.033). We did however find a significant main effect of age (*F* (1, 22) = 5.289, *p* = 0.031; *η*^2^_p_ = 0.194). Similar to the results presented above, this lack of a main effect of group and the significant main effect of age indicate that the effects reported above of those in the reading group having longer response time may be explained by the age difference between the two groups.

**Figure 8:**
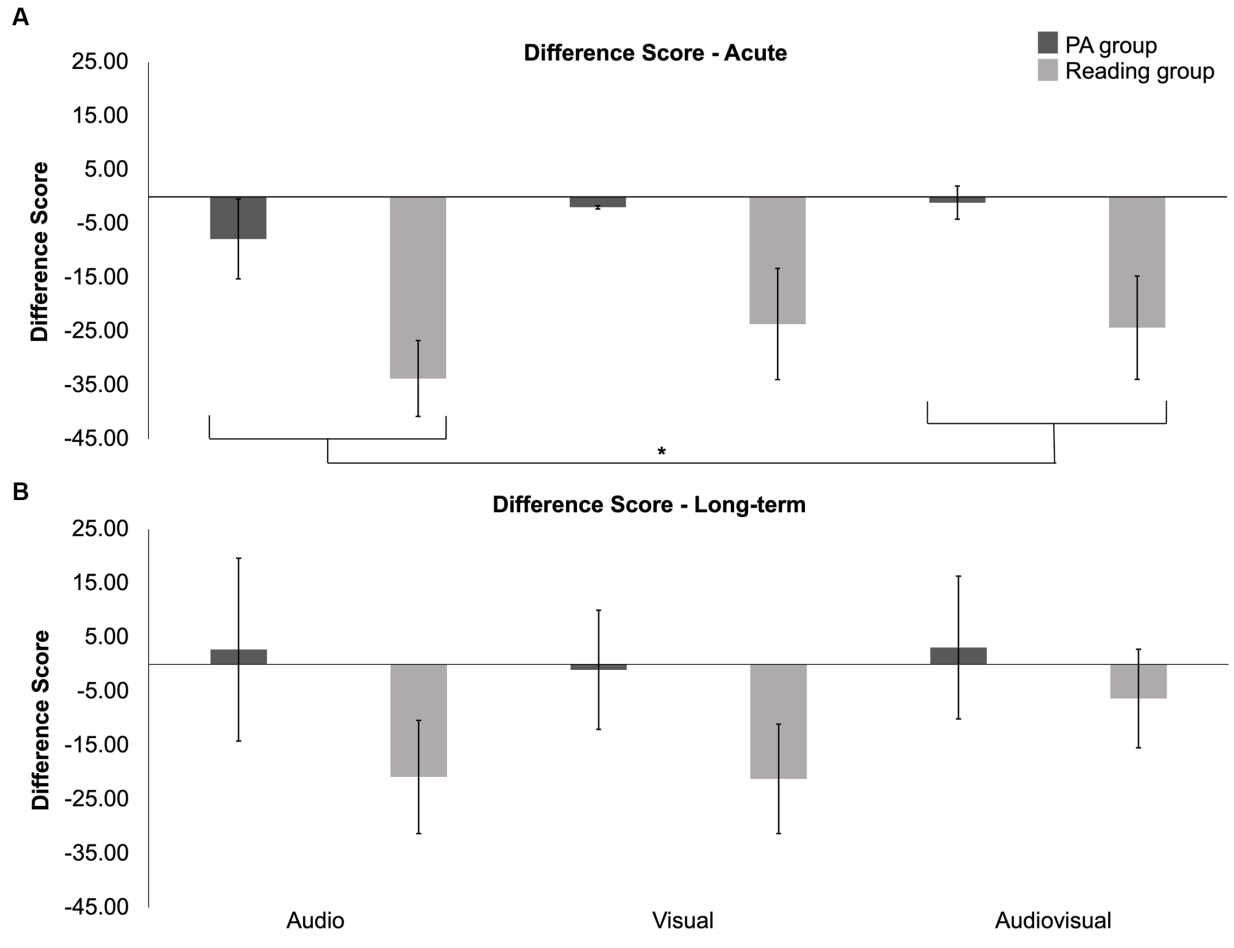
Scores calculated by subtracting pre-session response time for auditory, visual, and audiovisual stimuli from post-session response time. Response times are collapsed across 3 times (session 1 post - pre-session 1, session 2 post - pre-session 2, and session 3 post - pre-session 3) in panel **(A)** and 1 time (post - baseline) in panel **(B)**. Error bars indicate the SEM.

To further investigate the data, difference scores were used to assess long-term and acute effects. A 2 (group) × 3 (modality) mixed-design ANOVA was conducted to assess long-term intervention effects on multisensory processing using difference scores. No significant main effect of group (*F* (1, 24) = 1.356, *p* = 0.256; *η*^2^_p_ = 0.053), modality (*F* (2, 48) = 1.086, *p* = 0.346; *η*^2^_p_ = 0.043), or interaction between group and modality (*F* (2, 48) = 0.593, *p* = 0.556; *η*^2^_p_ = 0.024) was found. Additionally, a 2 (group) × 3 (time: post-session 1 - pre-session 1, post-session 2 - pre-session 2, post-session 3 - pre-session 3) × 3 (modality) mixed-design ANOVA was conducted to assess acute intervention effects on multisensory processing. This analysis did not reveal significant main effects of group (*F* (1, 23) = 3.445, *p* = 0.076; *η*^2^_p_ = 0.130), modality (*F* (2, 46) = 2.206, *p* = 0.122; *η*^2^_p_ = 0.088), or time (*F* (2, 46) = 1.726, *p* = 0.189; *η*^2^_p_ = 0.070). However, a significant interaction between time and modality (*F* (2.957, 68.002) = 3.157, *p* = 0.018; *η*^2^_p_ = 0.121) was found. Pairwise comparisons investigating the interaction between time and modality revealed that the interaction was driven by the auditory modality exhibiting a larger difference when pre-session 3 scores were subtracted from post-session 3 scores (i.e., greater improvement; mean = −27.54, s.e. = 19.86) compared to the session 3 difference scores obtained for the audiovisual modality (*p* = 0.027; mean = −1.768, s.e. = 4.363). Although not significant, the main effect of group approached significance, and post-hoc pairwise comparisons revealed that those in the reading condition showed a larger difference in performance (mean = −27.25, s.e. = 4.84) compared to those who engaged in physical activity (mean = −3.63, s.e. = 2.55). Figure 8 displays both acute (panel a) and long-term (panel b) difference scores. Here for the acute sessions, a significant interaction between time and modality was found, potentially driven by a larger difference for the auditory modality compared to the audiovisual modality over session 3 (*p* = 0.027). Although not significant, the reading group showed a larger difference in performance (i.e., greater improvement) compared to those who engaged in physical activity. No other significant effects or interactions were found. As we did not expect reading to positively affect performance, we suspected that here too the nearing-significant effect of group was driven by age, and indeed when age was added as a covariate, the analysis revealed no main effect of group (*F* (1, 22) = 2,273, *p* = 0.146; *η*^2^_p_ = 0.094). No other effects were significant. These results indicate that the effect of reading leading to a greater difference in performance (i.e., greater improvement) may be explained by age. Those in the reading group may have exhibited greater improvement as there is greater room for improvement with older age.

See Supplementary material for analyses conducted with median response times that confirm and supplement our findings from mean response times.

### Area under the curve

**Figure 9:**
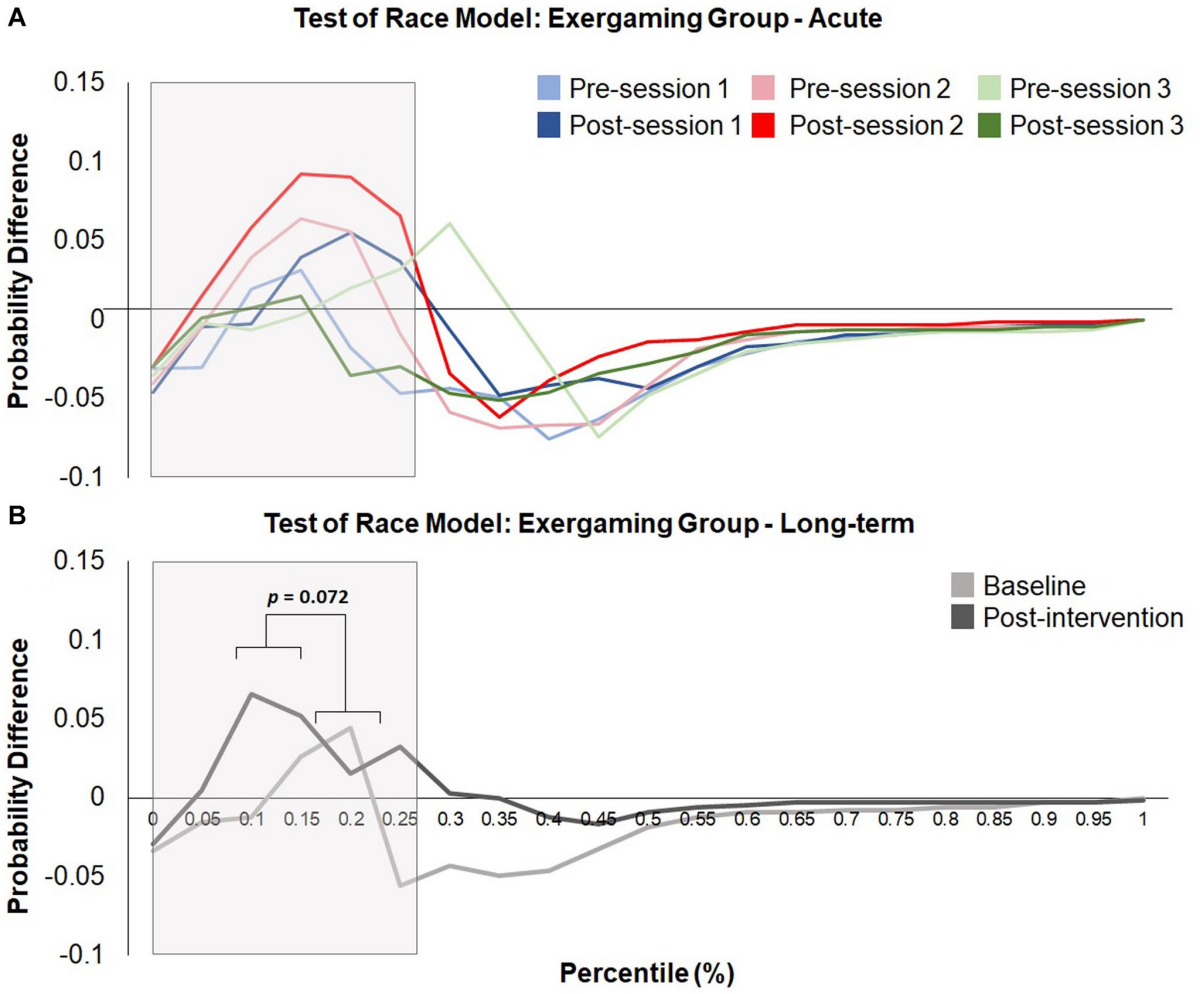
Test of the race model for those who engaged in physical activity showing the probability difference wave, where the predicted CDF is subtracted from the observed CDF for **(A)** acute changes (sessions 1, 2, and 3) and **(B)** long-term differences (i.e., baseline and post-intervention). The grey box indicates the area analyzed. A near-significant effect of time from the acute analysis revealed that the area under the curve increased after both interventions (*p* = 0.072). No further significant effects or interactions were found.

**Figure 10:**
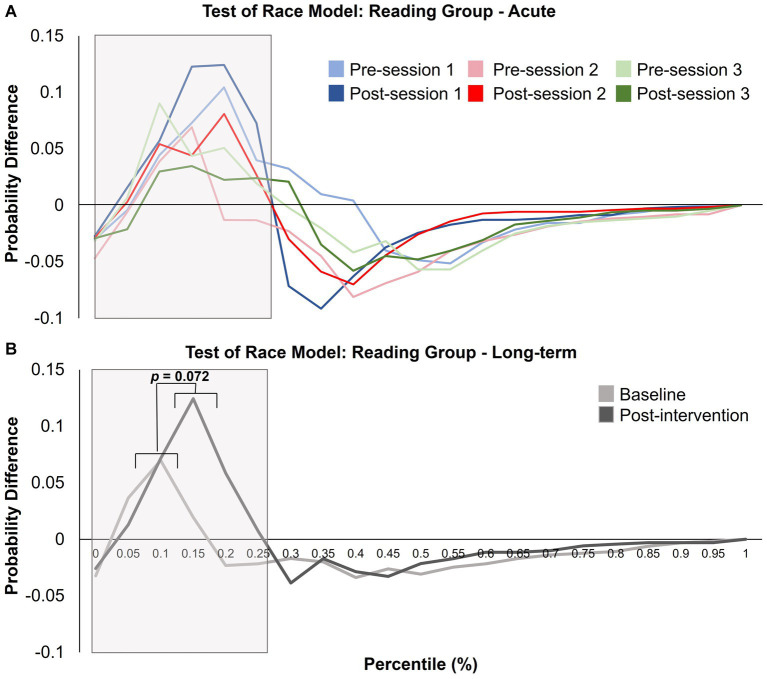
Test of the race model for the reading group showing the probability difference wave, where the predicted CDF is subtracted from the observed CDF for **(A)** acute changes (sessions 1, 2, and 3) and **(B)** long-term differences (i.e., baseline and post-intervention). The grey box indicates the area analyzed. A near-significant effect of time from the acute analysis revealed that the area under the curve increased after both interventions (*p* = 0.072). No further significant effects or interactions were found.

To investigate the long-term effects of the interventions, a 2 (group) × 2 (time) mixed-design ANOVA was conducted. This analysis revealed a near-significant effect of time (*F* (1, 25) = 3.526, *p* = 0.072; *η*^2^_p_ = 0.124) but did not show a main effect of group (*F* (1, 25) = 0.859, *p* = 0.363; *η*^2^_p_ = 0.033) or a significant interaction between group and time (*F* (1, 25) = 0.10, *p* = 0.923; *η*^2^_p_ < 0.001). Pairwise comparisons investigating the near-significant effect of time revealed an increase in the area under the curve post-intervention compared to baseline, indicating increased violations post-intervention. See Figures 9 and 10 for the probability difference waves. This section concludes the analyses used to assess the effects of intervention on race model violations (hypothesis 3). The following exploratory analyses investigate potential acute changes and difference scores obtained from longitudinal and acute sessions.

A 2 (group) × 6 (time) mixed-design ANOVA investigating the acute effects of intervention on AUC revealed no significant effect of group (*F* (1, 23) = 1.332, *p* = 0.260; *η*^2^_p_ = 0.055) or time (*F* (3.531, 81.209) = 1.913, *p* = 0.124; *η*^2^_p_ = 0.077). Additionally, no significant interaction between group and time was found (*F* (3.531, 81.209) = 0.931, *p* = 0.442; *η*^2^_p_ = 0.039). See Supplementary Figures S11, 12 for the graphical representation of the acute and long-term area under the curve for the physical activity and reading interventions, respectively.

To further investigate the long-term effects of intervention on AUC using difference scores, an independent t-test was conducted, which did not reveal a significant difference between the two groups (*t* (25) = 0.098, *p* = 0.923; *Cohen’s d* = 0.038). Difference scores were also used to assess acute effects. A 2 (group) × 3 (time: post-session 1 - pre-session 1, post-session 2 - pre-session 2, and post-session 3 - pre-session 3) mixed-design ANOVA investigating acute effects failed to reveal a significant main effect of group (*F* (1, 24) = 0.039, *p* = 0.846; *η*^2^_p_ = 0.002) or time (*F* (2, 48) = 1.829, *p* = 0.172; *η*^2^*_p_* = 0.071). Furthermore, no significant interaction between group and time was found (*F* (2, 48) < 0.01, *p* = 0.999; *η*^2^_p_ < 0.001). See Supplementary Figure S17 for both the acute (panel A) and long-term (panel B) difference scores from those in the physical activity and reading interventions.

## Discussion

Our study aimed to investigate the effects of a physical activity intervention in a VR setting on perceptual processing compared to a reading control condition. Initial analyses revealed that those who engaged in physical activity in a VR environment exhibited higher accuracy scores on the SIFI task (acute effect) and faster response times on the audiovisual RT task (both chronic and acute effect). The significant improvements in perceptual processing in both the experimental and control groups suggest that these interventions may positively impact multisensory processing.

Apart from group differences, time was also a significant factor of interest as data was collected across multiple sessions (baseline and post-intervention for all tasks and three additional pre- and post-sessions for the RT and SIFI tasks in between) to investigate chronic and acute effects of physical activity in VR or reading on multisensory processing. Starting with the chronic effects observed for the SIFI, we found that accuracy to the illusory condition was significantly lower at baseline as compared to post-intervention, suggesting that susceptibility to the illusory condition can decrease either because of repetition effects or because of the interventions that each group was exposed to. Further, the difference score analysis revealed that difference in accuracy to the illusory condition was larger than that for the 2 flash 2-beep condition suggesting that repetition or exposure to our experimental and control conditions is more likely to impact components of perceptual performance that have greater potential for improvement. Further evidence for such a process is provided by the near-significant effect of group for the visual-only trials of the SIFI, where those in the reading group showed a larger difference in performance after 6 weeks of intervention. Our acute-analysis results from the RT task also indicate larger differences on trials with greater room for improvement, where although the mean response times to audiovisual trials were significantly faster than auditory and visual trials across time, the auditory modality showed a larger difference in performance as compared to the audiovisual modality. Additionally, both mean and median response time difference scores investigating acute and long-term effects also revealed that those in the reading group showed larger improvement (i.e., greater difference score) as compared to those in the experimental group. As the reading group had significantly longer response times to all modalities and showed a greater reduction in response time as compared to the experimental group, this finding further suggests the potential of our interventions or repetition to target areas or populations that are most in need of improvement.

Although not significant, we found that those in the reading group had wider TBWs at baseline as compared to post-intervention (i.e., greater improvement) for the SJ and TOJ tasks.I. These results suggest that either reading and engaging with VR can directly affect the width of the TBW, or that exposure to, and improvement on the SIFI and RT tasks, may have beneficial transferable effects. Previous research provides some guidance related to transfer effects. A study conducted by Setti et al. (2014) aimed to determine the impact of perceptual training on older adults where they trained twenty-four individuals to judge the temporal order of auditory and visual stimuli using the TOJ task, while providing feedback after each trial, over five consecutive days. They found that the majority (eighteen of the twenty-four) of the participants were significantly more accurate on the TOJ task on the fifth as compared to the first day. Additionally, the researchers aimed to determine whether training participants on the TOJ task would reduce susceptibility to the SIFI and although training on the TOJ task did not improve susceptibility to the SIFI for all the stimulus onset asynchronies, significant improvement appeared for the longest SOA of 270 ms.

Our results additionally revealed that (prior to our covariate analysis) the control group showed greater improvement (i.e., reduction) in response time compared to the experimental group. However, when age was added as a covariate in our analysis, this difference disappeared. These results indicate that older adults are more likely to benefit from interventions, possibly due to repetition or transfer effects, because they have greater room for improvement (Powers III et al., 2009, 2016). Future research should employ a more systematic approach to participant selection, matching age and sex between intervention and control groups.

As single-bouts of exercise have been shown to impact not only higher-order cognitive function (Audiffren et al., 2008; Chang et al., 2012; McSween et al., 2018; pontifex et al., 2019) but also sensory processing (O’brien et al., 2017; Basharat and Barnett-Cowan, 2023), it is not surprising that our physical activity intervention (‘Seas the Day’) affected multisensory processing as assessed *via* the RT and SIFI tasks. One potential explanation for changes observed through exercise in multisensory processing could be related to increases in Gamma-aminobtyric acid (GABA), the chief inhibitory neurotransmitter in the central nervous system. GABA tends to decrease in concentration with aging and indeed, Gao et al. (2013) found that the levels of GABA are reduced in frontal and parietal regions by approximately 5% per decade of life. Such a reduction in GABA can reduce the brain’s ability to ignore or inhibit the integration of erroneous cues and can potentially increase the difficulty in discriminating the temporal order of information. GABA levels have been found to increase in concentration not only with chronic exercise but also following acute bouts of exercise (Maddock et al., 2016; Li et al., 2017). Indeed, in a study conducted by Maddock et al. (2016), GABA levels were found to increase significantly after vigorous exercise (80% of predicted maximal heart rate) in 38 young adults (mean age = 26.68). It is important to note however that although there is evidence to indicate that single bouts of aerobic exercise can increase GABA concentration, which may have an impact on multisensory processing, most of the neurophysiological research has been conducted with high or moderate intensity exercise, which is unlike the intensity utilized in this intervention. The participants in this study were asked to exert light to moderate effort and most participants reported exerting light effort. This can help to explain the lack of group differences observed for the SJ and TOJ tasks between the control and experimental groups. However, a meta-analysis conducted by Chang et al. (2012) did find that 20 min of light exercise can induce cognitive enhancement as long as cognition is tested within the first 20 min following exercise, which may help to explain the effects that were indeed observed. It is interesting however that the larger differences between the mean and median scores were observed for the reading group.

Although changes in multisensory processing were expected from engaging in the experimental intervention, the unexpected improvements from engaging in reading may arise from the fact that reading is thought to be a relaxing activity which has been shown to improve mental health, maintain cognitive abilities, reduce the risk of mortality, and reduce stress in young and older adults (Rizzolo et al., 2009; Bavishi et al., 2016; Levine et al., 2022). Indeed, in a study conducted by Rizzolo et al. (2009), a single session of 30 min of reading was found to reduce stress by reducing elevated systolic blood pressure, diastolic blood pressure, and heart rate in 24 young adults (mean age = 23). Most interestingly, it was found that 30-min of reading had similar effects to 30-min of yoga and watching a humorous video. In an older study, 60 min of reading was similarly found to reduce anxiety, heart rate, and blood pressure in 24 adults (mean age = 36.2), however in this study, Tai-Chi was found to have superior effects (Jin, 1992). One possible mechanism through which reading can reduce stress is *via* easing of tension in the muscles of readers, which may occur when an individual becomes immersed into the topic of interest. Another potential mechanism, not dissimilar to exercise, is the GABergic system, where reading may reduce stress through the modulation of GABA (refer to de Souza Spinosa et al., 2002 and Lydiard, 2003 that indicate an increase in GABA with a reduction in stress and anxiety). The evidence presented here and above indicates that the GABAergic system may underlie the changes in multisensory processes observed in this study and warrants further investigation.

While reading served as a control condition in our study, it may not be optimal for researchers investigating multisensory processing, as reading is considered a multisensory activity (Boerma et al., 2016; Brosch, 2018). Notably, the additional covariate analyses (with age as a factor) rendered the difference between the two groups insignificant. Future research should explore alternative control conditions less likely to engage multisensory processing and systematically investigate control conditions utilized by the exercise literature (e.g., stretching, socializing with others, disengaged, etc.; Pontifex et al., 2019). Additionally, future researchers may consider increasing the intensity of their exergaming intervention, as moderate to vigorous intensity has been found to optimally affect cognitive processing following both acute (Chang et al., 2012; McSween et al., 2018; Pontifex et al., 2019) and chronic physical exercise (Erickson et al., 2011, 2019). Finally, including the perceived enjoyment questionnaire for both groups would provide a more comprehensive comparison between the interventions.

It is crucial to interpret these results with caution, however, due to potential group differences, such as the older age of participants and higher number of females in the reading group. The age discrepancy could have provided greater potential for improvement in the reading group, as evidenced by the larger difference scores obtained for the SIFI and RT tasks. Prior research has demonstrated that the age of the perceiver directly impacts the temporal binding window (TBW) and susceptibility to illusions, which can be associated with various adverse outcomes (Poliakoff et al., 2006; Setti et al., 2011a,b; Chan et al., 2014a,b; Bedard and Barnett-Cowan, 2016; Basharat et al., 2018, 2019). To mitigate the effects of age between the control and experimental groups, we added age as a covariate each time a main effect of the group was found, revealing that age significantly affected performance in this study. An additional limitation of this study is the learning effects that may arise from repetition of the SIFI and RT tasks. Although learning effects are inevitable, especially for the RT task, future studies can consider the utilization of randomization of SOAs for each session for the SIFI task to reduce such effects from affecting their results.

In conclusion, our study aimed to demonstrate that participation in our co-designed physical activity intervention, compared to a reading control, would reduce susceptibility to the SIFI, reduce the width of the TBW for both the SJ and TOJ tasks, and reduce response time while increasing race model violations. Although we found evidence for a reduction in susceptibility to the SIFI and a reduction in response time, we did not find any evidence of change for the SJ and TOJ tasks or a change in race model violations. More importantly, we found that the older age of our participants in the reading group was the driving factor for the observed group differences. Researchers should consider alternative control conditions and ensure that age and sex are matched between intervention and control groups to provide a more accurate comparison. Regardless of this limitation, our study demonstrates that both physical activity in a VR setting and reading interventions can influence perceptual processing. Despite potential group differences and limitations, our findings contribute valuable insights into the impact of these interventions on multisensory processing.

Our research indicates that VR may be a useful tool to investigate and subsequently impact multisensory processing, while promoting physical activity. As we aimed to create an intervention that was accessible to all older adults with intact auditory and visual processing, we were therefore limited to light-to-moderate intensity of physical activity. However, researchers hoping to utilize this tool in the future may see larger effects with exercise intervention requiring a higher intensity of exertion. Future research should focus on exploring the underlying mechanisms, such as the GABAergic system, that may contribute to the observed changes in perceptual processing. Additionally, it is recommended that future researchers investigate the longer-term effects (i.e., longer than 6 weeks) of these interventions on multisensory processing and cognitive function in older adults with and without cognitive impairment.

## Author’s note

Physical activity plays a crucial role in maintaining and improving cognitive and perceptual processes, particularly in older adults. Perceptual processes, such as multisensory integration, refer to the ability to combine and interpret sensory information from various sources (e.g., vision, hearing, touch) to better understand and interact with the environment. Research has demonstrated that regular physical activity can enhance these perceptual processes, leading to improvements in cognitive function, motor learning, and overall well-being. In this exploratory, pilot non-randomized control trial, we investigated the effects exergaming in a virtual reality as compared to our reading control during the COVID-19 pandemic to encourage participation in physical activity. We found that exergaming and reading interventions can influence perceptual processing as tested *via* four different tasks including the Sound-induced flash illusion. More importantly, however, we found that the older age of our participants in the reading group was the driving factor for the observed group differences. Regardless of this limitation, our study demonstrates that perceptual processes are malleable and can be influenced by both reading and exergaming interventions. Despite potential group differences and limitations, our findings contribute valuable insights into the impact of these interventions on multisensory processing.

## Data availability statement

The raw data supporting the conclusions of this article will be made available by the authors, without undue reservation.

## Ethics statement

The studies involving humans were approved by University of Waterloo’s Human Research Ethics Board. The studies were conducted in accordance with the local legislation and institutional requirements. The ethics committee/institutional review board waived the requirement of written informed consent for participation from the participants or the participants’ legal guardians/next of kin because Verbal consent was obtained from all participants in lieu of written consent.

## Author contributions

All authors listed have made a substantial, direct, and intellectual contribution to the work, and approved it for publication.

## Footnotes

^1^ https://www.oculus.com/experiences/quest/4164068860279573
