# Supplementary material for: Virtual reality as a tool to explore multisensory processing before and after engagement in physical activity
Source: Front Aging Neurosci. 2023 Nov 2;15:1207651. doi: 10.3389/fnagi.2023.1207651 (PMC10652573; doi:10.3389/fnagi.2023.1207651)
Supplement: Supplementary file 1 [file Data_Sheet_1.docx]

| Condition | #Beeps | #Flashes | SOAs (ms) |
| --- | --- | --- | --- |
| Multisensory - illusory condition (2B1F) | 2 | 1 | -230, -150, -70, 70, 150, 230 |
| Multisensory - control condition (2B1F) | 2 | 2 | 70, 150, 230 |
| Multisensory - control condition (1B1F) | 1 | 1 | 0 |
| Unimodal visual (0B2F) | 0 | 2 | 70, 150, 230 |
| Unimodal auditory (2B0F) | 2 | 0 | 70, 150, 230 |

*Supplementary Table 1:* This table provides details regarding the parameters utilized for each condition included in the Sound-Induced Flash Illusion (SIFI). SOA = Stimulus Onset Asynchrony, where negative values indicate that a beep preceded the flash-beep pair.


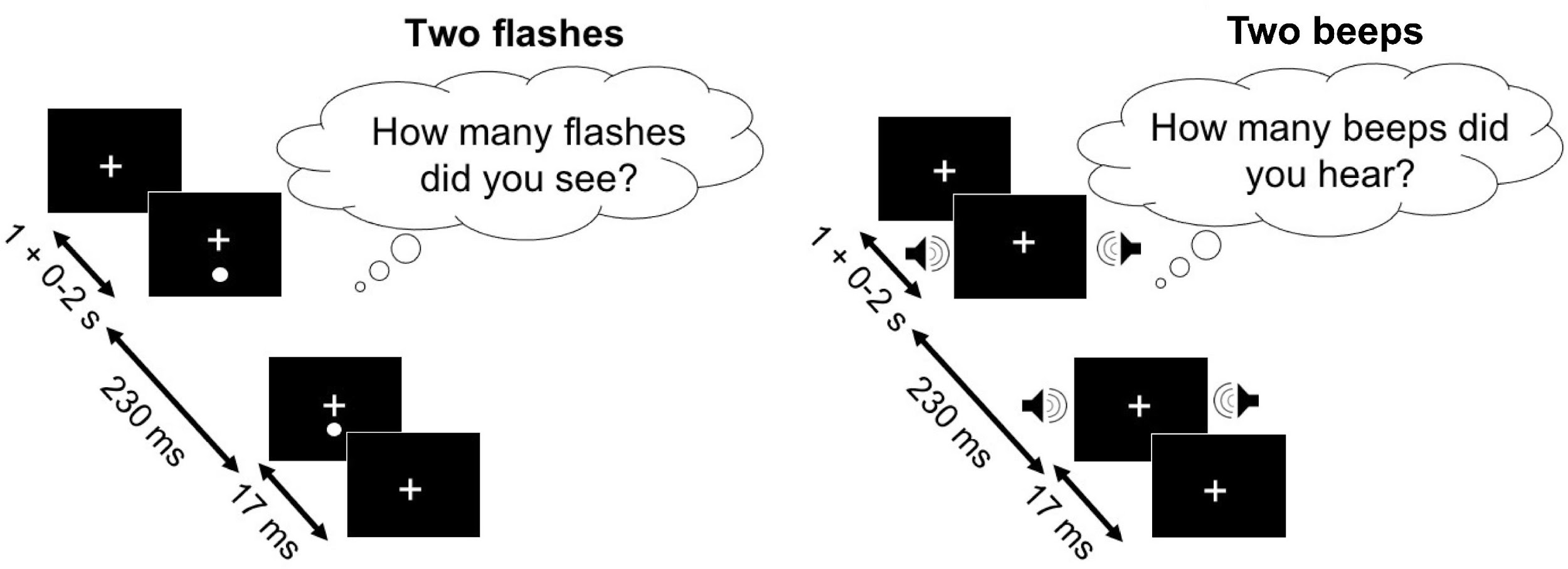


*Supplementary Figure 1:* Sound-induced Flash Illusion. Visual-only (left) and audio-only (right) trials were presented to participants with SOAs of 70,150, and 230 ms. In both tasks, the first stimulus of the pair could appear 1-3 seconds after the fixation cross, and the second stimulus appeared between 70 and 230 ms after the first stimulus.


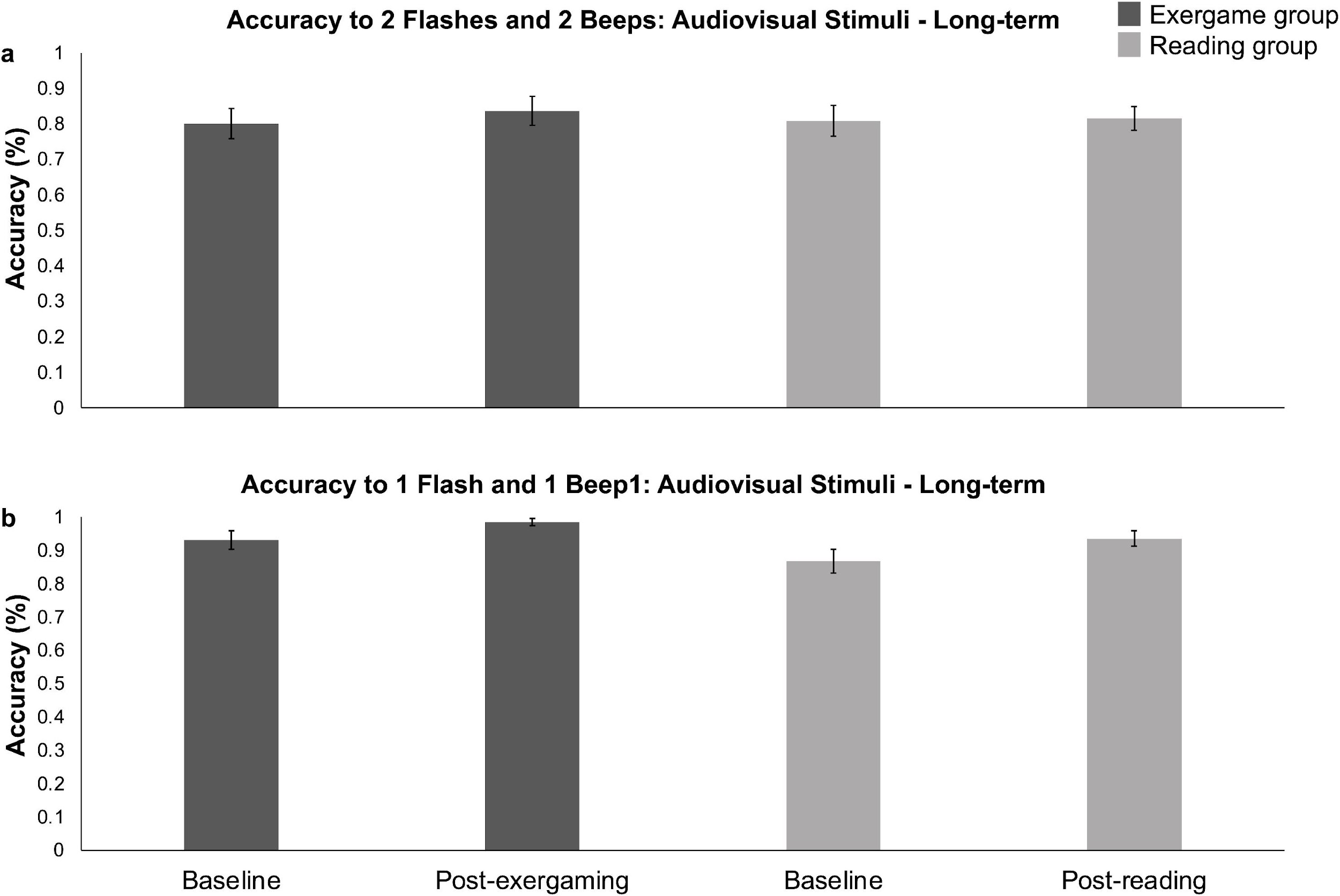


*Supplementary Figure 2:* Accuracy for 2-flashes/2-beeps and 1-flash/1-beep conditions of the Sound-induced Flash Illusion during baseline and post-intervention sessions in the physical activity and reading groups. Panel a: 2-flashes/2-beeps condition accuracy. Panel b: 1-flash/1-beep condition accuracy. Significantly higher accuracy was found for the 1-flash/1-beep condition compared to the illusory and overall accuracy conditions (*p* = 0.002 and *p* = 0.019, respectively). Error bars represent SEM.


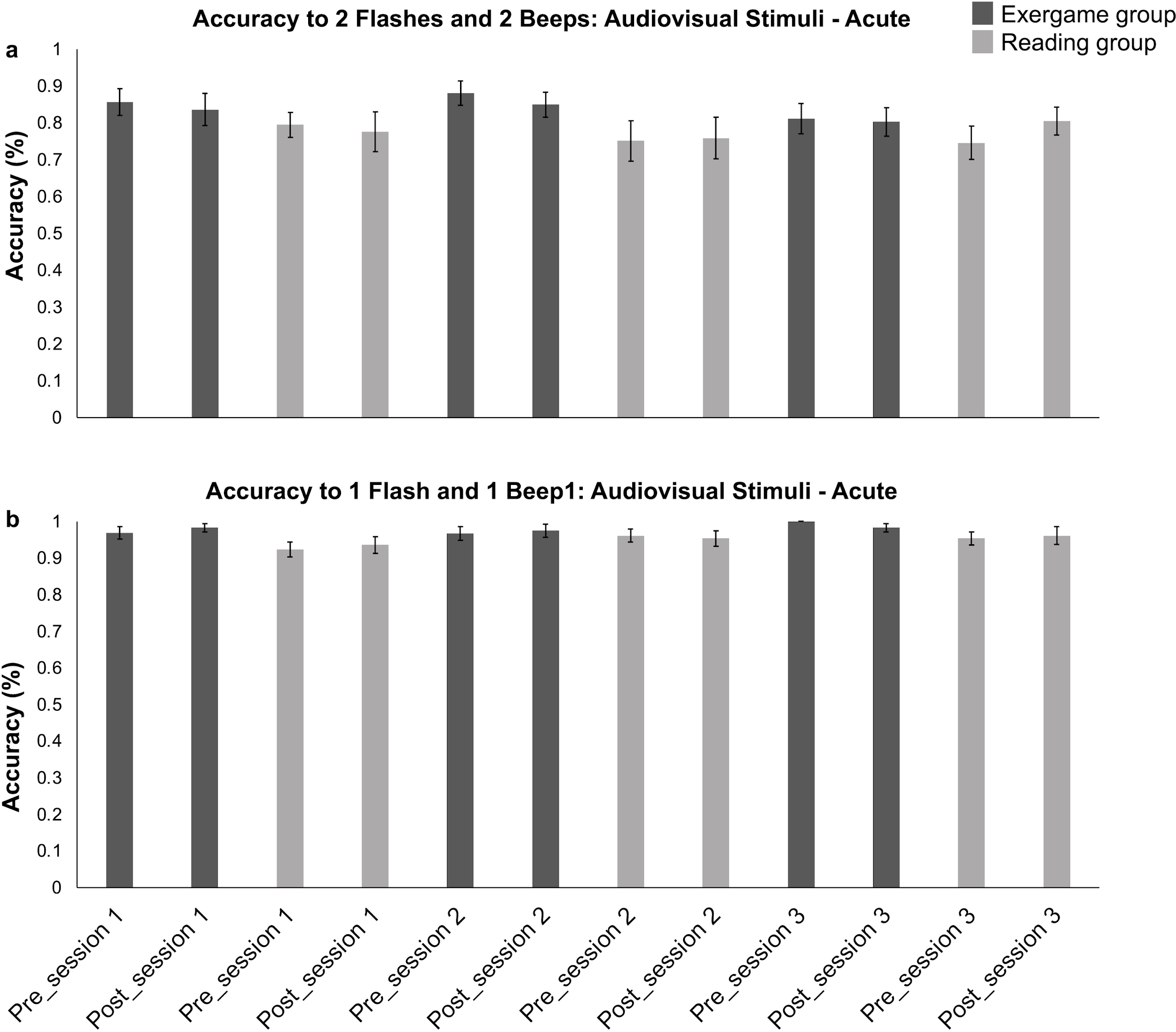


*Supplementary Figure 3:* Accuracy for 2-flashes/2-beeps and 1-flash/1-beep conditions of the Sound-induced Flash Illusion in the physical activity and reading groups. Panel a: 2-flashes/2-beeps condition accuracy. Panel b: 1-flash/1-beep condition accuracy. The 1-flash/1-beep condition had significantly higher accuracy than overall, illusory, and 2-flashes/2-beeps conditions (*p* = 0.003, *p* = 0.003, and *p* = 0.014, respectively). Error bars represent SEM.

| Time*Condition | Time*Condition | Mean Difference | SE | t | Cohen's d | pbonf |
| --- | --- | --- | --- | --- | --- | --- |
| T 1-pre, Overall | T 1-pre, 1 flash | -0.217 | 0.043 | -5.002 | -1.479 | <0.001 |
|  | T 1-post, 1 flash | -0.227 | 0.044 | -5.199 | -1.548 | <0.001 |
|  | T 2-pre, 1 flash | -0.232 | 0.044 | -5.314 | -1.582 | <0.001 |
|  | T 2-post, 1 flash | -0.232 | 0.044 | -5.314 | -1.582 | <0.001 |
|  | T 3-pre, 1 flash | -0.252 | 0.044 | -5.772 | -1.718 | <0.001 |
|  | T 3-post, 1 flash | -0.252 | 0.044 | -5.772 | -1.718 | <0.001 |
| T 1-post, Overall | T 1-pre, 1 flash | -0.203 | 0.044 | -4.649 | -1.384 | 0.002 |
|  | T 1-post, 1 flash | -0.213 | 0.043 | -4.910 | -1.452 | <0.001 |
|  | T 2-pre, 1 flash | -0.218 | 0.044 | -4.993 | -1.486 | <0.001 |
|  | T 2-post, 1 flash | -0.218 | 0.044 | -4.993 | -1.486 | <0.001 |
|  | T 3-pre, 1 flash | -0.238 | 0.044 | -5.451 | -1.623 | <0.001 |
|  | T 3-post, 1 flash | -0.238 | 0.044 | -5.451 | -1.623 | <0.001 |
| T 2-pre, Overall | T 1-pre, 1 flash | -0.168 | 0.044 | -3.859 | -1.149 | 0.049 |
|  | T 1-post, 1 flash | -0.179 | 0.044 | -4.088 | -1.217 | 0.021 |
|  | T 2-pre, 1 flash | -0.183 | 0.043 | -4.230 | -1.251 | 0.012 |
|  | T 2-post, 1 flash | -0.184 | 0.044 | -4.203 | -1.251 | 0.013 |
|  | T 3-pre, 1 flash | -0.203 | 0.044 | -4.661 | -1.387 | 0.002 |
|  | T 3-post, 1 flash | -0.203 | 0.044 | -4.661 | -1.387 | 0.002 |
| T 2-post, Overall | T 1-post, 1 flash | -0.171 | 0.044 | -3.917 | -1.166 | 0.040 |
|  | T 2-pre, 1 flash | -0.176 | 0.044 | -4.031 | -1.200 | 0.026 |
|  | T 2-post, 1 flash | -0.176 | 0.043 | -4.057 | -1.200 | 0.024 |
|  | T 3-pre, 1 flash | -0.196 | 0.044 | -4.489 | -1.336 | 0.004 |
|  | T 3-post, 1 flash | -0.196 | 0.044 | -4.489 | -1.336 | 0.004 |
| T 3-pre, Overall | T 3-pre, 1 flash | -0.178 | 0.043 | -4.103 | -1.213 | 0.020 |
|  | T 3-post, 1 flash | -0.178 | 0.044 | -4.077 | -1.213 | 0.022 |
| T 3-post, Overall | T 2-pre, 1 flash | -0.168 | 0.044 | -3.859 | -1.149 | 0.049 |
|  | T 2-post, 1 flash | -0.169 | 0.044 | -3.859 | -1.149 | 0.049 |
|  | T 3-pre, 1 flash | -0.189 | 0.044 | -4.317 | -1.285 | 0.008 |
|  | T 3-post, 1 flash | -0.188 | 0.043 | -4.345 | -1.285 | 0.008 |
| T 1-pre, Illusion | T 3-pre, 1 flash | -0.137 | 0.029 | -4.653 | -0.932 | 0.001 |
|  | T 3-post, Illusion | 0.112 | 0.029 | -3.802 | -0.761 | 0.047 |
|  | T 1-pre, 2 flash | -0.169 | 0.043 | -3.900 | -1.153 | 0.043 |
|  | T 1-pre, 1 flash | -0.297 | 0.043 | -6.858 | -2.028 | <0.001 |
|  | T 1-post, 1 flash | -0.307 | 0.044 | -7.043 | -2.096 | <0.001 |
|  | T 2-pre, 1 flash | -0.312 | 0.044 | -7.157 | -2.130 | <0.001 |
|  | T 2-post, 1 flash | -0.312 | 0.044 | -7.157 | -2.130 | <0.001 |
|  | T 3-pre, 1 flash | -0.332 | 0.044 | -7.615 | -2.267 | <0.001 |
|  | T 3-post, 1 flash | -0.332 | 0.044 | -7.615 | -2.267 | <0.001 |
| T 1-post, illusion | T 1-pre, 1 flash | -0.267 | 0.044 | -6.108 | -1.818 | <0.001 |
|  | T 1-post, 1 flash | -0.277 | 0.043 | -6.378 | -1.886 | <0.001 |
|  | T 2-pre, 1 flash | -0.282 | 0.044 | -6.451 | -1.920 | <0.001 |
|  | T 2-post, 1 flash | -0.282 | 0.044 | -6.451 | -1.920 | <0.001 |
|  | T 3-pre, 1 flash | -0.302 | 0.044 | -6.909 | -2.057 | <0.001 |
|  | T 3-post, 1 flash | -0.302 | 0.044 | -6.909 | -2.057 | <0.001 |
| T 2-pre, illusion | T 1-post, 1 flash | -0.220 | 0.044 | -5.039 | -1.500 | <0.001 |
|  | T 2-pre, 1 flash | -0.225 | 0.043 | -5.187 | -1.534 | <0.001 |
|  | T 2-post, 1 flash | -0.225 | 0.044 | -5.153 | -1.534 | <0.001 |
|  | T 3-pre, 1 flash | -0.245 | 0.044 | -5.611 | -1.670 | <0.001 |
|  | T 3-post, 1 flash | -0.245 | 0.044 | -5.611 | -1.670 | <0.001 |
| T 2-post, Illusion | T 3-pre, 1 flash | -0.229 | 0.044 | -5.249 | -1.562 | <0.001 |
|  | T 3-post, 1 flash | -0.229 | 0.044 | -5.249 | -1.562 | <0.001 |
|  | T 3-pre, Illusion | -0.171 | 0.044 | -3.913 | -1.165 | 0.040 |
|  | T 2-pre, 1 flash | -0.176 | 0.044 | -4.027 | -1.199 | 0.026 |
|  | T 2-post, 1 flash | -0.176 | 0.044 | -4.027 | -1.199 | 0.026 |
|  | T 3-pre, 1 flash | -0.196 | 0.043 | -4.514 | -1.335 | 0.004 |
|  | T 3-post, 1 flash | -0.196 | 0.044 | -4.485 | -1.335 | 0.004 |
| T 3-post, Illusion | T 1-pre, 1 flash | -0.186 | 0.044 | -4.256 | -1.267 | 0.011 |
|  | T 1-post, 1 flash | -0.196 | 0.044 | -4.485 | -1.335 | 0.004 |
|  | T 2-pre, 1 flash | -0.201 | 0.044 | -4.600 | -1.369 | 0.003 |
|  | T 2-post, 1 flash | -0.201 | 0.044 | -4.600 | -1.369 | 0.003 |
|  | T 3-pre, 1 flash | -0.221 | 0.044 | -5.058 | -1.505 | <0.001 |
|  | T 3-post, 1 flash | -0.221 | 0.043 | -5.091 | -1.505 | <0.001 |
|  | T 1-post, 2 flash | -0.182 | 0.044 | -4.161 | -1.238 | 0.016 |
|  | T 3-post, 1 flash | -0.182 | 0.044 | -4.161 | -1.238 | 0.016 |
|  | T 2-pre, 2 flash | -0.182 | 0.044 | -4.161 | -1.238 | 0.016 |
|  | T 3-post, 1 flash | -0.182 | 0.044 | -4.161 | -1.238 | 0.016 |
|  | T 2-post, 2 flash | -0.168 | 0.044 | -3.855 | -1.148 | 0.050 |
|  | T 2-post, 1 flash | -0.168 | 0.043 | -3.880 | -1.148 | 0.046 |
|  | T 3-pre, 1 flash | -0.188 | 0.044 | -4.314 | -1.284 | 0.009 |
|  | T 3-post, 1 flash | -0.188 | 0.044 | -4.314 | -1.284 | 0.009 |
|  | T 3-pre, 2 flash | -0.177 | 0.044 | -4.046 | -1.204 | 0.024 |
|  | T 2-pre, 1 flash | -0.182 | 0.044 | -4.161 | -1.238 | 0.016 |
|  | T 2-post, 1 flash | -0.182 | 0.044 | -4.161 | -1.238 | 0.016 |
|  | T 3-pre, 1 flash | -0.202 | 0.043 | -4.649 | -1.375 | 0.002 |
|  | T 3-post, 1 flash | -0.202 | 0.044 | -4.619 | -1.375 | 0.002 |
|  | T 3-post, 2 flash | -0.187 | 0.044 | -4.275 | -1.273 | 0.010 |
|  | T 3-post, 1 flash | -0.187 | 0.043 | -4.303 | -1.273 | 0.009 |

*Supplementary Table 2:* Post-hoc comparisons for the audiovisual condition of the Sound-induced Flash Illusion during sessions 1, 2, and 3. Results indicate higher accuracy for the illusory condition during both pre- and post-sessions of session 3 compared to pre-intervention accuracy in session 1 (*p* = 0.001 and *p* = 0.047, respectively). Participants achieved higher accuracy for the 1-flash/1-beep trials compared to overall and illusory conditions (*p* < 0.01 and *p* < 0.05, respectively). T1 = session 1 (week 2), T2 = session 2 (week 4), and T3 = session 3 (week 6). Only significant results are presented.


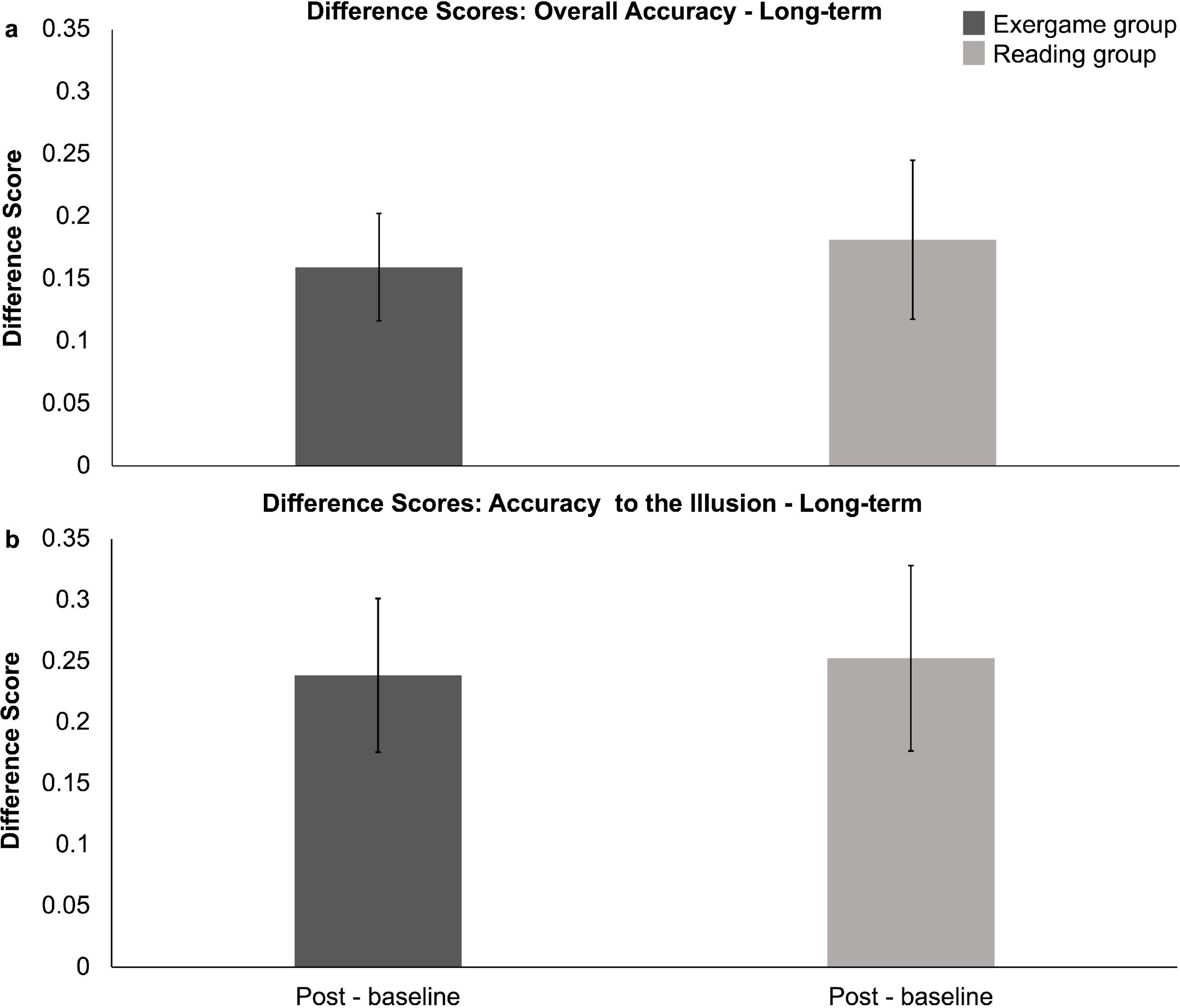


*Supplementary Figure 4:* This figure illustrates the difference scores calculated by subtracting baseline accuracy from post-intervention accuracy for overall accuracy (panel a) and illusory (panel b) conditions for the Sound-induced Flash Illusion. The physical activity group's scores are depicted in dark grey, while the reading group's scores are depicted in light grey. The difference in accuracy for the illusory condition was significantly higher than that for the 2 flash-2 beep (*p* < 0.001) and 1 flash-1 beep conditions (*p* = 0.005), indicating a reduced susceptibility to the illusion and greater improvement after 6 weeks of both engaging in physical activity and reading interventions compared to control conditions. Additionally, pairwise comparisons revealed that the overall accuracy difference was significantly higher than that for the 2 flash-2 beep condition (*p* = 0.035). Error bars indicate the SEM.


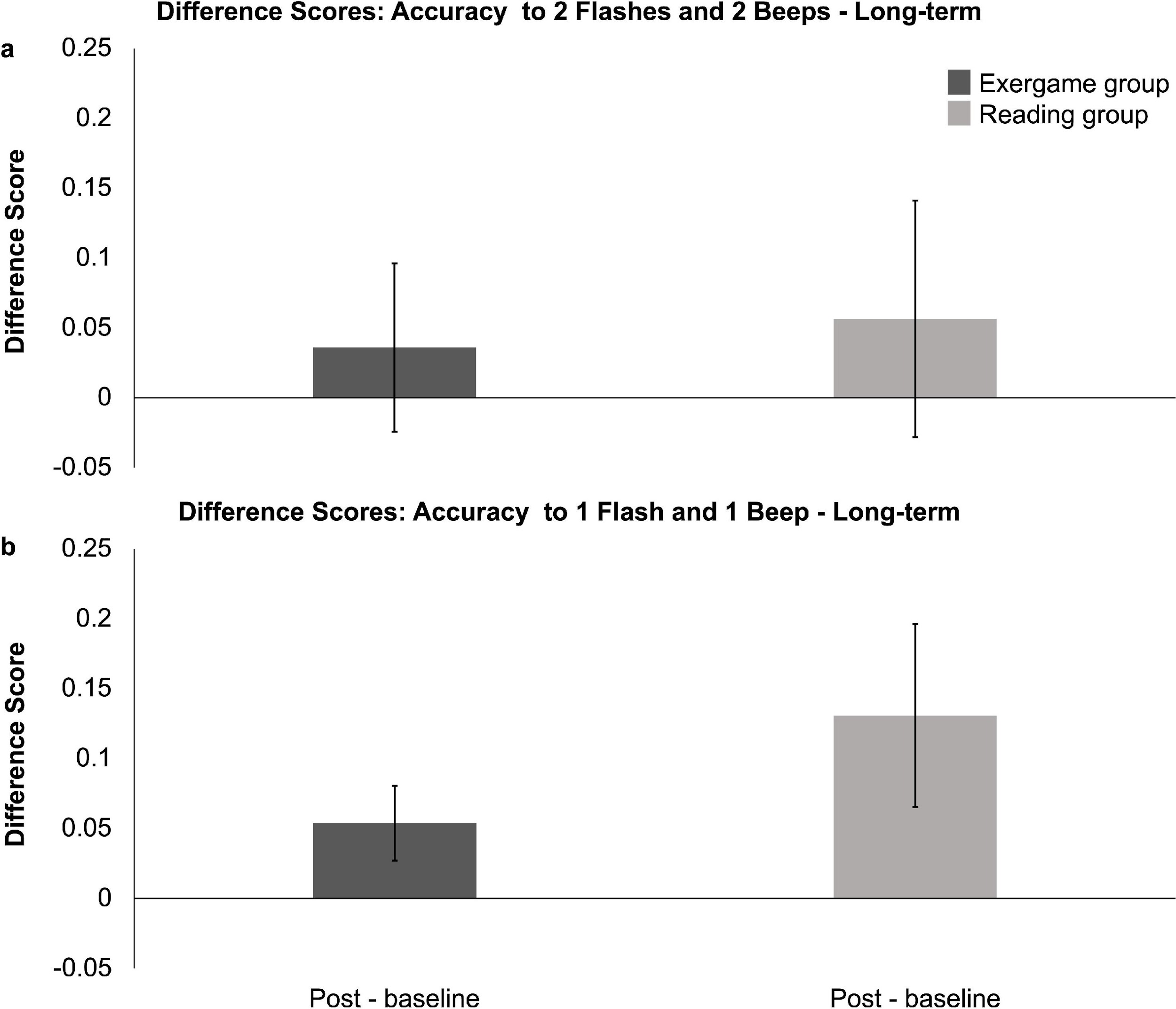


*Supplementary Figure 5:* This figure displays the difference scores calculated by subtracting baseline accuracy from post-intervention accuracy for the 2 flashes-2 beeps (panel a) and 1 flash-1 beep (panel b) conditions for the Sound-induced Flash Illusion. The physical activity group's scores are depicted in dark grey, while the reading group's scores are depicted in light grey. No significant differences were found between sessions. The error bars indicate the SEM.


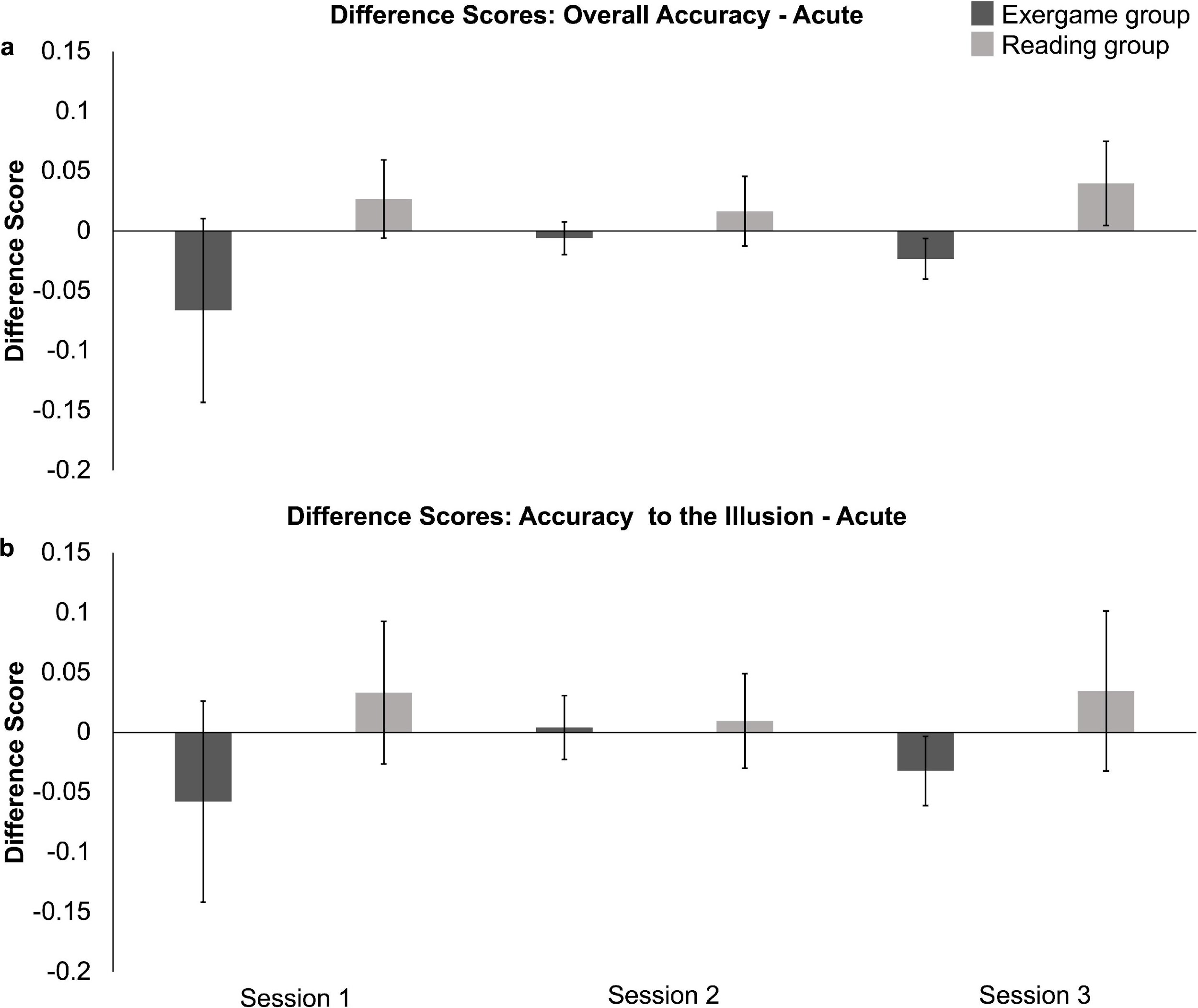


Supplementary *Figure 6:* This figure presents the difference scores calculated by subtracting pre-session accuracy from post-session accuracy for sessions 1, 2, and 3 for overall accuracy (panel a) and accuracy in the illusory (panel b) conditions for the Sound-induced Flash Illusion. The physical activity group's scores are depicted in dark grey, while the reading group's scores are depicted in light grey. No significant differences were found between sessions for both overall accuracy and accuracy in the illusory condition. The error bars indicate the SEM.


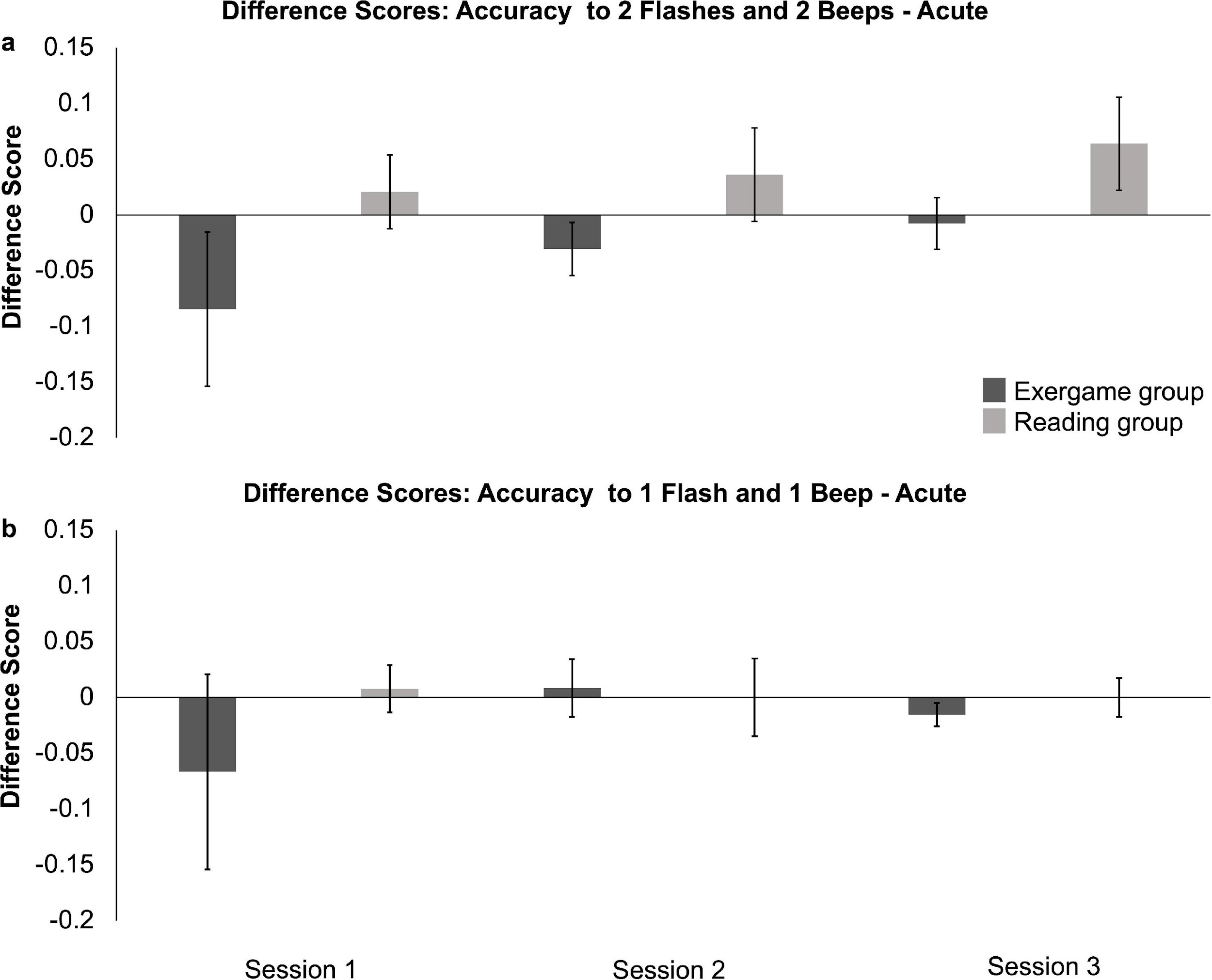


*Supplementary Figure 7:* This figure shows the difference scores calculated by subtracting pre-session accuracy from post-session accuracy for sessions 1, 2, and 3 for the 2 flashes-2 beeps (panel a) and 1 flash-1 beep (panel b) conditions for the Sound-induced Flash Illusion. The physical activity group's scores are depicted in dark grey, while the reading group's scores are depicted in light grey. No significant differences were found between sessions for both the 2 flashes-2 beeps and 1 flash-1 beep conditions. The error bars indicate the SEM.

**SIFI: Unimodal Control Conditions**

A 2 (group) x 2 (time: baseline and post-intervention) mixed-design ANOVA was conducted to assess the long-term effects of intervention on the auditory condition. Due to a violation of Levene's test for Equality of Variance for time, a Friedman test was conducted, which revealed a significant main effect (*χ2* (1) = 7.118, *p* = 0.008). Conover's post-hoc pairwise comparisons investigating the main effect of time showed that accuracy for auditory cues during the post-intervention session was significantly higher than at baseline (p = 0.011). However, no significant main effect of group (*F*(1, 25) 0.803, *p* = 0.379; *η*2_p_ = 0.031) or interaction between group and time (*F*(1, 25) 0.738, *p* = 0.398; *η*2_p_ = 0.029) were found. A 2 (group) x 6 (time) mixed-design ANOVA was conducted to assess acute changes for the auditory condition, which did not reveal significant main effects for group (*F*(1, 18) < 0.01, *p* = 0.987; *η*2_p_ < 0.01) or time (*F*(2.338, 42.081) = 1.203, *p* = 0.315; *η*2_p_ = 0.063). Additionally, no significant interaction between group and time was found (*F*(2.338, 42.081) = 1.941, *p* = 0.150; *η*2_p_ = 0.097). See Supplementary Figure 8 for a graphical representation of both the long-term and acute auditory accuracy obtained from the reading and physical activity groups.

To further investigate the data, difference scores were used to assess long-term and acute effects. An independent t-test was conducted to investigate the long-term effects of physical activity and reading on the auditory condition, and the results failed to find a significant difference between the two groups (*t*(25) = -0.859, *p* = 0.398; *Cohen’s d* = - 0.331). Additionally, a 2 (group) x 3 (time) mixed-design ANOVA was conducted for the auditory cues to investigate any acute changes. The analysis did not reveal a significant effect of group (*F*(1, 21) = 0.930, *p* = 0.346; *η*2_p_ = 0.042) or a significant interaction between group and time (*F*(1.394, 29.271) = 1.806, *p* = 0.189; *η*2_p_ = 0.079). Levene’s test for Equality of Variance was violated, so a Friedman test was conducted, which failed to reveal a main effect of time (*χ2* (2) = 0.552, *p* = 0.759). See Supplementary Figure 10 and Supplementary Figure 11 for graphical representations of both the long-term and acute difference scores, respectively, obtained from both the auditory and visual modalities.

A 2 (group) x 2 (time) mixed-design ANOVA was conducted to assess the long-term effects of intervention for the visual condition. The results did not reveal a significant main effect of group (F (1, 23) = 0.099, p = 0.756; η2 = 0.004) or time (*F*(1, 23) = 0.057, *p* = 0.813; *η*2_p_ = 0.002). Further, no significant interaction between group and time was found (*F*(1, 23)= 1.280, *p* = 0.270; *η*2_p_ = 0.053). Additionally, a 2 (group) x 6 (time) mixed-design ANOVA was conducted to assess the acute effects of intervention for the visual condition. The results did not reveal a significant main effect of group (*F*(1, 18) = 0.048, *p* = 0.829; *η*2_p_ = 0.003) or time (*F*(5, 90) = 0.467, *p* = 0.800; *η*2_p_ = 0.025). Further, no significant interaction between group and time was found (*F*(5, 90) = 0.686, *p* = 0.636; *η*2_p_ = 0.037). Please refer to Supplementary Figure 9 for accuracy scores obtained by those in the physical activity and reading group.

To further investigate the data, difference scores were employed to assess long-term and acute effects. An independent t-test was conducted to examine the long-term effects of physical activity and reading on the visual condition. The results revealed a near-significant difference between the two groups (*t*(25) = -1.837, *p* = 0.078; *Cohen’s d* = - 0.707), with the reading group demonstrating a larger difference in accuracy compared to the physical activity group. Additionally, a 2 (group) x 3 (time) mixed-design ANOVA was conducted for the visual cues to explore acute changes. The analysis did not reveal a significant effect of group (*F*(1, 20) < 0.01, *p* = 0.983; *η*2_p_ < 0.001), time (*F*(2, 40) = 2.286, *p* = 0.115; *η*2_p_ = 0.103), or a significant interaction between group and time (*F*(2, 40) = 1.934, *p* = 0.158; *η*2_p_ = 0.088). Lastly, refer to Figure 10 and Supplementary Figure 11 for graphical representations of both the long-term and acute difference scores, respectively, obtained from both the auditory and visual modalities.


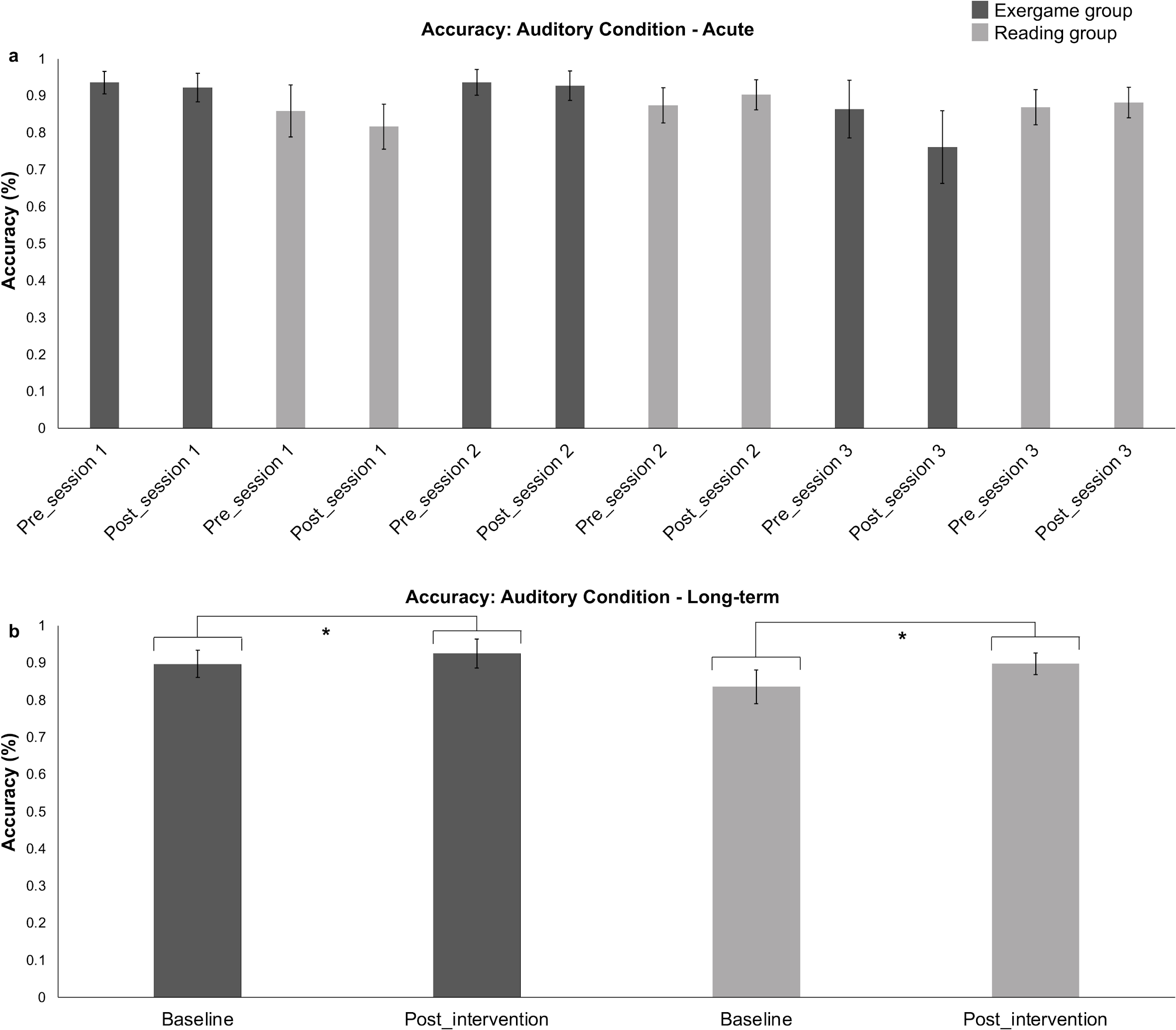


*Supplementary Figure 8:* Acute (panel a) and long-term (panel b) accuracy in the unimodal auditory condition for physical activity (dark grey) and reading (light grey) groups for the Sound-induced Flash Illusion. Post-intervention accuracy was significantly higher than baseline (*p* = 0.011; panel b). Error bars represent SEM.


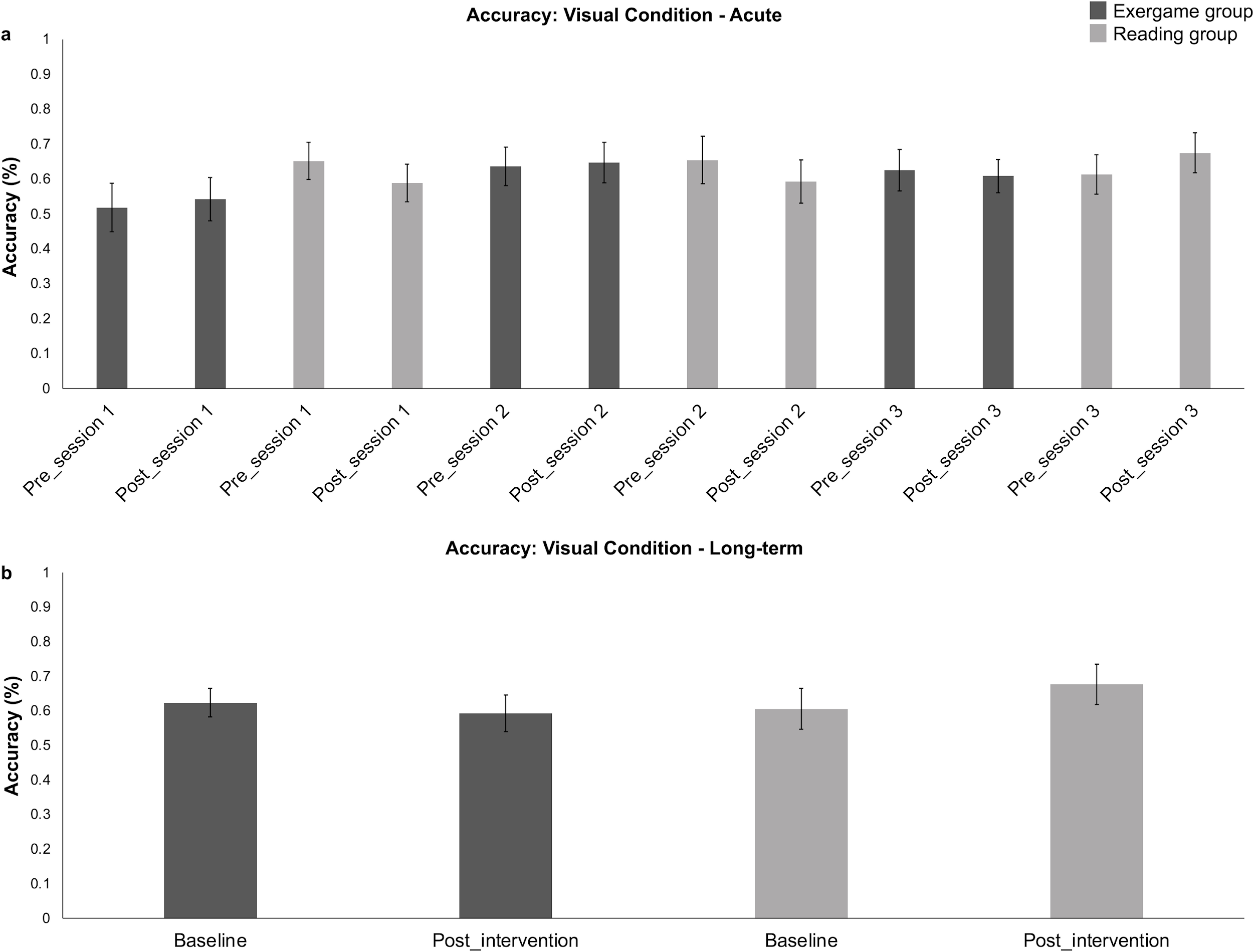


*Supplementary Figure 9:* Acute (panel a) and long-term (panel b) accuracy in the unimodal visual condition for physical activity (dark grey) and reading (light grey) groups for the Sound-induced Flash Illusion. No significant differences were observed in either acute or long-term analyses. Error bars represent SEM.


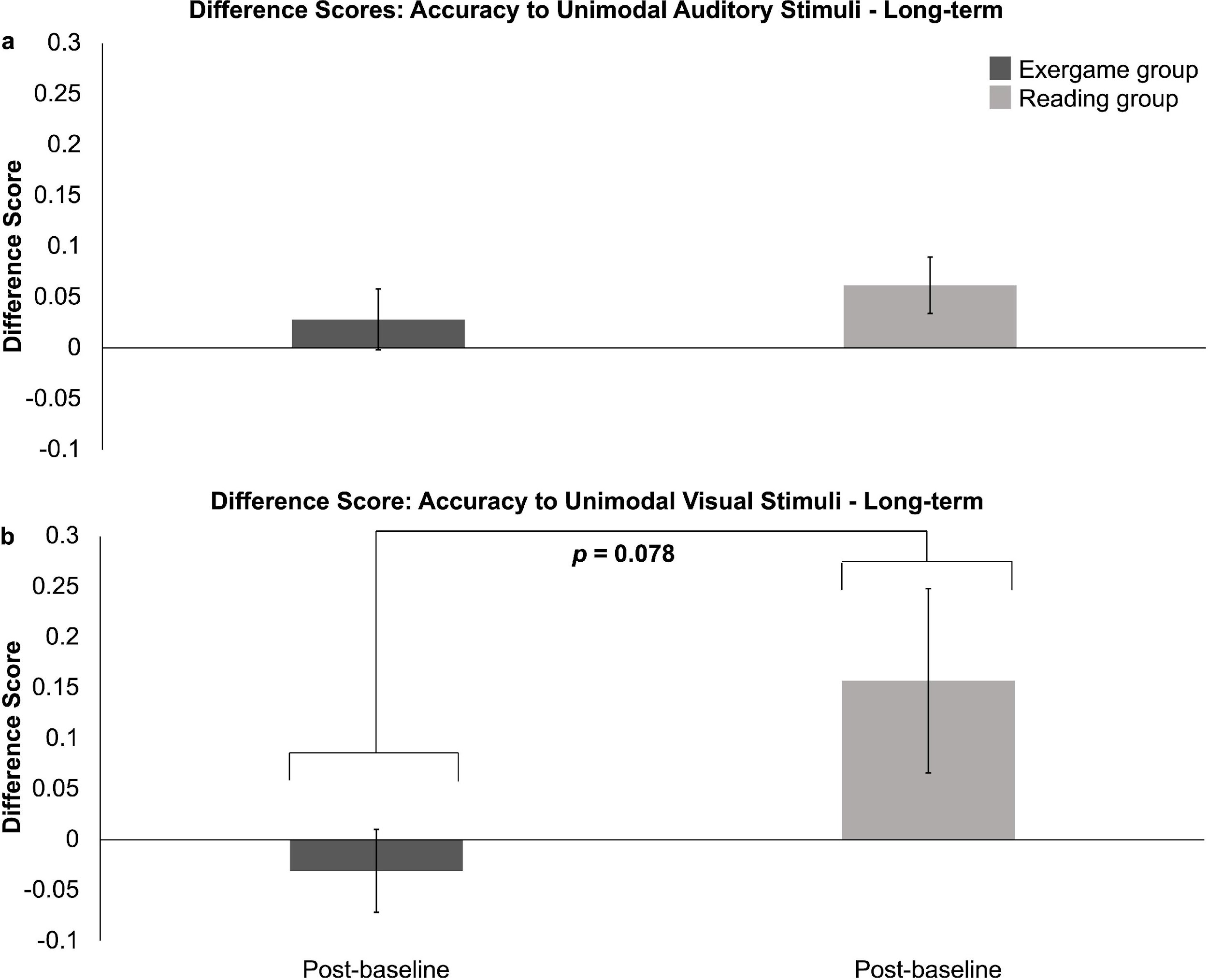


*Supplementary Figure 10:* Difference scores (post-intervention minus baseline accuracy) for auditory (panel a) and visual (panel b) conditions for the Sound-induced Flash Illusion. Those who engaged in physical activity are shown in dark grey and the reading group in light grey. A nearly significant accuracy difference was found in the reading group for the long-term visual condition (*p* = 0.078; panel b). No significant differences were observed in the auditory condition. Error bars represent SEM.


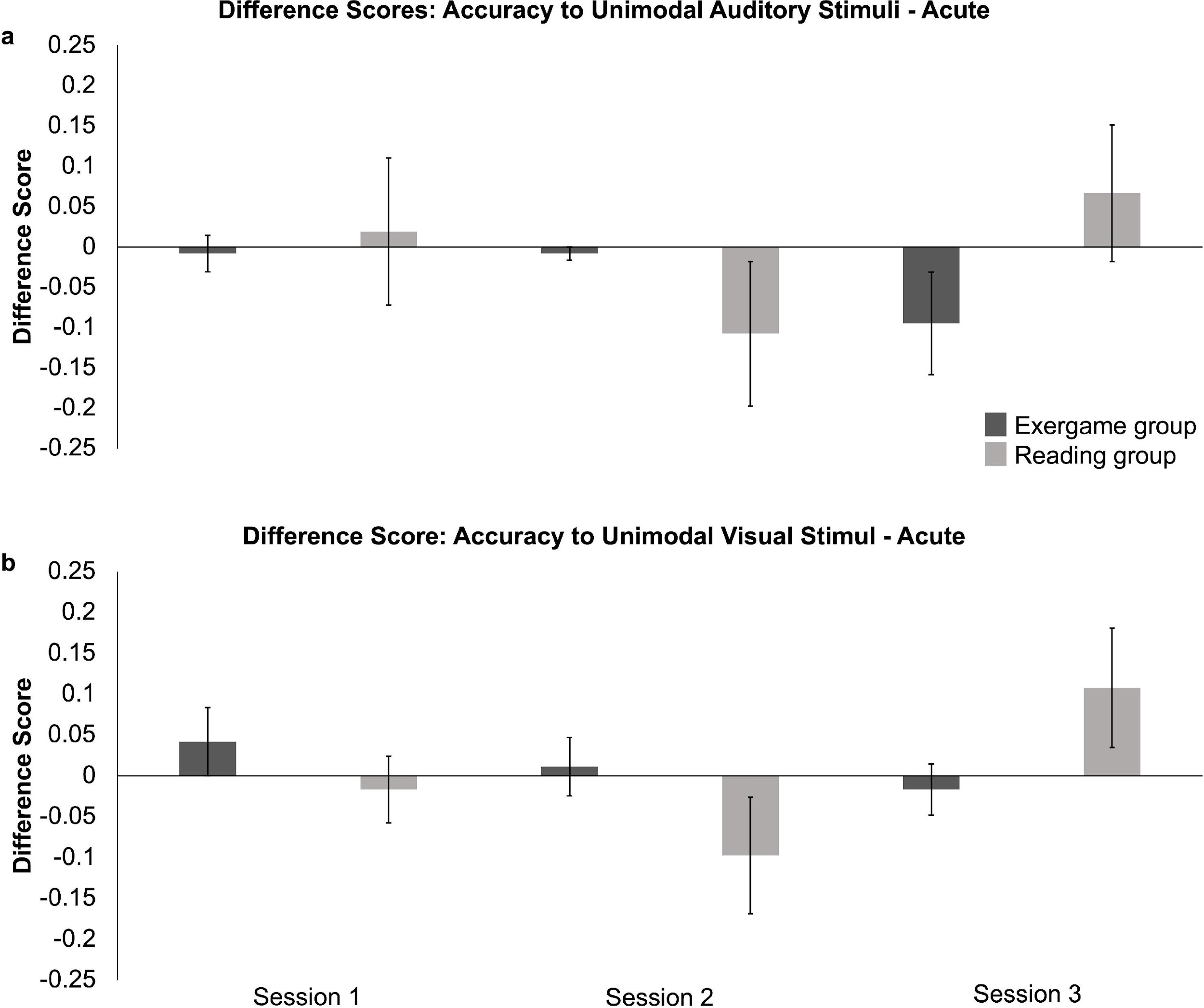


*Supplementary Figure 11*: Difference scores (post-session minus pre-session accuracy) for sessions 1, 2, and 3 in auditory (panel a) and visual (panel b) conditions for the Sound-induced Flash Illusion. Those who engaged in physical activity are shown in dark grey and those in the reading group in light grey. No significant differences were detected in either auditory or visual conditions. Error bars represent SEM.


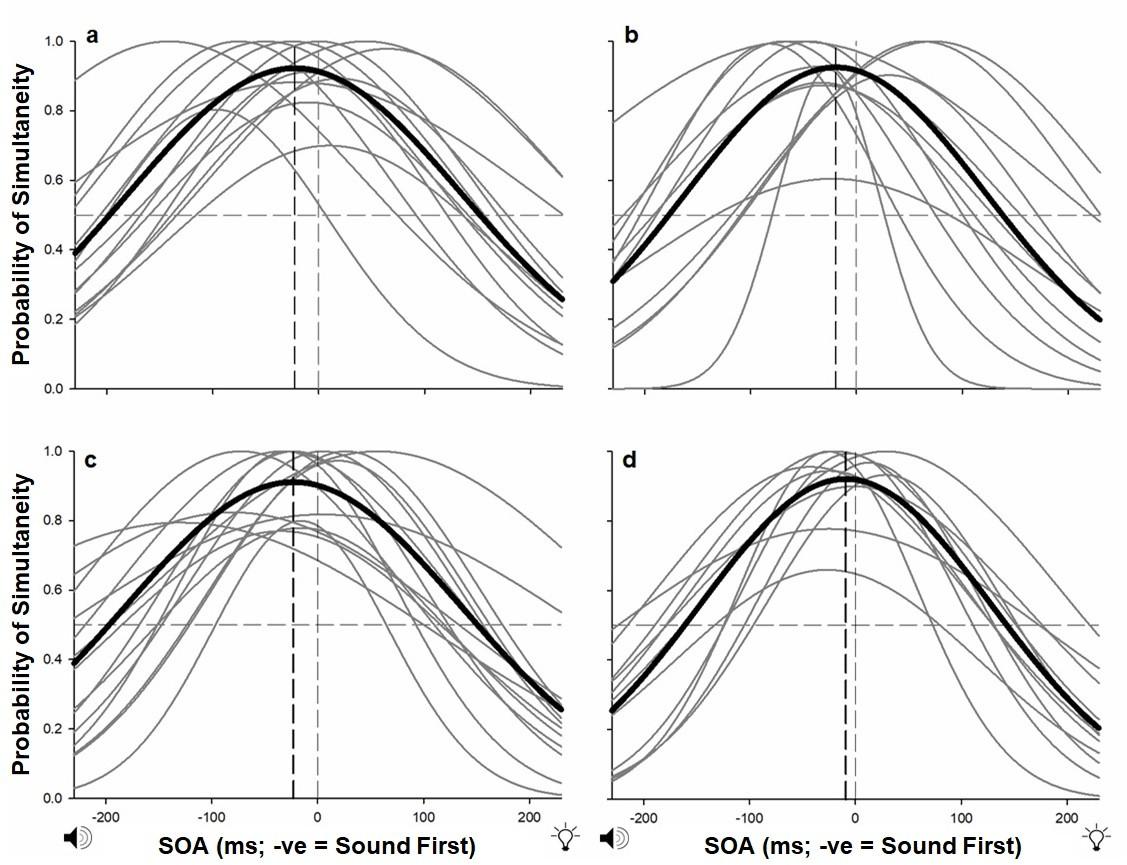


*Supplementary Figure 12:* Gaussian functions fitted to average (thick lines) and individual (thin lines) Simultaneity Judgement data for physical activity (a and c) and reading (b and d) groups. Panels A and B show pre-intervention data for physical activity and reading groups, respectively, while panels C and D show post-intervention data. At baseline, participants who engaged in physical activity required sound 22 ms before light for simultaneity, shifting slightly closer post-intervention (19 ms). Their TBWs decreased from 158.13 (baseline) to 142.24 (post-intervention). Reading participants required sound 23 ms before light at baseline and 9 ms post-intervention. Their TBWs also decreased from 158.73 (baseline) to 137.83 (post-intervention). No significant amplitude differences were observed.


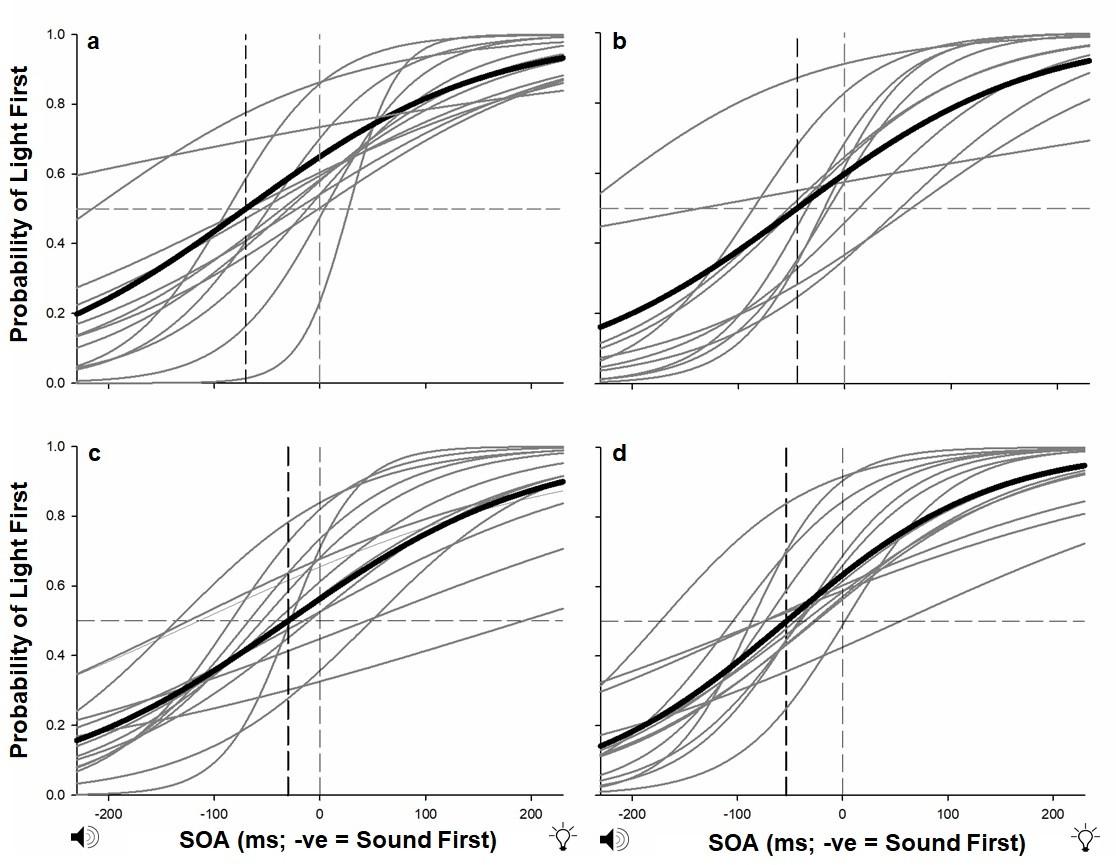


*Supplementary Figure 13:* Sigmoidal functions fitted to average (thick lines) and individual (thin lines) Temporal Order Judgment data. Panels A and B display pre-intervention data for physical activity and reading groups, respectively, while panels C and D present post-intervention data. At baseline, those who engaged in physical activity required sound 70 ms before light for simultaneity, shifting closer post-intervention (45 ms). Their TBWs slightly decreased from 114.06 (baseline) to 112.17 (post-intervention). Reading participants required sound 30 ms before light at baseline and 54 ms post-intervention. Their TBWs decreased from 118.92 (baseline) to 97.82 (post-intervention).


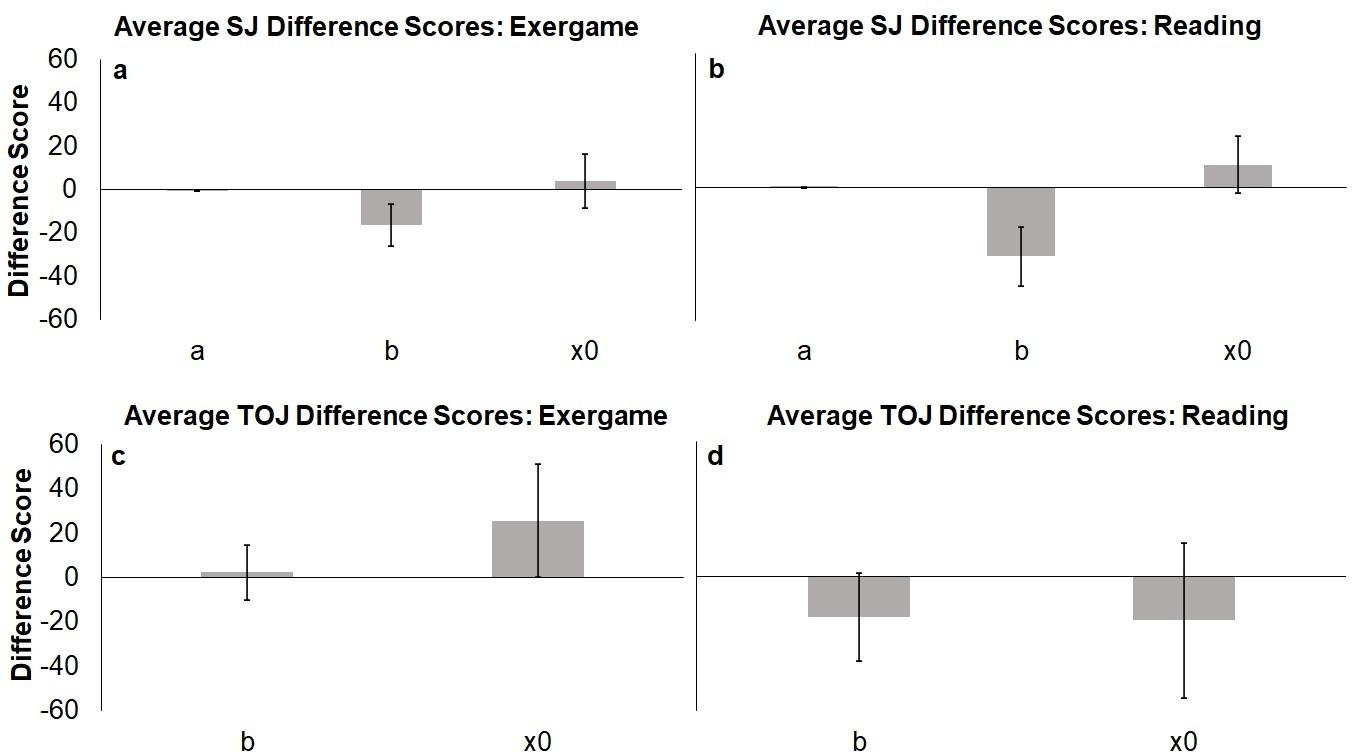


Supplementary *Figure 14:* Difference scores for temporal binding window (TBW) and point of subjective simultaneity (PSS; subtracting baseline from post-intervention values) for Simultaneity Judgement (SJ) and Temporal Order Judgment (TOJ) tasks. Panels A and B show SJ task scores for physical activity and reading groups, respectively. Panels C and D display TOJ task scores for physical activity and reading groups, respectively. No significant effects or interactions were found for TBW and PSS. Amplitude differences between reading (mean = 0.016; s.e. = 0.029) and physical activity (mean = -0.006; s.e. = 0.022) groups were small and not significant. Supplementary Figure 10 provides a graphical representation of these difference scores. Error bars represent SEM.


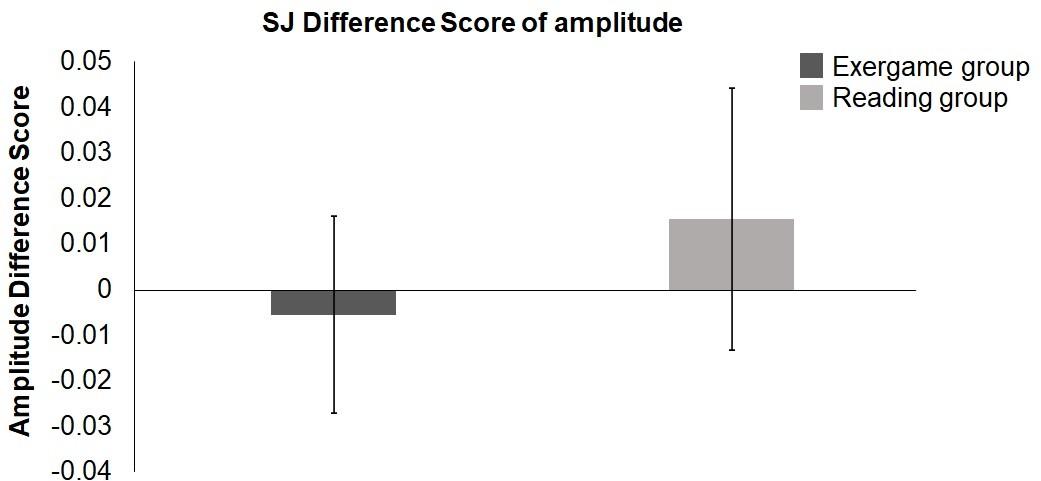


Supplementary *Figure 13:* Amplitude difference scores for Simultaneity Judgement (SJ) task (subtracting baseline from post-intervention values). Dark grey bars represent physical activity group scores, while light grey bars show reading group scores. No significant group effect was found. Error bars indicate SEM.


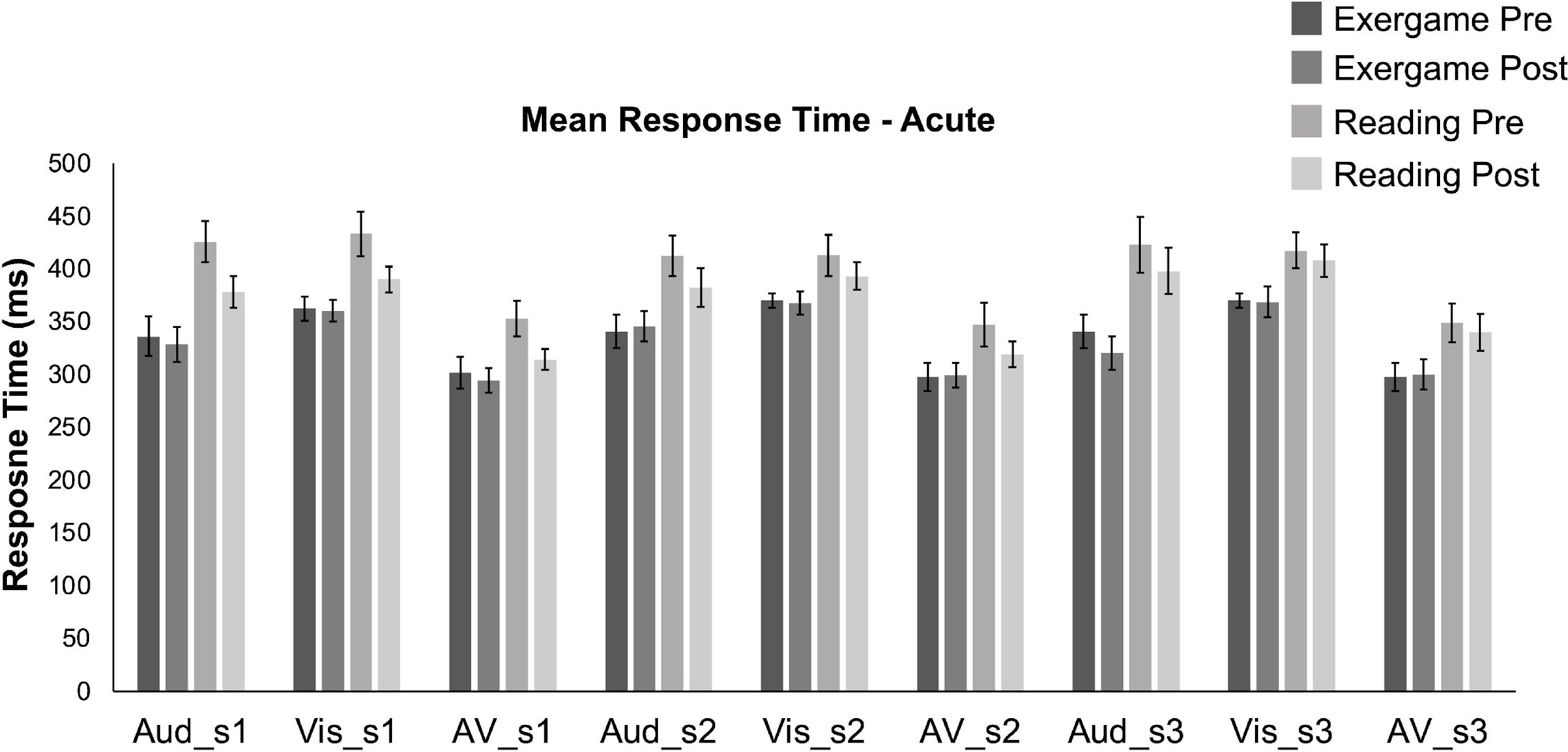


Supplementary *Figure 16:* This figure shows the mean response time from sessions 1, 2, and 3 (dark shades represent pre-session and light shades represent post-session response times) for both the physical activity (dark grey) and reading (light grey) groups across audiovisual, auditory, and visual trials of the Response Time (RT) task. The reading group displayed longer response times (mean = 383.088, s.e. = 8.841) compared to the physical activity group (mean = 333.376, s.e. = 6.880, *p* = 0.006). Additionally, response times for audiovisual stimuli (mean = 317.061, s.e. = 5.842) were significantly faster than auditory (mean = 368.933, s.e. = 9.781; *p* < 0.001) and visual (mean = 387.108, s.e. = 6.6335; *p* < 0.001) modalities. Note: ‘S’ = session, ‘E’ = physical activity group, and ‘R’ = reading group. Error bars indicate the SEM.


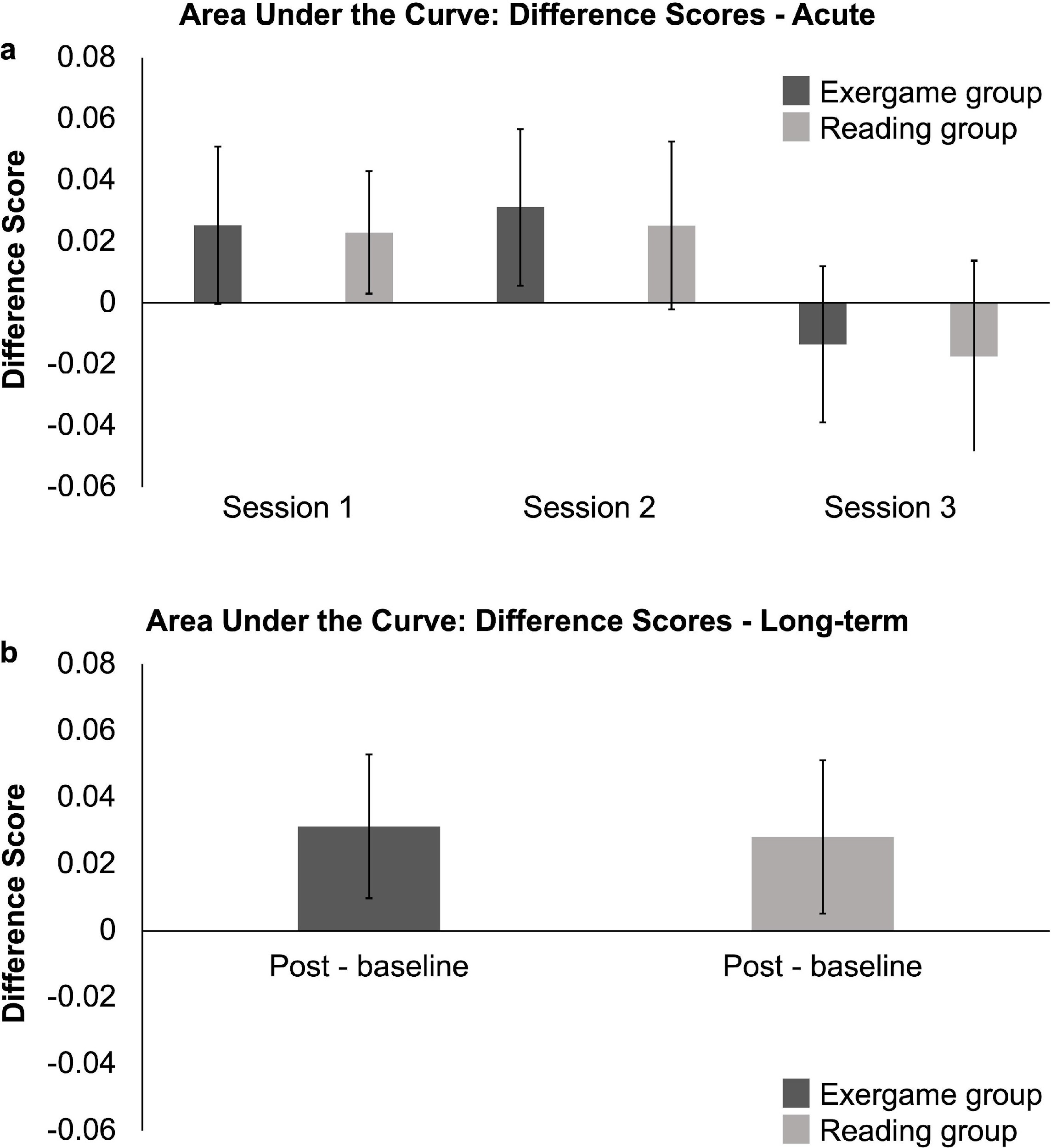


Supplementary *Figure 17:* This figure shows the difference scores calculated by subtracting pre-session AUC scores from post-session AUC scores obtained from the Response Time task. The AUC difference scores represented are from 3 times (session 1 post - pre, session 2 post - pre, and session 3 post - pre) in panel 'a' and 1 time (post - baseline) in panel 'b'. Scores from the physical activity group are shown in dark grey, while those from the reading group are in light grey. No significant effects or interactions were found for both the acute and long-term analyses. Error bars indicate the SEM.

**Median Response Time**

Median response time values were computed, and a 2 (group) x 2 (time) x 3 (modality) mixed-design ANOVA was conducted to determine the long-term effect of physical activity and reading on median response time. The analysis revealed a main effect of group (*F*(1, 24) = 6.590, *p* = 0.017; *η2_p_* = 0.215), a main effect of modality (*F*(1.4126, 33.897) = 71.377, *p* < 0.001; *η2_p_* = 0.748), and a significant interaction between time and modality (*F*(1.598, 38.348) = 3.798, *p* = 0.040; *η2_p_* = 0.137). Pairwise comparisons showed that the physical activity group had faster response times compared to the reading group (p = 0.017). The effect of modality showed that both auditory (*p* < 0.001) and visual (*p* < 0.001) stimuli had significantly longer response times compared to audiovisual stimuli, and visual stimuli exhibited longer response times compared to auditory stimuli (*p* < 0.001). The interaction between modality and time revealed several significant outcomes (see Supplementary Table 3); of interest to this dissertation is the finding that median RTs for audiovisual cues at both baseline and post-intervention were significantly faster compared to baseline auditory and visual median RTs (*p* < 0.01). However, the analysis did not find a main effect of time (*F*(1, 24) = 0.239, *p* = 0.629; *η2_p_* = 0.010). See Supplementary Figure 18 for a graphical representation of the median response times obtained from baseline and post-intervention sessions. This section concludes the analyses used to assess the effects of intervention on median response time, with the following exploratory analyses investigating potential acute changes and difference scores obtained from longitudinal and acute sessions. Just as the main effect of group was investigated for the SIFI and the RT tasks, here too we re-ran these analyses using age as a covariate. When age was added as a covariate, the analysis revealed no main effect of group (*F*(1, 23) = 0.522, *p* = 0.477; *η2_p_* = 0.022). Although we found a near-significant interaction between time and group (*F*(1, 23) = 3.668, *p* = 0.068; *η2_p_* = 0.138), post-hoc analyses did not reveal any significant differences between the conditions. No other effects were significant. Both these results indicate that the faster median response times exhibited by those who engaged in physical activity may be explained by the age difference between the two groups.

A 2 (group) x 6 (time) x 3 (modalities) mixed-design ANOVA was conducted to assess the acute effects of physical activity and reading on median response time. The analysis revealed a main effect of group (*F*(1, 23) = 6.573, *p* = 0.017; *η2_p_* = 0.222), a main effect of time (*F*(2.646, 60.862) = 4.538, *p* = 0.008; *η2_p_* = 0.165), a main effect of modality (*F*(1.276, 29.352) = 43.829, *p* < 0.001; *η2p* = 0.656), and a near-significant interaction between time, modality, and group (F(4.816, 110.768) = 2.036, p = 0.082; *η2_p_* = 0.081). Consistent with mean response time results, pairwise comparisons showed that the reading group had longer median response times compared to the physical activity group (*p* = 0.017). Additionally, both auditory (*p* < 0.001) and visual (*p* < 0.001) stimuli had significantly longer response times compared to audiovisual stimuli, and visual stimuli exhibited longer response times compared to auditory stimuli (*p* = 0.035). The effect of time showed that post-intervention session 1 results had significantly faster response times compared to pre-intervention session 1 results (*p* = 0.004). Finally, the interaction between group, time, and modality revealed multiple significant outcomes (see Supplementary Table 4); however, the overall pattern showed that responses to audiovisual trials were significantly faster across time compared to auditory and visual trials, and the reading group had longer response times compared to those who engaged in physical activity. See Supplementary Figure 19 for a graphical representation of the median response times obtained from sessions 1 through 3. Just as the main effect of group was investigated above, here too we re-ran these analyses using age as a covariate. When age was added as a covariate, the analysis revealed no main effect of group (*F*(1, 222) = 0.652, *p* = 0.652; *η2_p_* = 0.009). We did however find a main effect of age (*F*(1, 222) = 5.726, *p* = 0.026; *η2_p_* = 0.207). No other effects were significant. Similar to the covariate analysis above, these results also indicate that the faster median response times exhibited by the physical activity group above may be explained by the age difference between the two groups.

Additionally, difference scores of the median response time were calculated to assess the long-term and acute effects of physical activity and reading on median response times. A 2 (group) x 3 (modality) mixed-design ANOVA was conducted to assess the long-term effects of intervention on median response time. The results revealed a significant effect of modality (*F*(1.598, 38.348) = 3.798, *p* = 0.040; *η2_p_* = 0.137); pairwise comparisons showed that although audiovisual stimuli were faster than auditory and visual stimuli, the difference obtained from subtracting baseline median RTs from post-intervention median RTs indicated an increase in median response times for audiovisual cues compared to the visual modality (p = 0.040). As such, the improvement in performance was greater for the visual modality compared to the audiovisual modality. The analysis failed to find a significant effect of group (*F*(1, 24) = 0.861, *p* = 0.363; *η2_p_* = 0.035) or an interaction between group and modality (*F*(1.598, 38.348) = 1.241, *p* = 0.298; *η2_p_* = 0.049). Furthermore, a 2 (group) x 3 (time) x 3 (modality) mixed-design ANOVA was conducted to assess acute changes and failed to reveal a significant effect of group (*F*(1, 23) = 3.131, *p* = 0.090; *η2_p_* = 0.169); however, pairwise comparisons showed that the reading group had a larger improvement (i.e., greater difference score) compared to the physical activity group (*p* = 0.090). As Levene's test for Equality of Variance was violated, Friedman tests were conducted, which did not reveal a main effect of time (*χ2* (2) = 4.570, *p* = 0.102) or modality (*χ2* (2) = 3.315, *p* = 0.191). See Supplementary Figure 20 for a graphical representation of the difference scores obtained for auditory, visual, and audiovisual conditions for both acute (panel a) and long-term (panel b) sessions.


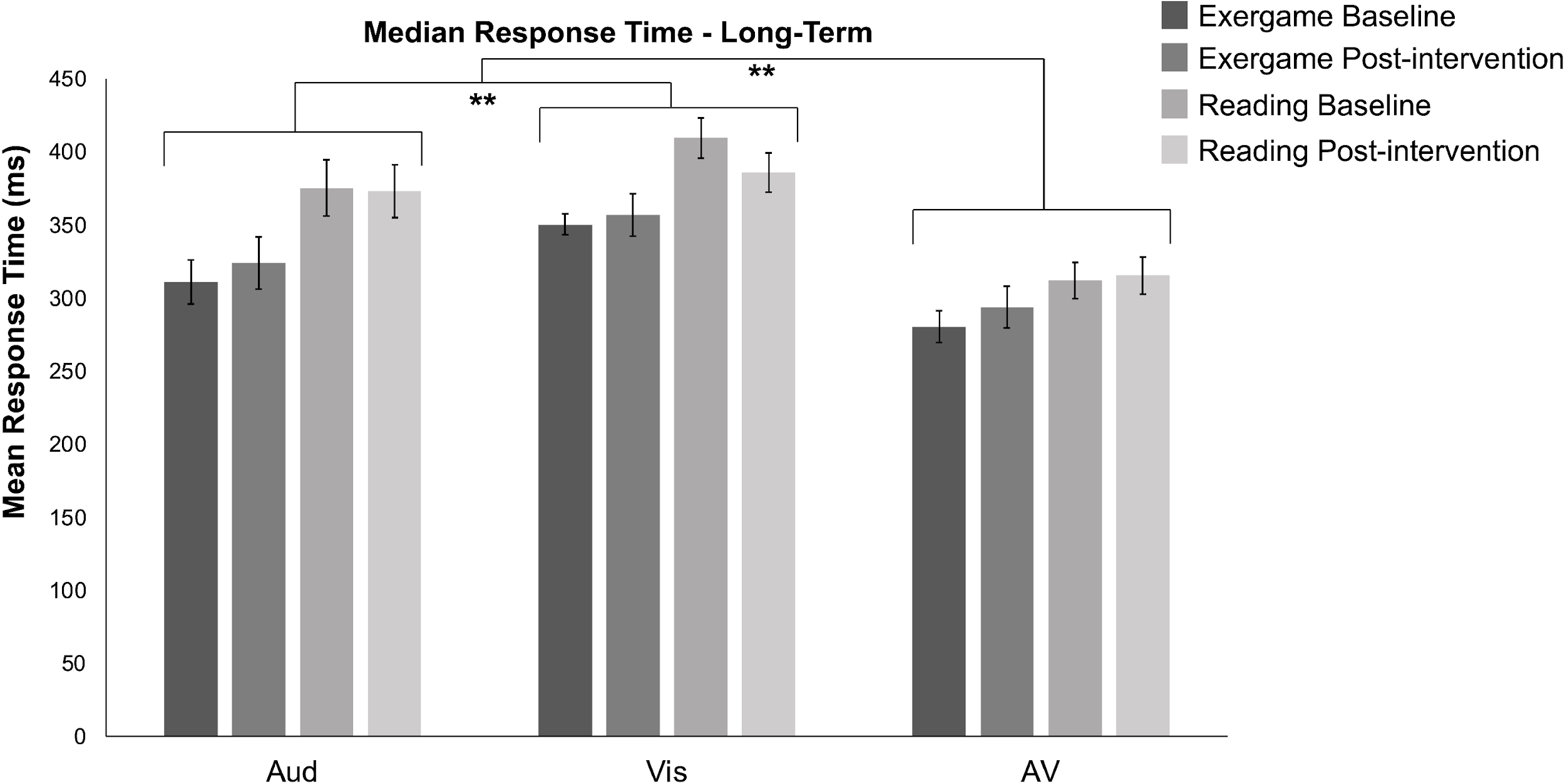


*Supplementary Figure 18:* Median response times from baseline (darker shade) and post-intervention (lighter shade) sessions for participants in the physical activity (dark grey) and reading (light grey) interventions across audiovisual, auditory, and visual trials of the Response Time (RT) task. The long-term analysis results were consistent with the acute sessions, showing longer median RTs for the reading group (median = 362.041, s.e. = 16.10) compared to the physical activity group (median = 319.641, s.e. = 12.404; *p* = 0.017). Additionally, median response times for audiovisual stimuli (median = 300.691, s.e. = 8.161) were significantly faster compared to auditory (median = 346.016, s.e. = 16.573; *p* < 0.001) and visual (median = 375.816, s.e. = 13.670; *p* < 0.001) modalities. Responses to visual stimuli were significantly longer compared to auditory stimuli (*p* < 0.001). Moreover, compared to baseline auditory and visual median RTs, the RTs for audiovisual cues in both baseline and post-intervention sessions were significantly faster (*p* < 0.01). Note: Aud = auditory stimuli, Vis = visual stimuli, AV = audiovisual stimuli, pre = baseline, and post = post-intervention. Error bars represent the SEM.


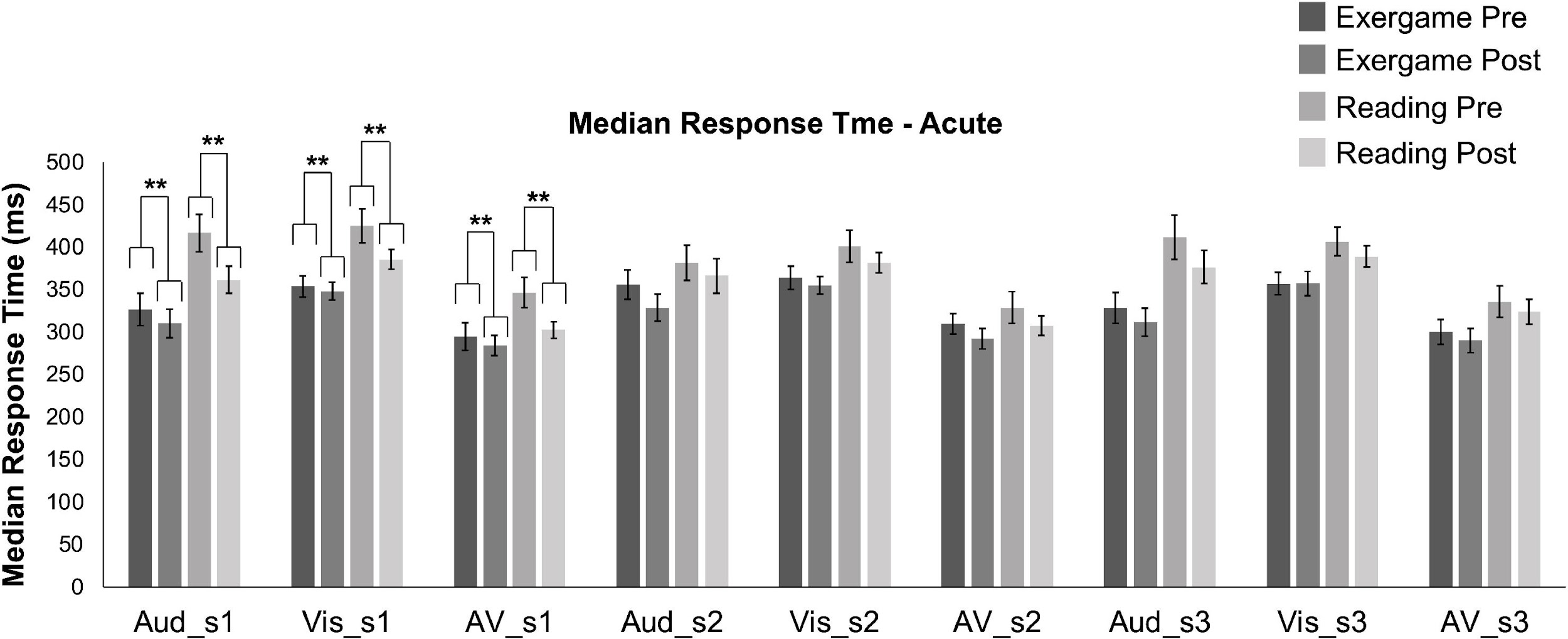


*Supplementary Figure 19:* This figure illustrates the median response times from acute sessions 1, 2, and 3, with dark shades representing pre-session and light shades representing post-session response times. Participants in the physical activity (dark grey) and reading (light grey) interventions are shown for audiovisual, auditory, and visual trials. The reading group exhibited longer median RTs (median = 369.232, s.e. = 8.891) as compared to those who engaged in physical activity (median = 325.926, s.e. = 6.98; *p* = 0.017). Additionally, median response times for audiovisual stimuli (median = 308.936, s.e. = 5.023) were significantly faster compared to auditory (median = 355.161, s.e. = 9.277; *p* < 0.001) and visual (median = 376.117, s.e. = 6.319; *p* < 0.001) modalities. Median responses to visual stimuli were significantly longer than those for auditory stimuli (*p* = 0.035). Furthermore, compared to pre-intervention results from session 1, post-intervention session 1 results showed significantly faster median response times (*p* = 0.004). Note: 'S' = session, 'E' = physical activity group, and 'R' = reading group. Error bars represent the SEM.

| Time*Mod | Time*Mod | Mean Difference | SE | t | Cohen’s d | pbonf |
| --- | --- | --- | --- | --- | --- | --- |
| B, A | B, V | -36.903 | 7.118 | -5.185 | -0.700 | < .001 |
|  | B, AV | 46.743 | 7.118 | 6.567 | 0.887 | < .001 |
|  | PI, AV | 36.528 | 10.162 | 3.595 | 0.693 | 0.010 |
| PI, A | PI, V | -23.160 | 7.118 | -3.254 | -0.440 | 0.026 |
|  | B, AV | 54.164 | 10.162 | 5.330 | 1.028 | < .001 |
| B, V | PI, AV | 43.950 | 7.118 | 6.175 | 0.834 | < .001 |
|  | B, AV | 83.646 | 7.118 | 11.751 | 1.587 | < .001 |
| PI, V | PI, AV | 73.432 | 10.162 | 7.226 | 1.394 | < .001 |
|  | B, AV | 77.324 | 10.162 | 7.609 | 1.467 | < .001 |
|  | PI, AV | 67.110 | 7.118 | 9.428 | 1.274 | < .001 |

*Supplementary Table 3:* Details of post-hoc comparisons for median response times in long-term sessions [baseline (B) and post-intervention (PI)] for the physical activity and reading intervention groups. Results showed that compared to auditory and visual median RTs at baseline, the RTs for audiovisual cues, both during baseline and post-intervention, were significantly faster (*p* < 0.01). Note: Mod = modality, B = baseline, PI = post-intervention, A = audio, V = visual, and AV = audiovisual. Only significant results are presented here.


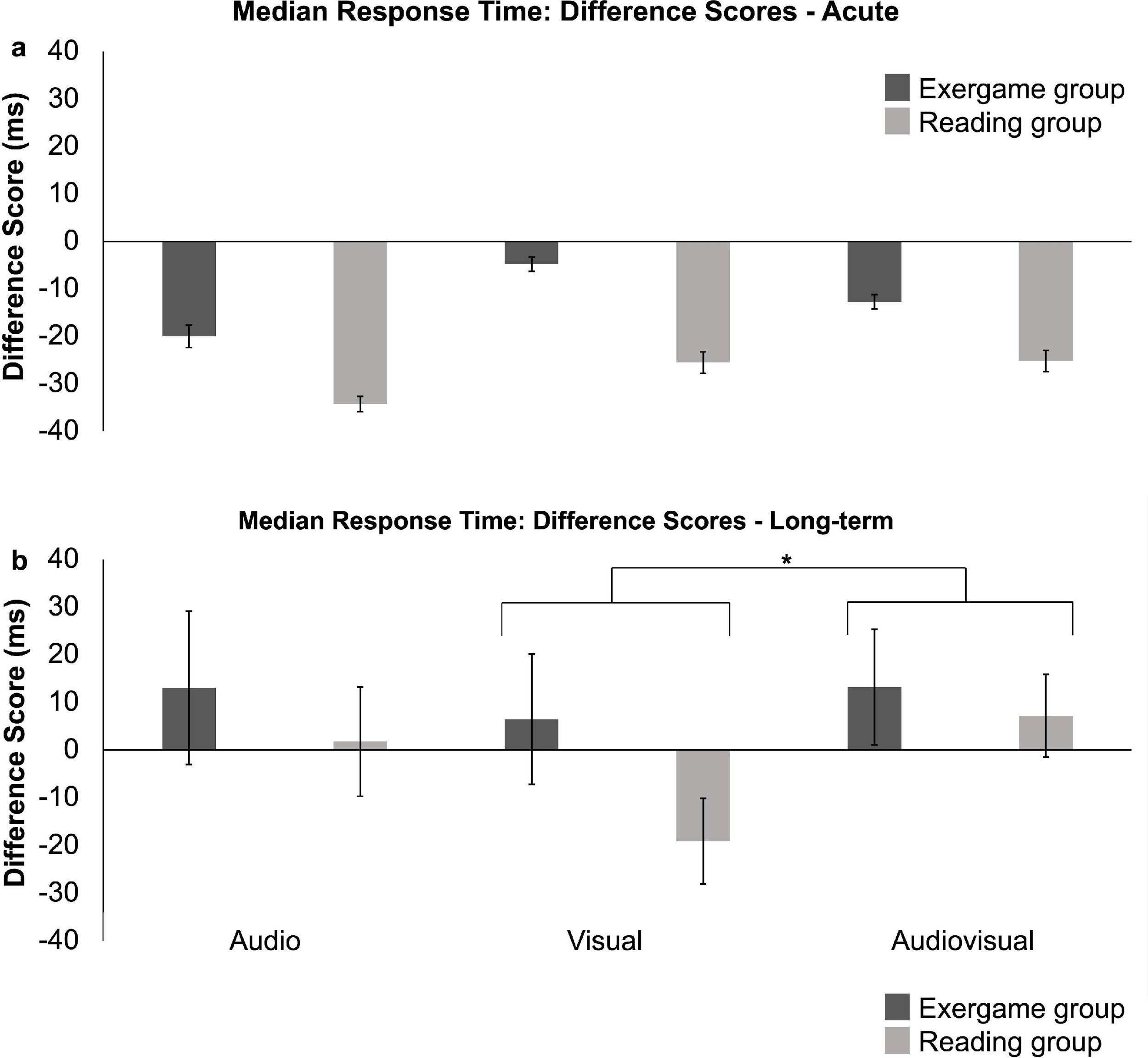


*Figure 20:* This figure presents difference scores calculated by subtracting pre-session response times from post-session response times for auditory, visual, and audiovisual stimuli. The response times shown are collapsed across 3 times (session 1 post - pre session 1, session 2 post - pre session 2, and session 3 post - pre session 3) for acute effects (panel a) and 1 time (post - baseline) for long-term effects (panel b). Scores from the physical activity group are depicted in dark grey, while scores from the reading group are in light grey. For acute effects, although not significant, the reading group showed greater improvement (i.e., larger difference scores) compared to the physical activity group (*p* = 0.090). For long-term effects, analyses revealed that median response times increased after 6 weeks of intervention for audiovisual cues compared to the visual modality (*p* = 0.040). No other significant effects or interactions were observed for both acute and long-term sessions. Error bars represent the SEM.

| Gr*Tm*Mod | Gr*Tm*Mod | Mean Difference | SE | t | Cohen’s d | Pbonf |
| --- | --- | --- | --- | --- | --- | --- |
| G1, S1 pre, A | G2, S1 pre, A | -102.3320 | 23.734 | -4.3120 | -1.7260 | 0.039 |
|  | G1, S1 pre, V | -106.2780 | 23.734 | -4.4780 | -1.7930 | 0.022 |
| G2, S1 pre, A | G1, S1 post, A | 118.612 | 23.734 | 4.998 | 2.001 | 0.003 |
|  | G2, S1 post, A | 63.079 | 13.610 | 4.635 | 1.064 | 0.005 |
|  | G2, S2 post, A | 57.704 | 13.610 | 4.240 | 0.973 | 0.024 |
|  | G1, S3 pre, A | 100.604 | 23.734 | 4.239 | 1.697 | 0.050 |
|  | G1, S3 post, A | 117.331 | 23.734 | 4.944 | 1.979 | 0.004 |
|  | G1, S1 pre, AV | 134.021 | 23.734 | 5.647 | 2.261 | < .001 |
|  | G2, S1 pre, AV | 72.137 | 12.115 | 5.954 | 1.217 | < .001 |
|  | G1, S1 post, AV | 144.809 | 23.734 | 6.101 | 2.442 | < .001 |
|  | G2, S1 post, AV | 123.733 | 17.034 | 7.264 | 2.087 | < .001 |
|  | G1, S2 pre, AV | 119.357 | 23.734 | 5.029 | 2.013 | 0.003 |
|  | G2, S2 pre, AV | 92.417 | 17.034 | 5.426 | 1.559 | < .001 |
|  | G1, S2 post, AV | 136.760 | 23.734 | 5.762 | 2.307 | < .001 |
|  | G2, S2 post, AV | 119.892 | 17.034 | 7.039 | 2.022 | < .001 |
|  | G1, S3 pre, AV | 128.811 | 23.734 | 5.427 | 2.173 | < .001 |
|  | G2, S3 pre, AV | 93.092 | 17.034 | 5.465 | 1.570 | < .001 |
|  | G1, S3 post, AV | 138.928 | 23.734 | 5.854 | 2.343 | < .001 |
|  | G2, S3 post, AV | 103.392 | 17.034 | 6.070 | 1.744 | < .001 |
| G1, S1 post, A | G2, S1 pre, V | -122.5580 | 23.734 | -5.1640 | -2.0670 | 0.002 |
| G2, S1 post, A | G2, S1 post, AV | 60.654 | 12.115 | 5.006 | 1.023 | 0.002 |
| G1, S2 pre, A | G1, S1 post, AV | 71.668 | 16.365 | 4.379 | 1.209 | 0.013 |
| G2, S2 pre, A | G1, S1 post, AV | 107.647 | 23.734 | 4.536 | 1.816 | 0.018 |
|  | G2, S1 post, AV | 86.571 | 17.034 | 5.082 | 1.460 | < .001 |
|  | G2, S2 pre, AV | 55.254 | 12.115 | 4.561 | 0.932 | 0.012 |
|  | G2, S2 post, AV | 82.729 | 17.034 | 4.857 | 1.395 | 0.002 |
|  | G1, S3 post, AV | 101.766 | 23.734 | 4.288 | 1.716 | 0.043 |
| G2, S3 pre, A | G1, S1 pre, AV | 116.388 | 23.734 | 4.904 | 1.963 | 0.005 |
|  | G1, S1 post, AV | 127.176 | 23.734 | 5.358 | 2.145 | < .001 |
|  | G2, S1 post, AV | 106.100 | 17.034 | 6.229 | 1.790 | < .001 |
|  | G1, S2 pre, AV | 101.724 | 23.734 | 4.286 | 1.716 | 0.043 |
|  | G2, S2 pre, AV | 74.783 | 17.034 | 4.390 | 1.261 | 0.012 |
|  | G1, S2 post, AV | 119.127 | 23.734 | 5.019 | 2.009 | 0.003 |
|  | G2, S2 post, AV | 102.258 | 17.034 | 6.003 | 1.725 | < .001 |
|  | G1, S3 pre, AV | 111.178 | 23.734 | 4.684 | 1.875 | 0.011 |
|  | G2, S3 pre, AV | 75.458 | 12.115 | 6.228 | 1.273 | < .001 |
|  | G1, S3 post, AV | 121.295 | 23.734 | 5.111 | 2.046 | 0.002 |
|  | G2, S3 post, AV | 85.758 | 17.034 | 5.035 | 1.446 | < .001 |
| G1, S3 post, A | G2, S1 pre, V | -121.2770 | 23.734 | -5.1100 | -2.0460 | 0.002 |
| G2, S3 post, A | G2, S1 post, AV | 73.321 | 17.034 | 4.304 | 1.237 | 0.018 |
|  | G2, S2 post, AV | 69.479 | 17.034 | 4.079 | 1.172 | 0.044 |
|  | G2, S3 post, AV | 52.979 | 12.115 | 4.373 | 0.894 | 0.024 |
| G1, S1 pre, V | G1, S1 pre, AV | 58.871 | 11.640 | 5.058 | 0.993 | 0.002 |
|  | G1, S1 post, AV | 69.659 | 16.365 | 4.256 | 1.175 | 0.021 |
| G2, S1 pre, V | G1, S1 pre, AV | 137.967 | 23.734 | 5.813 | 2.327 | < .001 |
|  | G2, S1 pre, AV | 76.083 | 12.115 | 6.280 | 1.283 | < .001 |
|  | G1, S1 post, AV | 148.755 | 23.734 | 6.268 | 2.509 | < .001 |
|  | G2, S1 post, AV | 127.679 | 17.034 | 7.496 | 2.154 | < .001 |
|  | G1, S2 pre, AV | 123.303 | 23.734 | 5.195 | 2.080 | 0.002 |
|  | G2, S2 pre, AV | 96.362 | 17.034 | 5.657 | 1.625 | < .001 |
|  | G1, S2 post, AV | 140.706 | 23.734 | 5.928 | 2.373 | < .001 |
|  | G2, S2 post, AV | 123.837 | 17.034 | 7.270 | 2.089 | < .001 |
|  | G1, S3 pre, AV | 132.757 | 23.734 | 5.594 | 2.239 | < .001 |
|  | G2, S3 pre, AV | 97.037 | 17.034 | 5.697 | 1.637 | < .001 |
|  | G1, S3 post, AV | 142.874 | 23.734 | 6.020 | 2.410 | < .001 |
|  | G2, S3 post, AV | 107.337 | 17.034 | 6.302 | 1.810 | < .001 |
| G1, S1 post, V | G1, S1 post, AV | 64.093 | 11.640 | 5.506 | 1.081 | < .001 |
| G2, S1 post, V | G1, S1 post, AV | 101.134 | 23.734 | 4.261 | 1.706 | 0.047 |
|  | G2, S1 post, AV | 80.058 | 12.115 | 6.608 | 1.350 | < .001 |
| G1, S2 pre, V | G1, S1 pre, AV | 68.877 | 16.365 | 4.209 | 1.162 | 0.026 |
|  | G1, S1 post, AV | 79.665 | 16.365 | 4.868 | 1.344 | 0.002 |
|  | G1, S2 pre, AV | 54.213 | 11.640 | 4.657 | 0.914 | 0.008 |
|  | G1, S2 post, AV | 71.616 | 16.365 | 4.376 | 1.208 | 0.013 |
|  | G1, S3 post, AV | 73.784 | 16.365 | 4.509 | 1.245 | 0.008 |
| G2, S2 pre, V | G1, S1 pre, AV | 113.338 | 23.734 | 4.775 | 1.912 | 0.008 |
|  | G1, S1 post, AV | 124.126 | 23.734 | 5.230 | 2.094 | 0.001 |
|  | G2, S1 post, AV | 103.050 | 17.034 | 6.050 | 1.738 | < .001 |
|  | G2, S2 pre, AV | 71.733 | 12.115 | 5.921 | 1.210 | < .001 |
|  | G1, S2 post, AV | 116.077 | 23.734 | 4.891 | 1.958 | 0.005 |
|  | G2, S2 post, AV | 99.208 | 17.034 | 5.824 | 1.673 | < .001 |
|  | G1, S3 pre, AV | 108.128 | 23.734 | 4.556 | 1.824 | 0.017 |
|  | G2, S3 pre, AV | 72.408 | 17.034 | 4.251 | 1.221 | 0.022 |
|  | G1, S3 post, AV | 118.245 | 23.734 | 4.982 | 1.994 | 0.004 |
|  | G2, S3 post, AV | 82.708 | 17.034 | 4.856 | 1.395 | 0.002 |
| G1, S2 post, V | G1, S1 post, AV | 70.567 | 16.365 | 4.312 | 1.190 | 0.017 |
|  | G1, S2 post, AV | 62.517 | 11.640 | 5.371 | 1.054 | < .001 |
| G2, S2 post, V | G2, S1 post, AV | 77.983 | 17.034 | 4.578 | 1.315 | 0.006 |
|  | G2, S2 post, AV | 74.142 | 12.115 | 6.120 | 1.251 | < .001 |
| G1, S3 pre, V | G1, S1 post, AV | 73.038 | 16.365 | 4.463 | 1.232 | 0.009 |
|  | G1, S3 pre, AV | 57.040 | 11.640 | 4.900 | 0.962 | 0.003 |
|  | G1, S3 post, AV | 67.157 | 16.365 | 4.104 | 1.133 | 0.039 |
| G2, S3 pre, V | G1, S1 pre, AV | 111.871 | 23.734 | 4.714 | 1.887 | 0.010 |
|  | G1, S1 post, AV | 122.659 | 23.734 | 5.168 | 2.069 | 0.002 |
|  | G2, S1 post, AV | 101.583 | 17.034 | 5.964 | 1.713 | < .001 |
|  | G2, S2 pre, AV | 70.267 | 17.034 | 4.125 | 1.185 | 0.036 |
|  | G1, S2 post, AV | 114.610 | 23.734 | 4.829 | 1.933 | 0.006 |
|  | G2, S2 post, AV | 97.742 | 17.034 | 5.738 | 1.649 | < .001 |
|  | G1, S3 pre, AV | 106.661 | 23.734 | 4.494 | 1.799 | 0.021 |
|  | G2, S3 pre, AV | 70.942 | 12.115 | 5.856 | 1.197 | < .001 |
|  | G1, S3 post, AV | 116.778 | 23.734 | 4.920 | 1.970 | 0.005 |
|  | G2, S3 post, AV | 81.242 | 17.034 | 4.769 | 1.370 | 0.002 |
| G1, S3 post, V | G1, S1 post, AV | 73.172 | 16.365 | 4.471 | 1.234 | 0.009 |
|  | G1, S3 post, AV | 67.290 | 11.640 | 5.781 | 1.135 | < .001 |
| G2, S3 post, V | G1, S1 post, AV | 105.038 | 23.734 | 4.426 | 1.772 | 0.026 |
|  | G2, S1 post, AV | 83.963 | 17.034 | 4.929 | 1.416 | 0.001 |
|  | G2, S2 post, AV | 80.121 | 17.034 | 4.704 | 1.351 | 0.003 |
|  | G2, S3 post, AV | 63.621 | 12.115 | 5.251 | 1.073 | < .001 |

*Supplementary Table 4:* Details of post-hoc comparisons for median response times in acute sessions (sessions 1, 2, and 3) for the physical activity and reading intervention groups. Results showed that responses to audiovisual trials were significantly faster across time compared to auditory and visual trials, and that participants in the reading group had longer response times compared to those in the physical activity group. Note: Gr = group, Tm = time, Mod = modality, G1 = physical activity group, G2 = reading group, A = audio, V = visual, and AV = audiovisual. Only significant results are presented here.
